# How are biomechanical and psychosocial factors associated with injuries across one season in Swedish elite and sub-elite female soccer players?

**DOI:** 10.1186/s13102-026-02084-y

**Published:** 2026-09-17

**Authors:** John Ressman, Philip von Rosen, Eva Rasmussen-Barr, Wilhelmus Johannes Andreas Grooten

**Affiliations:** 1 Department of Neurobiology, Care Sciences and Society, Division of Physiotherapy, Karolinska Institutet, Alfred Nobels Allé 23, Huddinge, Stockholm, 141 83 Sweden; 2 School of Health and Welfare, Dalarna University, Falun, 791 88 Sweden

**Keywords:** Sports, Injuries, Athlete fear avoidance, Hip strength, Biopsychosocial, Rehabilitation

## Abstract

**Background:**

Soccer is a high-intensity contact sport, and the patterns and burden of injuries can vary based on age, sex, and player level. To better understand injury occurrence, it is important to identify biopsychosocial factors that are associated with injuries. Our study aimed to investigate whether various biomechanical and psychosocial factors associate with time-loss injuries and reported injury problems in the dominant and non-dominant leg across one season in elite and sub-elite female soccer players.

**Methods:**

This prospective study followed 244 elite and sub-elite female soccer players (22 years, SD ± 4) from divisions 1–3 over one season. Data on investigated factors were collected at baseline, while injury data were collected every four weeks. Negative binomial regression analyses were used to examine associations between the investigated factors and injury outcomes.

**Results:**

For time-loss injuries involving the dominant leg, higher levels of hip strength were associated with a lower injury incidence in players aged 16–19 years (IRR = 0.95, 95% CI 0.92–0.98), while this association was not observed in the older age groups. Higher levels of fear avoidance were associated with a higher injury incidence (IRR = 1.08, 95% CI 1.03–1.14). For the non-dominant leg, playing in division 2 was associated with a higher injury incidence (IRR = 2.75, 95% CI 1.23–6.16) compared with division 1. Greater ankle dorsiflexion was associated with a lower injury incidence (IRR = 0.91, 95% CI 0.84–0.99), and higher levels of hip strength were associated with a lower injury incidence (IRR = 0.98, 95% CI 0.96–0.99). Regarding injury problems, higher levels of fear avoidance were associated with a higher rate of reported injury problem observations for both the dominant (IRR = 1.04, 95% CI 1.02–1.07) and non-dominant leg (IRR = 1.04, 95% CI 1.02–1.06).

**Conclusion:**

Biomechanical factors, including hip strength, and psychosocial factors such as fear-avoidance behaviour were associated with time-loss injuries and reported injury problems in female elite and sub-elite soccer players. The separate leg-specific analyses showed different patterns of associations in the dominant and non-dominant leg. Given the relatively small effect sizes, the findings should be considered hypothesis-generating and their clinical relevance requires further investigation.

**Trial registration:**

Retrospectively registered in Clinical Trials Gov, date of registration 13/03/2022. Clinical trials identifier NCT05289284.

**Supplementary Information:**

The online version contains supplementary material available at https://doi.org/10.1186/s13102-026-02084-y.

## Background

Soccer is a high-intensity contact sport, and the patterns and burden of injuries can vary based on age, sex, and player level [1–4]. Despite this, female soccer players have been more scarcely investigated compared to male soccer players [5].

Recent meta-analyses found the injury incidence rates (IIR) for elite senior women’s soccer players to be 5.63–6.7 per 1000 h for overall activities, but match IIRs were 4–7 times higher [5, 6]. While studies on amateur players are rarer, they reported higher overall IIRs of 9.6–12.1 per 1000 h compared to the elite players [1, 6]. The most common injury locations reported are the thigh, knee and ankle [1, 2, 5]. In comparison to time-loss injuries, the prevalence of overuse injuries, defined as an “*injury with insidious onset and no known trauma*” [7], have been reported to range from 16 to 66% of all injuries among female soccer players [1, 5, 8, 9]. This injury type is more commonly seen during the preseason and mostly affects the knee [9, 10].

To prevent injuries and improve player availability, the identification of both intrinsic and extrinsic biopsychosocial risk factors has been pointed out as one of the cornerstones in sports medicine [11–13]. Furthermore, research in general [14, 15] and in sports medicine [16, 17] urges a more holistic biopsychosocial perspective in rehabilitation and injury prevention. As such, psychosocial variables are often overlooked, even though they are suggested to prevent injuries and appear to be efficient in reducing sports injuries in injury prevention programs [16, 18, 19].

In sport injury surveillance, it is recommended to report time-loss injuries in severity categories with the following time bins: 0, 1–7 days, 8–28 days and > 28 days [20]. However, it is common that players continue to play despite pain from a previous acute injury or an overuse injury [21, 22]. It is therefore recommended to report such injury problems with the Oslo Sport Trauma Research Centre (OSTRC) questionnaires on overuse injury or health problems [21]. Both questionnaires offer a severity score from 0 (no injury problem) to 100 (a time-loss injury). The severity score, however, is an arbitrary number and has not yet been validated as a proxy for injury severity [20, 21], which might contribute to inaccuracies in injury data reporting.

In senior female soccer players, there is today some evidence indicating an association between an increased injury risk and older player age [1, 23–25], a previous injury [1, 23, 26–28], increased joint laxity [23, 25, 29], weak hip abductors [30–33] and increased Body Mass Index (BMI) [23, 26, 27]. Moreover, research indicates that injuries most often occur in the dominant leg [26, 34, 35], defined as the preferred kicking leg [36, 37]. Unfortunately, few studies address risk factors for each leg separately. Whittaker et al. [38] argues that research on female athletes is sparse and mainly focuses on physiological, biological and performance-based risk factors, and only a few studies focus on psychosocial and female-specific risk factors such as menstrual cycle and contraceptive use. Slimani et al. [39] reported that a history of stressors and personality attributes was the main predictor of injury rates among both male and female soccer players aged 14–36 years. In addition, research on senior female soccer players indicates an association between psychosocial risk factors and an increased injury risk [23, 40, 41].

To better understand the causes of injuries and how to prevent them, it is essential to conduct and report studies within the appropriate context. Injury problems (non-time-loss injuries) are highly prevalent, yet they are arbitrarily defined, and the risk of injury might differ between the dominant and non-dominant legs. This indicates a need for further research. Additionally, the absence of a biopsychosocial perspective in studies involving senior female soccer players calls for a more comprehensive research approach. Therefore, the present study aimed to investigate whether various biomechanical and psychosocial factors associate with time-loss injuries and reported injury problems in the dominant and non-dominant leg across one season in elite and sub-elite female soccer players.

## Method

### Study design and participants

The present prospective study is part of a previously reported cross-sectional study on elite and sub-elite female soccer players [42], where enrolment of participants and data were collected from January to February 2022. The cross-sectional and prospective project was registered at the United States National Library of Medicine, Clinical Trials Gov [43] 13/03/2022 with the clinical trials identifier: NCT05289284, and was conducted and reported in accordance with the STROBE (Strengthening the Reporting of Observational Studies in Epidemiology) guidelines [44]. Twenty female soccer teams from the three highest divisions in the Swedish soccer league were invited. Those who agreed to participate were screened for demographics, biomechanical and psychosocial factors. Inclusion criteria were players 16 years or older, the ability to understand written and spoken Swedish, and that the players were contracted with a club in one of the three highest divisions in Sweden during the season. Exclusion criteria were two-footed players, players suffering from an ongoing injury that made it impossible to perform the physical tests at baseline without pain, players who had an ongoing injury at baseline that prevented them from playing during the season and players who changed their division level during the season. Fifteen players were excluded from the baseline [42] and a further ten players were excluded in the present prospective study as they declined the invitation (*n* = 3), had an ongoing injury that prevented them from playing during the season (*n* = 6), or changed their division level during the season (*n* = 1). Written informed consent was obtained from all individual subjects to participate in the study. The study was approved by the Regional Ethical Review Board in Stockholm: Ethical approval Dnr 2021–03067 with amendment Dnr 2021-05398-02.

### Data collection

Demographic data, psychosocial and biomechanical measures were collected at baseline by one examiner (JR) with 25 years of experience as a physiotherapist in sports medicine. The procedure is described elsewhere [42].

Prospective injury data were collected using web-based surveys (SurveyMonkey©) sent by e-mail to the participants every fourth week over ten months, starting from the participants’ baseline test. If no response was registered, the participants received a reminder two days later; if there was still no response, participants were contacted by a text message two days later. Regardless of response or non-response, the participants were contacted as usual next month. The questionnaire used for injury registration was based on the translated Swedish version [45] of the Oslo Sports Trauma Research Centre (OSTRC) Overuse Injury Questionnaire [21, 46]. The OSTRC Overuse Injury Questionnaire has shown acceptable content and construct validity with a Cronbach’s α of 0.91 and an Intraclass Correlation Coefficient (ICC) reliability of 0.62 [47].

### Injury definition and classification

The OSTRC questionnaire used in the present study (see Additional file 1) collected data from five different anatomical areas: the lumbar spine/glutes, the lower abdomen/groin/hips, the thigh, the knee and the lower leg/foot. The first initial question contained four options. As the latter three options were only relevant for participants who had a problem located in a specific anatomical area, a selected answer for the first option, *“full participation without (anatomical location) problems the last seven days”*, made all further questions redundant. In this case, a total severity score of 0 was assigned and that anatomical area was completed. If a participant selected the last option, *“could not participate due to (anatomical location) problems the last seven days”*, the first three options were redundant, and a total severity score of 100 was assigned. The participant was then directed to additional questions about the specific anatomical area of the injury and how many days they had abstained from training/match in the last four weeks. This time-loss from training and competition was registered as a time-loss injury (TLI) and defined as recommended by the International Olympic Committee Consensus Statement [20] with the following time intervals: 0, 1–7 days, 8–28 days, > 28 days. If the participant selected the two middle options, *“full participation*,* but with (anatomical location) problems the last seven days” or “reduced participation due to (anatomical location) problems the last seven days”*, they were assigned 8 or 17 points on the severity score and directed to three further questions about training/competition modification, performance and pain. Each question adds 0–25 points. This type of injury problem (IP) is defined as a participant who suffered from training/competition modifications, affected performance and pain/discomfort, but still, to some extent, continued to play [21]. The above-described data collection procedure was repeated for each anatomical area.

For the present study, the injury data were divided into counts of a TLI (a total severity score of 100 in one anatomical area) for the last four weeks or an IP (a total severity score of 8–92 in one or more anatomical areas) for the last seven days. To register a TLI, the participant had to be injured at the time of reporting and the previous seven days, i.e. any shorter or resolved TLI since the foregoing data collection might have been missed. For one period of four weeks or seven days, it was possible to register multiple IPs and TLIs. However, in those cases where two TLIs were registered for the same month and the total time of time-loss exceeded more than 30 days, the time-loss was adjusted to 30 days. A TLI relapse was registered as a second injury if the participant was first registered as 0–92 on the severity scale during the following period of four weeks. In contrast, no distinction was made for an IP regarding its onset and resolution, therefore repeated reports could also represent persistent or recurrent injury problems rather than distinct new episodes. Regarding a TLI, an average value of days of absence for each time bin was estimated and used to calculate exposure/days at risk.

### Outcomes, investigated factors and covariates

#### Outcomes

The outcomes were based on counts of time-loss injuries and reported injury problems. Exposure, or days at risk, was defined as the total number of days an individual was exposed to the possibility of getting injured. In the case of a non-response regarding the OSTRC questionnaire for one month, 30 days of absence were registered and used to adjust the exposure. Statistically, exposure was calculated as the total number of days a participant was registered in the study (10 months, i.e. 300 days at risk) minus those days they registered a time-loss injury or a non-response (30 days of absence). For example, if a participant registered one week of time-loss injury and missed one monthly injury registration, that participant would have a total of 263 days at risk. This way of calculating exposure in days at risk has previously been used to study injuries in college athletes [48, 49] and were selected based on the assumption that athletes routinely perform strength training, conditioning and other potentially injurious sport-related activities outside the team. Since no specific data were collected regarding absence due to disease, this could not be adjusted for in terms of exposure, and as no specific data on training and match exposure were available, metrics such as injuries per 1000 h of training exposure could not be calculated.

#### Investigated factors and covariates

The choice of investigated factors was based on common practice, clinical experience and previous research [50]. Three biomechanical and four psychosocial factors were examined for inclusion in the final models: the Single Leg Squat (SLS) test, ankle dorsiflexion (ADF), hip strength, perceived stress (PSS-14), general anxiety (GAD-7), sleep quality (PSQI) and fear avoidance behaviour (AFAQ).

##### Biomechanical factors

The SLS test was used to evaluate the movement quality and alignment of the foot, knee, pelvic and trunk [51]. The SLS was assessed as pass/fail on a total score for all separate segments in the lower extremity and trunk [42]. To measure ADF, the weight-bearing lunge test was used, which calculates the ADF in degrees by a trigonometric function [52]. Hip strength was measured with a handheld dynamometer (MicroFET2TM wireless, Hoggan Scientific, LLC. USA) with the player performing an isometric clamshell (CLAM) test [53] measured in Nm/(Kg*m)*100. Ankle dorsiflexion and hip strength were used as continuous variables, while the SLS test was used as a categorical variable.

##### Psychosocial factors

General perceived stress was measured using the Swedish version of the Perceived Stress Scale − 14 items **(**PSS- 14) [54] which contains 14 items and a total score that ranges from 0 to 56, where 56 represents high stress [54, 55]. Sleep quality was measured with the Pittsburgh Sleep Quality Index (PSQI) [56] which contains 19 items and a total global score that ranges from 0 to 21, where 21 represents poor sleep [56, 57]. Anxiety was measured with the Swedish version of the Generalized Anxiety Disorder-7 items (GAD-7) scale [58], which contains seven items and ranges from 0 to 21, where 21 represents high anxiety [58, 59]. Fear of avoidance was measured with the Athlete Fear Avoidance Questionnaire (AFAQ). It contains 10 items and ranges from 10 to 50, where 50 represents a high fear of avoidance [60]. AFAQ and PSS-14 were analysed as continuous variables. PSQI was categorised according to the established cut-off (≤ 5 good sleepers/≥6 poor sleepers), while GAD-7 was categorised according to established severity cut-offs (0–4 no/minimal anxiety, 5–9 mild anxiety, and ≥ 10 moderate/severe anxiety). The moderate and severe GAD-7 categories were combined due to sparse observations in the severe category.

Other covariates used in the analysis were: division level (division 1, 2, 3) and Body Mass Index (BMI), where BMI was used as a continuous variable. Age was categorised into three groups (16–19, 20–24, and 25–39 years) because evaluation of the linear relationship with the outcomes on the log scale indicated that the assumption of linearity was not consistently supported across the leg-specific outcomes. Age was always included as an a priori variable.

### Statistical analysis

To check for normality, demographic data were visually analysed with histograms and distributional diagnostic plots and tested for skewness and kurtosis [61]. As not all demographic data were normally distributed, descriptive data were expressed as medians and minimum/maximum values. Before any calculation, all data were categorised as dominant and non-dominant leg, and the dominant leg was defined as the preferred kicking leg [36, 37].

The dominant and non-dominant legs were analysed separately to investigate leg-specific associations between biomechanical and psychosocial factors and injury outcomes. This approach allowed side-specific biomechanical measures and injury outcomes to be evaluated within the corresponding leg. The Wilcoxon signed rank test for paired non-normally distributed data was used to analyse the difference between the dominant and non-dominant leg for hip strength, ankle dorsiflexion, time-loss injuries and injury problems. For time-loss injuries, injury incidence rates (IIRs), season (period) prevalence, and injuries per player-season were calculated. IIRs were calculated as the total number of time-loss injuries divided by the total number of exposure days ×1000, season prevalence was calculated as the number of players with at least one time-loss injury divided by the total number of players ×100, and injuries per player-season were calculated as the total number of time-loss injuries divided by the total number of players (see Table 3)**.** For injury problems, the total number of positive OSTRC reports for the five anatomical areas was recorded as reported injury problem observations. Because repeated positive reports may represent continuation or recurrence of an existing problem rather than distinct new injury episodes, these observations were not interpreted as incident injury problems. Cumulative season prevalence of reported injury problems was calculated as the number of players with one or more positive OSTRC reports for any anatomical area during the season divided by the total number of players ×100. The number of reported injury problem observations per player-season was calculated as the total number of positive anatomical-area reports divided by the total number of players (see Table 3)**.**

As a method of model-building, “a purposeful selection of covariates strategy” was used [62] to build four multivariate models for the dominant and non-dominant leg. As a first step, all investigated factors and covariates were evaluated for outliers, missing data and their relation to the outcome with a Poisson regression. The outcome variables were evaluated for zero inflation [63], and the offset variable (exposure) was evaluated for different time intervals. As no difference in incidence rate ratio (IRR) was detected when the smallest or highest value for a time-loss injury was seen for the intervals 1–7 days and 8–28 days, an average value was calculated for those intervals. For the time interval > 28 days, 30 days were registered. To ascertain that the basic assumption for conducting a Poisson regression was met, the data were then checked for linearity on the log scale, multicollinearity and overdispersion [62–64]. The control of multicollinearity was performed for all variables, both categorical and continuous, with an overall correlation statistic and with the specific variance inflation factor (VIF) command in the software program STATA. As there was obvious overdispersion and the data contained repeated measurements per individual, thereby violating the assumption of independent observations, all models used a negative binomial regression and were estimated with cluster-robust standard errors at the individual level [63]. Finally, all investigated factors and covariates were analysed one by one with a single univariate negative binomial regression with cluster-robust standard errors.

In a second step, the “purposeful selection of covariates strategy” [62] started with a full base model, which contained all investigated factors and covariates identified for inclusion in step 1. The significance level for removal of eligible covariates from the model was set at *p* ≥ 0.20; the results were expressed as incidence rate ratio (IRR) with a 95% confidence interval (95% CI), and the Wald test was used to evaluate the contribution of all investigated factors and covariates to the model. Variables with p-values > 0.05 were considered non-significant and were sequentially removed in the model reduction process [62]. At each step, the reduced model was compared with the previous, larger model. This iterative process was repeated until a stable, parsimonious, and non-overfitted model was obtained. Initial model fit was assessed using several complementary measures, such as Akaike´s information criterion (AIC) and Bayesian information criterion (BIC) and predictive effects were evaluated by comparing the coefficients (IRR) from the full and reduced models [63]. A variable was considered a potential confounder if the removal of that variable changed the IRR of any other variable by ≥ 10%. The remaining variables were tested for possible interactions [62, 63]. Model diagnostics and sensitivity analyses were performed to evaluate the adequacy and fit of the final models, including the identification of potential outliers and influential observations. The overall model specification was finally assessed using the *linktest* procedure in Stata [63, 64]. Furthermore, additional sensitivity analyses were performed for all multivariate models regarding the offset variable (exposure) as a non-response concerning the OSTRC questionnaire for one month automatically assigned the participants 30 days of absence. The sensitivity analyses included the assignment of 15 days of absence, zero days of absence and a complete case analysis where all participants who had at least one non-response during the 10-month period were excluded.

As a rule of thumb, it has been suggested that for every variable screened for association in a regression model, there should be at least ten events [64]. Such guidelines are useful as indicators of potential problems, but should not be applied categorically, as other factors may influence model stability [64], Furthermore, some evidence suggests that this rule may be relaxed to as few as 5–9 events in certain situations [65]. Loss to follow-up was addressed by excluding participants at the time of withdrawal; however, data collected up to that point were retained and included in the analyses. To collect and organise the data, Microsoft Excel for Microsoft 365 MSO (Version 2510 Build 16.0.19328.20190) 64-bit for Windows was used. All statistical analyses were performed in STATA (version 15.1) and the code for the analyses is included in Additional file 2.

## Results

### Participants and injury characteristics

In total, 244 players from soccer divisions 1–3 in Sweden were included in the study. During the 10-month follow-up, the average response rate was 85% (median = 83%), and in total 19 players (8%) dropped out. The reasons for dropping out during the season were that the player stopped playing soccer (*n* = 11), serious injuries and illnesses (*n* = 5), no reason given (*n* = 2) and change transfer to a lower division (*n* = 1). Demographics, biomechanical and psychosocial factors stratified by division are described in Table 1.

**Table 1:** Baseline subject characteristics, stratified by division and presented as medians (min-max)

Characteristics	Total group (*n* = 244)	Division 1 (*n* = 89)	Division 2 (*n* = 49)	Division 3 (*n* = 106)
Age, yr.	22 (16–39)	23 (17–38)	23 (16–31)	19 (16–39)
Height, m	1.70 (1.52–1.83)	1.71 (1.57–1.82)	1.67 (1.52–1.83)	1.69 (1.55–1.83)
Weight, kg	63 (50–85)	64 (55–85)	62 (50–78)	63 (50–83)
BMI^a^, kg/(m*m)	22 (18–28)	23 (19–28)	22 (20–25)	22 (18–27)
Ankle dorsiflexion				
Dominant leg	45°* (32°-56°)	44° (32°-54°)	45° (32°-55°)	45° (36°-56°)
Non-dominant leg	45°* (34°-58°)	46° (35°-53°)	44° (34°-53°)	45° (36°-58°)
Hip strength				
Dominant leg	96* (48–196)	93 (60–172)	102 (62–196)	96 (48–144)
Non-dominant leg	97* (40–204)	97 (40–160)	102 (63–204)	97 (56–149)
AFAQ^b^	23 (10–45)	23 (10–42)	25 (10–42)	21 (10–45)
PSS-14^c^	32 (20–42)	31 (22–42)	33 (22–41)	31 (20–39)
PSQI^d^	5 (0–15)	4 (0–15)	5 (0–15)	5 (1–14)
GAD-7^e^	5 (0–20)	4 (0–20)	6 (0–16)	6 (0–16)

^a^*BMI* Body Mass Index, ^b^*AFAQ* Athlete Fear Avoidance Questionnaire, ^c^*PSS-14* Perceived Stress Scale 14-item, ^d^*PSQI* Pittsburgh Sleep Quality Index, ^e^*GAD-7 *Generalized Anxiety Disorder 7-item scale, *n* denotes the number of subjects in the total group and each division, *Mdn* median, *yr. *years, *m *metres, *kg* kilograms

^*^Denotes a statistically significant difference between legs, p-values at *p* < 0.05

Altogether, 87 time-loss injuries and 1397 injury problems (including multiple injuries) were registered during the season. For injury problems, there was a significant (*p* < 0.05) difference between the dominant leg (DL, *n* = 736) and non-dominant leg (NDL, *n* = 661), but not for time-loss injuries (DL, *n* = 37; NDL, *n* = 50). The two most common injury locations for an injury problem were the foot and the lumbar spine, and for time-loss injury, the knee and the foot. This applied to both the DL and NDL, see Table 2.

**Table 2:** Injury location and frequency for time-loss injuries and injury problems in the dominant and non-dominant leg over a 10-month season

Injury location	Total number of time-loss injuries in the dominant leg^a^	Total number of time-loss injuries in the non-dominant leg^a^
Lumbar^b^	1 (3%, *n* = 1)	1 (2%, *n* = 1)
Groin	7 (20%, *n* = 7)	8 (16%, *n* = 8)
Thigh	3 (8%, *n* = 3)	4 (8%, *n* = 4)
Knee	15 (43%, *n* = 15)	19 (38%, *n* = 18)
Foot	11 (26%, *n* = 9)	18 (36%, *n* = 15)
Total	37 (100%)	50 (100%)

Injury location	Total number of injury problems in the dominant leg^a^	Total number of injury problems in the non-dominant leg^a^
Lumbar^b^	182 (25%, *n* = 85)	185 (28%, *n* = 85)
Groin	115 (16%, *n* = 71)	85 (13%, *n* = 47)
Thigh	101 (14%, *n* = 58)	72 (11%, *n* = 50)
Knee	115 (16%, *n* = 59)	130 (20%, *n* = 57)
Foot	223 (30%, *n* = 96)	189 (29%, *n* = 91)
Total	*736 (100%)	*661 (100%)

^a^*In brackets* Percentage of total injuries, *n *number of subjects with at least one injury, ^b^*Lumbar* Most lumbar-spine problems were registered as left-sided, right-sided, central, bilateral, or combinations of these locations. For participants who registered their problem as central only, the problem was included in both the dominant- and non-dominant-leg, *A time-loss injury* an injury that demanded time-loss from training and competition, *An injury problem* an injury that didn´t demand any time-loss from training and competition

^*^Denotes a statistically significant difference between legs, p-values at *p* < 0.05

For all players, the total exposure (days at risk) during the season was 55,728 days with a median of 266 (1st quartile = 180, 3rd quartile = 296) days per player. For time-loss injuries, the average number of time-loss injuries (DL/NDL) per player-season was 0.4 (SD ± 0.7), the injury incidence was 1.6 (95% CI 1.3–1.9) per 1000 days of exposure, and the total season prevalence was 27.1% (95% CI 21.8–33.0). For injury problems, the average number of reported injury problem observations per player-season (DL/NDL) was 5.7 (SD ± 6.3), the rate of reported injury problem observations was 25.1 (95% CI 23.8–26.4) per 1000 days of exposure, and the cumulative season prevalence of reported injury problems was 84.4% (95% CI 79.3–88.5), see Table 3.

**Table 3:** Descriptive statistics for time-loss injuries and injury problems over a 10-month season

	Total injuries (*n* = 244)^a^	Dominant leg	Non-dominant leg
Average number of time-loss injuries per player-season (mean ± SD (range))	0.4 ± 0.7 (0–3)	0.2 ± 0.4 (0–2)	0.2 ± 0.5 (0–3)
Time-loss injury incidence, per 1000 days of exposure (95% CI)^b^	1.6 (1.3–1.9)	0.7 (0.5–0.9)	0.9 (0.7–1.2)
Season prevalence (95% CI) for time-loss injuries^c^	27.1 (21.8–33.0)	13.5 (9.8–18.4)	17.2 (13.0-22.5)
Average number of reported injury problem observations per player- season (mean ± SD (range))	5.7 ± 6.3 (0–37)	3.0 ± 3.6 (0–22)	2.7 ± 3.3 (0–16)
Rate of reported injury problem observations, per 1000 days of exposure (95% CI)^d^	25.1 (23.8–26.4)	13.2 (12.3–14.2)	11.9 (11.0-12.8)
Cumulative season prevalence (95% CI) of reported injury problems^e^	84.4 (79.3–88.5)	73.8 (67.9–78.9)	74.2 (68.3–79.3)

*SD *standard deviation, *95% CI*  95% confidence interval, *A time-loss injury* an injury that demanded time-loss from training and competition, *An injury problem *an injury that didn´t demand any time-loss from training and competition, ^a^Includes bilateral injuries and all injuries regardless of leg, ^b^Time-loss injury incidence rate = (number of time-loss injuries / total exposure days) ×1000, ^c^Season prevalence of time-loss injuries = (number of players with ≥ 1 time-loss injury / total number of players) ×100, ^d^Rate of reported injury problem observations = (number of positive OSTRC reports / total exposure days) ×1000, ^e^Cumulative season prevalence of reported injury problems = (number of players with ≥ 1 positive OSTRC report / total number of players) ×100

### Multivariate analyses

The univariate Poisson regression analyses for time-loss injuries and injury problems in the dominant (DL) and non-dominant leg (NDL) are reported in Additional files 3 and 4. The multivariate analyses for time-loss injury and injury problems are presented in Tables 4, 5, 6 and 7.

#### Time-loss injury, dominant leg

For the dominant leg, higher levels of hip strength were significantly associated with a 5% lower injury incidence (IRR = 0.95, *p* < 0.01, 95% CI 0.92–0.98) in the age category 16–19 years. The association was not observed in age categories 20–24 years and > 24 years, indicating that the association between hip strength and injury rate differed across age groups (see Table 4). Compared with the youngest age group (16–19 years), the association between hip strength and injury incidence was attenuated in the older age categories (age category 20–24; IRR 1.05, *p* = 0.03, 95% CI 1.01–1.09 and age category > 24; IRR 1.01, *p* = 0.62, 95% CI 0.97–1.06). To aid the interpretation of the interaction between age group and hip strength, age-specific IRRs were estimated with linear combinations of coefficients (lincom) for the two older age groups, see Additional file 5. In the age category 20–24, higher hip strength was not associated with injury incidence (IRR 1.00, *p* = 0.71, 95% CI 0.97–1.01). In contrast, in the > 24 years age category, higher hip strength was significantly associated with a lower injury incidence (IRR 0.96, *p* < 0.01, 95% CI 0.93–0.99).

**Table 4:** Factors associated with the occurrence of time-loss injuries in the dominant leg based on the multivariate analysis

Variables	Time-loss injuries for the dominant leg		
	IRR^a^	95% CI^b^	*p*-value^c^
Age^d^			
16–19	1		
20–24	1.29	0.40–4.16	0.68
> 24	1.32	0.40–4.31	0.65
Hip strength^e^			
CLAM measured in	0.95	0.92–0.98	< 0.01*
Nm/(Kg*m)*100			
Interaction effect			
Age 20–24*hip strength	1.05	1.01–1.09	0.03*
Age > 24*hip strength	1.01	0.97–1.06	0.62
AFAQ^f^			
(No fear/fear; 10–50)	1.08	1.03–1.14	< 0.01*

^a^*IRR* Incidence rate ratio, ^b^*95% CI* 95% Confidence interval, ^c^*P-value* Probability value, ^d^*Age *Main effects of age categories represent differences between age groups at the mean level of hip strength, as hip strength was mean-centered due to its interaction with age, ^e^*Hip strength* Main effect with reference category age 16–19 year, age-specific effects of hip strength were calculated using Lincom, see text for estimates, Side-Lying Clamshell (CLAM), ^f^*AFAQ* Athlete fear avoidance questionnaire

*Denotes a statistically significant p-value at *p* < 0.05

**Fig. 1:**
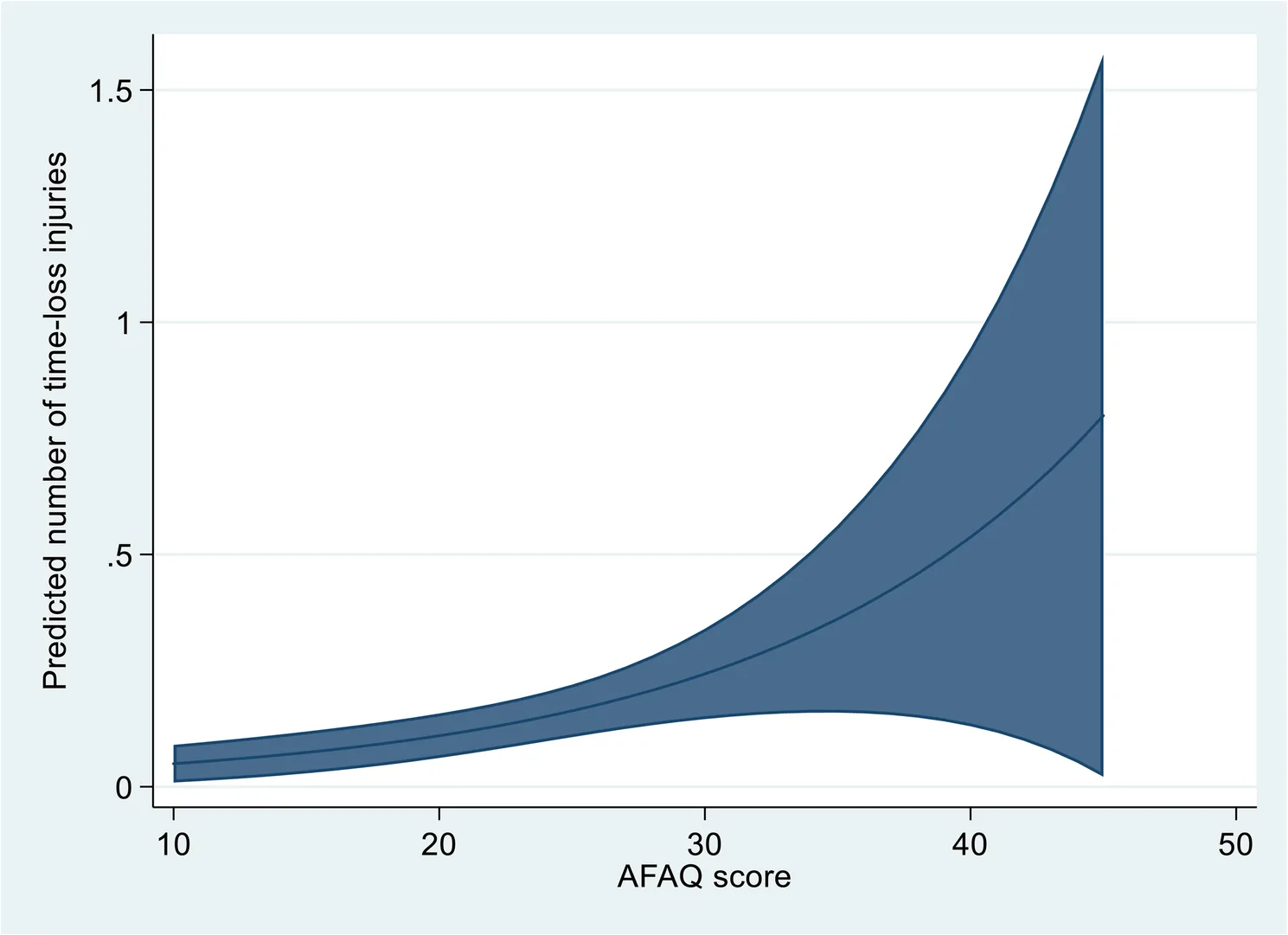
Predicted number of time-loss injuries in the dominant leg by Athlete Fear Avoidance Questionnaire (AFAQ) score, with 95% confidence band across the observed AFAQ range (10–45)

Finally, higher levels on the Athlete Fear Avoidance Questionnaire (AFAQ) were significantly associated with an 8% higher injury incidence per one-point increase in AFAQ score (IRR = 1.08, *p* < 0.01, 95% CI 1.03–1.14), see Table 4. Marginal predictions demonstrated an increasing predicted number of time-loss injuries across the observed AFAQ range, with wider confidence intervals at higher AFAQ scores indicating greater uncertainty in the predictions, see Fig. 1.

#### Time-loss injury, non-dominant leg

For the non-dominant leg, playing at division level 2 was significantly associated with a 175% higher injury incidence compared to division level 1 (IRR = 2.75, *p* < 0.01, 95% CI 1.23–6.16). Ankle dorsiflexion was significantly associated with a 9% lower injury incidence (IRR = 0.91, *p* = 0.02, 95% CI 0.84–0.99), and hip strength was associated with a 2% lower injury incidence (IRR = 0.98, *p* < 0.01, 95% CI 0.96–0.99), see Table 5.

**Table 5:** Factors associated with the occurrence of time-loss injuries in the non-dominant leg based on the multivariate analysis

Variables	Time-loss injuries for the non-dominant leg		
	IRR^a^	95% CI^b^	*p*-value^c^
Age			
16–19	1		
20–24	1.16	0.51–2.63	0.72
> 24	0.73	0.29–1.83	0.50
Division			
1	1		
2	2.75	1.23–6.16	0.01*
3	1.02	0.45–2.29	0.97
Ankle dorsiflexion^d^	0.91	0.84–0.99	0.02*
Hip strength^e^			
CLAM measured in Nm/(Kg*m)*100	0.98	0.96–0.99	< 0.01*

^a^*IRR* Incidence rate ratio, ^b^*95% CI* 95% Confidence interval, ^c^*p-value* Probability value, ^d^*Ankle dorsiflexion* measured with Weight Bearing Dorsiflexion Lunge Test (WBLT) and calculated with a trigonometric dorsiflexion angle (TA), ^e^*Hip strength* measured with Side-Lying Clamshell (CLAM)

*Denotes a statistically significant p-value at *p* < 0.05

#### Injury problem dominant and non-dominant leg

-dominant leg high levels of fear-avoidance were significantly associated with a 4% higher rate of reported injury problem observations, see Tables 6 and 7. Marginal predictions indicated a steady, linear rise in expected injury problem observations across the AFAQ range.

**Table 6:** Factors associated with reported injury problems in the dominant leg based on multivariate analysis

Variables	Reported injury problems for the dominant leg		
	IRR^a^	95% CI^b^	*p*-value^c^
Age			
16–19	1		
20–24	0.99	0.69–1.43	0.97
> 24	1.18	0.80–1.74	0.42
Ankle dorsiflexion^d^	1.04	1.00-1.08	0.07
AFAQ^e^			
(No fear/fear; 10–50)	1.04	1.02–1.07	< 0.01*

^a^*IRR *Rate ratio for reported injury problem observations, ^b^*95% CI* 95% Confidence interval, ^c^*p-value* Probability value, ^d^*Ankle dorsiflexion* measured with Weight Bearing Dorsiflexion Lunge Test (WBLT) and calculated with a trigonometric dorsiflexion angle (TA), ^e^*AFAQ* Athlete fear avoidance questionnaire

*Denotes a statistically significant p-value at *p* < 0.05

**Table 7:** Factors associated with reported injury problems in the non-dominant leg based on multivariate analysis

Variables	Reported injury problems for the non-dominant leg		
	IRR^a^	95% CI^b^	*p*-value^c^
Age			
16–19	1		
20–24	1.04	0.70–1.53	0.86
> 24	1.21	0.81–1.83	0.36
AFAQ^d^			
(No fear/fear; 10–50)	1.04	1.02–1.06	< 0.01*

^a^*IRR *Rate ratio for reported injury problem observations, ^b^*95% CI* 95% Confidence interval, ^c^*p-value* Probability value, ^d^*AFAQ* Athlete fear avoidance questionnaire

*Denotes a statistically significant p-value at *p* < 0.05

#### Sensitivity analyses

To evaluate the adequacy and fit of the final model’s sensitivity analyses were conducted by identifying and excluding influential observations and re-estimating the models to assess robustness of the results. The sensitivity analyses showed that the direction of the associations (IRR) was generally stable following exclusion of influential observations. However, the precision (p-value) of the ankle dorsiflexion estimates was sensitive to the exclusion of some influential observations. For the model time-loss injury in the non-dominant leg, exclusion of two influential observations slightly altered the precision of the ankle dorsiflexion estimate so that it became non-significant, and for the model reported injury problems in the dominant leg, exclusion of one influential observation increased the precision of the ankle dorsiflexion estimate to be statistically significant.

The sensitivity analyses regarding the offset variable (exposure) did not change the IRRs and its precision (p-values) to a larger extent for both the dominant and non-dominant leg, except for the interaction effect for age (20–24 years) and hip strength, which became non-significant but didn’t change in the direction of association. Likewise, when totally excluding one third of the participants (*n* = 91) with incomplete data in the complete case analysis (CCA), some variables became non-significant but didn’t change in direction, see Additional file 6. All in all, the sensitivity analyses showed that the main findings were generally similar across the alternative analytical specifications, although some variation in the estimates was observed, especially for the CCA.

Finally, an additional sensitivity analysis was conducted in which lumbar-spine injury problem observations were excluded from the dominant- and non-dominant-leg models. Exclusion of lumbar-spine observations removed 182 and 185 observations from the dominant- and non-dominant-leg outcomes, respectively. The analyses showed that the main findings remained similar, as AFAQ was still significantly associated with a higher rate of reported injury problem observations in both the dominant (IRR = 1.05, 95% CI 1.03–1.08) and non-dominant leg (IRR = 1.05, 95% CI 1.02–1.07). Furthermore, ankle dorsiflexion remained non-significant in the dominant-leg model (IRR = 1.03, 95% CI 0.99–1.08). Thus, exclusion of lumbar-spine observations did not materially alter the main findings.

## Discussion

The main findings of the present study were that higher levels of hip strength were associated with lower time-loss injury incidence, and that higher AFAQ scores were associated with a higher rate of reported injury problem observations. These associations were observed in both the dominant and non-dominant leg. In addition, higher AFAQ scores were associated with higher time-loss injury incidence in the dominant leg.

### Hip strength in relation to injury

Our significant findings that a lower injury incidence of time-loss injuries associated with greater hip strength are consistent with previous research in senior female soccer player [30–33]. However, as a non-response concerning the OSTRC questionnaire for one month automatically assigned the participants 30 days of absence, further sensitivity analyses were performed regarding the offset variable (exposure) to assess the stability of the results. The sensitivity analyses compared four scenarios: (1) 30 days of absence per month, (2) 15 days of absence per month, (3) 0 days of absence per month, and (4) complete-case analysis, see Additional file 6. For time-loss injuries, hip strength was stable over the four scenarios for both legs except for the complete-case analysis in the dominant leg, where the findings got non-significant but didn’t change in direction (IRR = 0.97, *p* = 0.13). However, the interaction effect between age (20–24 years) and hip strength became non-significant in three of the four sensitivity-analysis scenarios for the dominant leg, although the direction and magnitude of the association remained largely unchanged across scenarios. Even though the results of the sensitivity analyses were generally similar across the alternative analytical specifications, the observed effect sizes were relatively small (IRR = 0.95–0.98) and should thus be considered when interpreting the results. Biomechanically, the hip abductors function as a knee stabiliser [30], and as the knee is among the most common injury locations in female soccer players [1, 2, 24], this represents one possible mechanism underlying an association between hip strength and injury. However, this mechanism was not investigated in the present study. Previous studies on hip strength, in both female athletes and non-athletes, confirm an association between weaker hip strength, patellar tendinopathy [66] and patellofemoral pain [67, 68]. On the contrary, a systematic review [69] on the relationship between hip muscle performance and injuries in the lower extremity provided limited evidence that hip muscle performance variables are related to such injuries. For the dominant leg, our findings showed an interaction between hip strength and age, indicating an attenuated association between hip strength and injury incidence in the two older age categories. In the age category 20–24 years, higher hip strength was not associated with injury incidence, while in the age category > 24 years, higher hip strength was significantly associated with a 4% lower injury incidence. In the dominant leg, hip strength was significantly lower than in the non-dominant leg (see Table 1). Whether this side-to-side difference could contribute to the different patterns of associations observed between the legs cannot be determined from the present study and requires further investigation. This side-to-side disparity in hip abduction strength was also discussed by Brophy et al. [31], who reported weaker hip abductor strength in the non-dominant leg compared with the dominant leg in senior female soccer players. They argued that this imbalance may contribute to the increased risk of an anterior cruciate ligament (ACL) injury, as well as other injuries, in female soccer players [31].

### Athlete fear avoidance in relation to injury

High levels of fear avoidance were significantly associated with a 4% higher rate of reported injury problem observations in both the dominant and non-dominant leg, and an 8% higher injury incidence for time-loss injuries in the dominant leg. Furthermore, the sensitivity analyses showed that the main findings were generally similar across the alternative analytical specifications, as the AFAQ estimates were generally stable across the four scenarios for time-loss injuries, although the association was no longer statistically significant in the complete-case analysis for injury problems in the non-dominant leg (IRR = 1.02, *p* = 0.09), see Additional file 6. Even if higher levels of fear avoidance were significantly associated with both time-loss injuries and injury problems, the small effect sizes limit the clinical significance of the results. Therefore, the results must be interpreted with caution in terms of clinical relevance and warrant further investigation to better understand the strength and consistency of these associations in clinical settings.

Psychosocial risk factors such as fear of avoidance, stress, anxiety, sleep quality, or coping mechanisms are proposed to be associated with an increased risk of both illness and musculoskeletal injuries among athletes in different sports [39, 70–72]. Specific research on senior female soccer players and psychosocial risk factors is sparse, but one study on time-loss injuries for male and female elite soccer players in the Swedish Premier League [41] and two studies on overuse injuries on both males and females in different sports (soccer included) [73, 74] indicate that psychosocial factors are of importance. In a study on male professional soccer players, Almansour et al. [75] reported that higher scores on the AFAQ were associated with poorer perceived functionality in the lower extremity. Furthermore, Paterno et al. [72] reported that patients with higher levels of fear of movement, measured with the Tampa Scale of Kinesiophobia, also related to an increased risk of suffering a second ACL injury after return to sport.

### Ankle dorsiflexion and division level in relation to injury

Concerning ankle dorsiflexion (ADF), higher ADF was significantly associated with a 9% lower injury incidence for time-loss injury in the non-dominant leg, but no statistically significant association was observed between ADF and reported injury problem observations in the dominant leg. However, the relatively low number of time-loss injuries may have limited the stability and precision of the TLI models, particularly those including interaction terms, and may have increased the influence of individual observations on some estimates. Previous research indicates an association between reduced ADF and an increased risk for knee injuries [40, 76–79] and other injuries in the lower extremity [80]. Even though our sensitivity analyses showed that the ADF estimates should be interpreted with caution, as they were sensitive to some influential observations, the different patterns observed between the dominant and non-dominant leg and between time-loss injuries and reported injury problem observations may warrant further investigation.

Playing in division 2 was significantly associated with a higher time-loss injury incidence in the non-dominant leg compared with division 1, while no statistically significant difference in injury incidence was observed between divisions 3 and 1. As the higher injury incidence observed in division 2 was not observed in division 3, the findings do not indicate a consistent pattern of increasing injury incidence with lower division level. However, clustering of players within teams was not accounted for in the analyses, and the observed association in division 2 may therefore partly reflect differences between individual teams rather than division level itself. Differences in injury reporting or other unmeasured contextual factors between divisions may also have contributed to this finding.

Even though research on amateur female soccer players is sparse and the comparison of different division levels might be complicated, research on injury incidence indicates that amateur players have a higher overall injury incidence compared to elite players [1, 6].

### Injury severity and leg dominance

In total, the present study registered 87 time-loss injuries and 1,397 reported injury problem observations in the dominant and non-dominant legs, distributed over 55,728 days of exposure. The season prevalence of time-loss injuries and cumulative season prevalence of reported injury problems were 27.1% and 84.4%, respectively. These results are difficult to compare with other studies due to methodological differences, i.e. how exposure and injury problems (including both minor and substantial injuries) were defined and quantified. However, the present study shows that reported injury problems were considerably more common than time-loss injuries, both in terms of the proportion of players affected during the season and the number of recorded observations. Similar to previous research, the most common injury location for time-loss injuries were the knee and foot [1, 2, 5]. On the other hand, for an injury problem the most common location was the foot and lumbar spine. Both the International Olympic Committee Consensus Statement [20] and the Oslo Sport Trauma Research Centre (OSTRC) [21, 46] propose that minor injuries/health problems could be filtered out from the more substantial injuries. However, we argue that playing with pain affects the athletes in the short and long term and that it could be a disadvantage to exclude minor injuries and pain in such an early stage of research. Especially as the OSTRC severity score is an arbitrary number, questionnaires are self-reported and subjective in nature, and the OSTRC severity score has not yet been validated as a proxy for injury severity [20, 21].

Another methodological approach in this study was to analyse the dominant and non-dominant legs separately, as leg dominance may be relevant to injury occurrence in soccer [35]. In accordance with previous studies, injury problems occurred most often in the dominant leg, but for time-loss injuries, there was no statistically significant difference between the legs. However, the two legs of the same player are not independent, and the separate leg-specific models did not formally account for the correlation between outcomes in the dominant and non-dominant leg. Consequently, differences in the patterns of associations observed between the separate leg-specific models should not be interpreted as evidence of statistically significant differences between legs.

### Clinical implications and future research

From a clinical perspective, the high frequency of reported injury problems highlights the importance of considering both time-loss injuries and less severe injury problems when monitoring player health. The associations observed with biomechanical and psychosocial factors support the relevance of a biopsychosocial perspective; however, the relatively small effect sizes, particularly for fear-avoidance behaviour, limit the clinical significance of the individual findings. Therefore, the factors identified in the present study should not be considered in isolation or interpreted as direct targets for injury prevention. Assessment of biomechanical and psychosocial characteristics may provide useful baseline information for clinicians, but their value for injury screening or prevention remains to be established. Future research should investigate whether these factors have practical relevance for injury prevention and whether changes in these factors are associated with subsequent changes in injury outcomes.

### Methodological considerations

The primary strengths of the current study include the participation of elite and sub-elite female soccer players, the recruitment of a substantial number of players, and the examination of both biomechanical and psychosocial factors. Furthermore, our analyses of the four models were based on valid and reliable instruments for collecting the data and in addition, robust statistical analysis. Nevertheless, we cannot rule out the risk that unmeasured or confounding factors might have affected the results. There are also some limitations that need to be considered. First, all injury data were self-reported and collected every fourth week, which might have introduced recall bias and affected data accuracy [81, 82]. To limit recall bias, the questions regarding injury problems referred to the previous seven days, as recommended by the OSTRC [21]. Consequently, injury problems occurring and resolving during the approximately three-week interval outside the seven-day reporting window may not have been captured, representing a limitation in the coverage of injury problems. Similarly, shorter or resolved time-loss injuries occurring during this approximately three-week interval may not have been captured, potentially resulting in an underestimation of the number of time-loss injuries. In addition, self-reported data without clinical verification may have resulted in some misclassification of injury status, and individual differences in the perception and reporting of symptoms may have influenced the injury data. Furthermore, the OSTRC severity score is an arbitrary numerical score and has not been validated as a proxy for injury severity [20, 21], which limits its interpretation as a measure of injury severity. Secondly, all physical tests were performed by a single examiner (JR), which ensured consistency in test administration but may have introduced systematic measurement error. No study-specific assessment of examiner reliability was performed. Thirdly, the rates of time-loss injuries and reported injury problem observations were based on days at risk, rather than on standard exposure measures such as injuries per 1000 h of training and match exposure. This way of calculating exposure has been used before to study injuries in college athletes [48, 49]. However, this limits comparison between our findings and the existing literature, as days at risk reflect “time under risk” rather than direct exposure to training or match play. Conceptually, “days at risk” can be viewed as a proxy for time under risk, which underlies traditional exposure-based measures. However, unlike exposure hours, this measure does not capture actual variation in the duration or intensity of risk exposure and should therefore be considered a less precise indicator of the underlying exposure. This approach may underestimate absolute rates compared with standard metrics based on exposure hours, as not all days include actual exposure to training or match play. On the other hand, relative measures such as rate ratios may be less sensitive to this limitation, provided that exposure patterns are similar across individuals. However, if actual exposure differs systematically between participants (e.g., training volume varies with psychological or physical characteristics), some degree of residual confounding may remain, meaning that the observed rate ratios may partly reflect differences in unmeasured football exposure rather than associations with the investigated factors alone. Therefore, as precise data on training and match exposure were not available in the present study, the results need to be interpreted with caution.

## Conclusion

Biomechanical factors, including hip strength, and psychosocial factors such as fear-avoidance behaviour were associated with time-loss injuries and reported injury problems in female elite and sub-elite soccer players. The separate leg-specific analyses showed different patterns of associations in the dominant and non-dominant leg, although it was not formally tested whether these associations differed statistically between legs. However, the relatively small effect sizes, particularly for fear-avoidance behaviour, limit conclusions regarding their clinical relevance. These findings should therefore be considered hypothesis-generating and warrant further research to determine whether biomechanical and psychosocial factors have clinically meaningful associations with injury outcomes.

## Supplementary Information

Additional file 1. Monthly injury registration-OSTRC.

Additional file 2. Code for statistical analysis in STATA 15.1.

Additional file 3. Univariate analyses of an injury problem in the dominant and non-dominant leg.

Additional file 4.Univariate analyses of a time-loss injury in the dominant and non-dominant leg.

Additional file 5. Linear combination of coefficients-Lincom.

Additional file 6. Sensitivity analyses regarding exposure.

Additional file 7. Guidelines for research documentation and data management at Karolinska Institutet.

## Data Availability

Data is not made open and available, since sensitive personal data could be traced to a living person, which contrasts with our regulations §6.3 in our Guidelines for research documentation and data management at Karolinska Institutet (DNR 1-20/2021), see Additional file 7. However, requests for data can be sent to the corresponding author and if there are no conflicts against the regulations, data could be made available in specific cases.

## Abbreviations

95% CI: 95% confidence interval
AFAQ: Athlete Fear Avoidance Questionnaire
ADF: Ankle dorsiflexion
ACL injury: Anterior cruciate ligament injury
AIC: Akaike´s Information Criterion
BIC: Bayesian Information Criterion
CCA: Complete Case Analysis
CLAM: Clamshell
DL: Dominant leg
FABQ: Fear Avoidance Beliefs Questionnaire
GAD-7: Generalized Anxiety Disorder-7 items
IIR: Injury Incidence Rate
IRR: Injury Rate Ratio
Lincom: Linear combinations of coefficients
N: Newton
Nm: Peak torque values
NDL: Non-dominant leg
OSTRC: Oslo Sports Trauma Research Centre
PCS: Pain Catastrophizing Scale
PSS-14: Perceived Stress Scale-14 items
PSQI: Pittsburgh Sleep Quality Index
SLS: Single Leg Squat
TSK: Tampa Scale
