# Supplementary material for: How are biomechanical and psychosocial factors associated with injuries across one season in Swedish elite and sub-elite female soccer players?
Source: BMC Sports Sci Med Rehabil. 2026 Sep 17;18:435. doi: 10.1186/s13102-026-02084-y (PMC13599122; doi:10.1186/s13102-026-02084-y)
Supplement: Supplementary file 1 [file 13102_2026_2084_MOESM1_ESM.pdf]

Additional file 1

Monthly Injury Registration 2022-01

Purpose, structure and time required for the questionnaire

The purpose of this questionnaire is to collect information about physical problems you may have experienced during the past 7 days and the past 4 weeks, respectively.

The questionnaire will be sent to you once a month until the season ends. It takes approximately 4 minutes to complete.

We use a body chart to describe the anatomical areas that we are interested in for this study. The body chart, with its five anatomical areas, is described below, and the questions refer only to these five areas. The questionnaire goes through each anatomical area in turn.

If you have not had any problems or injuries in these areas, simply click through the questions and indicate that **"you participate fully without problems"** (even if, for other reasons, you may not have participated fully).

If you currently have, or have had, any type of problem in one of the anatomical areas, you will be asked three follow-up questions about it before moving on to the next anatomical area.

The five anatomical areas are:

1. Back/buttock
2. Lower abdomen/groin/hip
3. Thigh
4. Knee
5. Lower leg/foot

Anatomical body chart, front and back view

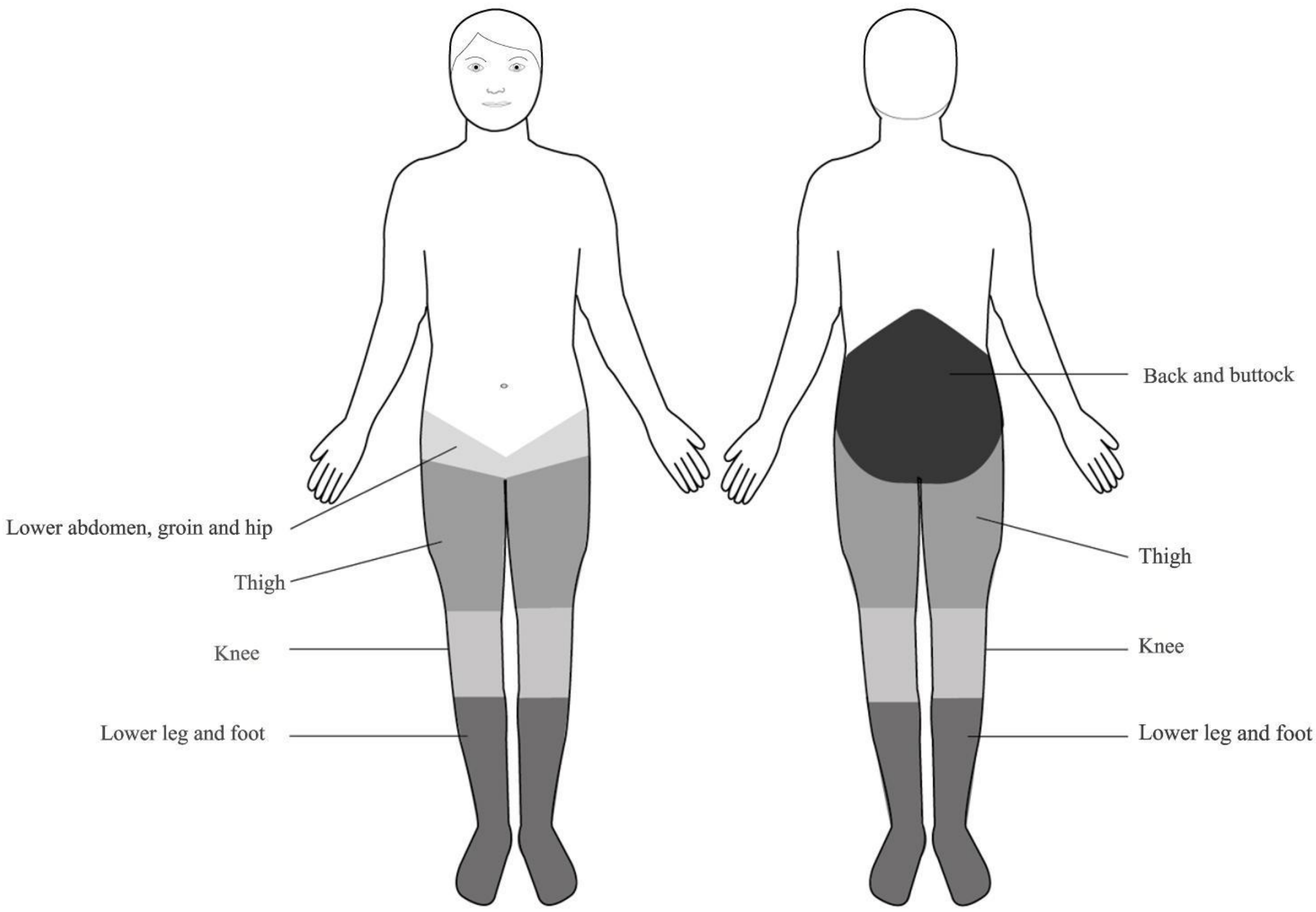

1. Problems from the back/buttock

Please answer all questions even if you do not have, or have not had, any problems with your back/buttock.

By back/buttock problems we mean pain, ache, stiffness, instability, swelling, locking, or other issues affecting the back/buttock.

Question 1A – Participation

Have you had problems participating in your sport (regular training/match/competition) due to your back/buttock problems during the past 7 days?

- Full participation, without back/buttock problems
- Full participation, but with back/buttock problems
- Reduced participation, due to back/buttock problems
- Could not participate, due to back/buttock problems

Question 1B – Modified training/competition

To what extent have you modified your training or competition due to back/buttock problems during the past 7 days?

- No modification
- To a minor extent
- To a moderate extent
- To a major extent

Question 1C – Performance

To what extent do you feel that your back/buttock problems have affected your sports performance during the past 7 days?

- No effect
- To a minor extent
- To a moderate extent
- To a major extent

Question 1D – Pain

To what extent have you experienced pain from your back/buttock during sports participation in the past 7 days?

- No pain
- Mild pain
- Moderate pain
- Severe pain

Days refraining from training/matches due to back/buttock problems in the past 4 weeks:

- 0 days
- 1–7 days
- 8–28 days
- More than 28 days

Side of the body affected:

- Right
- Left
- Centre
- Both sides
- Not applicable

2. Problems from the lower abdomen/groin/hip

Please answer all questions even if you do not have, or have not had, any problems with the lower abdomen/groin/hip.

By lower abdomen/groin/hip problems we mean pain, ache, stiffness, instability, swelling, locking, or other issues affecting the lower abdomen/groin/hip.

Question 2A – Participation

Have you had problems participating in your sport (regular training/match/competition) due to lower abdomen/groin/hip problems during the past 7 days?

- Full participation, without lower abdomen/groin/hip problems
- Full participation, but with lower abdomen/groin/hip problems
- Reduced participation, due to lower abdomen/groin/hip problems
- Could not participate, due to lower abdomen/groin/hip problems

Question 2B – Modified training/competition

To what extent have you modified your training or competition due to lower abdomen/groin/hip problems during the past 7 days?

- No modification
- To a minor extent
- To a moderate extent
- To a major extent

Question 2C – Performance

To what extent do you feel that your lower abdomen/groin/hip problems have affected your sports performance during the past 7 days?

- No effect
- To a minor extent
- To a moderate extent
- To a major extent

Question 2D – Pain

To what extent have you experienced pain from your lower abdomen/groin/hip during sports participation in the past 7 days?

- No pain
- Mild pain
- Moderate pain
- Severe pain

Days refraining from training/matches due to lower abdomen/groin/hip problems in the past 4 weeks:

- 0 days
- 1–7 days
- 8–28 days
- More than 28 days

Side of the body affected:

- Front
- Inner side
- Outer side
- Right
- Left
- Both sides
- Not applicable

3. Problems from the thigh

Please answer all questions even if you do not have, or have not had, any problems with the thigh.

By thigh problems we mean pain, ache, stiffness, instability, swelling, locking, or other issues affecting the thigh.

Question 3A – Participation

Have you had problems participating in your sport (regular training/match/competition) due to thigh problems during the past 7 days?

- Full participation, without thigh problems
- Full participation, but with thigh problems
- Reduced participation, due to thigh problems
- Could not participate, due to thigh problems

Question 3B – Modified training/competition

To what extent have you modified your training or competition due to thigh problems during the past 7 days?

- No modification
- To a minor extent
- To a moderate extent
- To a major extent

Question 3C – Performance

To what extent do you feel that your thigh problems have affected your sports performance during the past 7 days?

- No effect
- To a minor extent
- To a moderate extent
- To a major extent

Question 3D – Pain

To what extent have you experienced pain from your thigh during sports participation in the past 7 days?

- No pain
- Mild pain
- Moderate pain
- Severe pain

Days refraining from training/matches due to thigh problems in the past 4 weeks:

- 0 days
- 1–7 days
- 8–28 days
- More than 28 days

Location on the body affected:

- Front
- Inner side
- Back
- Right
- Left
- Both sides
- Not applicable

4. Problems from the knee

Please answer all questions even if you do not have, or have not had, any problems with the knee.

By knee problems we mean pain, ache, stiffness, instability, swelling, locking, or other issues affecting the knee.

Question 4A – Participation

Have you had problems participating in your sport (regular training/match/competition) due to knee problems during the past 7 days?

- Full participation, without knee problems
- Full participation, but with knee problems
- Reduced participation, due to knee problems
- Could not participate, due to knee problems

Question 4B – Modified training/competition

To what extent have you modified your training or competition due to knee problems during the past 7 days?

- No modification
- To a minor extent
- To a moderate extent
- To a major extent

Question 4C – Performance

To what extent do you feel that your knee problems have affected your sports performance during the past 7 days?

- No effect
- To a minor extent
- To a moderate extent
- To a major extent

Question 4D – Pain

To what extent have you experienced pain from your knee during sports participation in the past 7 days?

- No pain
- Mild pain
- Moderate pain
- Severe pain

Days refraining from training/matches due to knee problems in the past 4 weeks:

- 0 days
- 1–7 days
- 8–28 days
- More than 28 days

Location on the body affected:

- Front
- Back
- Outer side
- Inner side
- Right
- Left
- Both sides
- Not applicable

5. Problems from the lower leg/foot

Please answer all questions even if you do not have, or have not had, any problems with the lower leg/foot.

By lower leg/foot problems we mean pain, ache, stiffness, instability, swelling, locking, or other issues affecting the lower leg/foot.

Question 5A – Participation

Have you had problems participating in your sport (regular training/match/competition) due to lower leg/foot problems during the past 7 days?

- Full participation, without lower leg/foot problems
- Full participation, but with lower leg/foot problems
- Reduced participation, due to lower leg/foot problems
- Could not participate, due to lower leg/foot problems

Question 5B – Modified training/competition

To what extent have you modified your training or competition due to lower leg/foot problems during the past 7 days?

- No modification
- To a minor extent
- To a moderate extent
- To a major extent

Question 5C – Performance

To what extent do you feel that your lower leg/foot problems have affected your sports performance during the past 7 days?

- No effect
- To a minor extent
- To a moderate extent
- To a major extent

Question 5D – Pain

To what extent have you experienced pain from your lower leg/foot during sports participation in the past 7 days?

- No pain
- Mild pain
- Moderate pain
- Severe pain

Days refraining from training/matches due to lower leg/foot problems in the past 4 weeks:

- 0 days
- 1–7 days
- 8–28 days
- More than 28 days

Location on the body affected:

- Front
- Back
- Outer side
- Inner side
- Underside
- Right
- Left
- Both sides
- Not applicable

Other reasons for refraining from training/matches

We are interested in whether there may have been other reasons (apart from physical problems in the five anatomical areas) that have caused you to refrain from training/matches during the past 4 weeks. For example, due to illness, holiday, school/work, family, etc.

Please indicate below how many days during the past 4 weeks you have had to refrain from training/matches due to other reasons (e.g., holiday, illness, school/work, family) **not related to physical problems in the five anatomical areas:**

- 0 days
- 1–7 days
- 1–2 weeks
- 2–3 weeks
