# Supplementary material for: How are biomechanical and psychosocial factors associated with injuries across one season in Swedish elite and sub-elite female soccer players?
Source: BMC Sports Sci Med Rehabil. 2026 Sep 17;18:435. doi: 10.1186/s13102-026-02084-y (PMC13599122; doi:10.1186/s13102-026-02084-y)
Supplement: Supplementary file 2 [file 13102_2026_2084_MOESM2_ESM.pdf]

Additional file 2

Code for statistical analysis in STATA 15.1

*Table 1: Subject characteristics stratified by division.

```
tab div

sum Age, detail
sum Age if div==1, detail
sum Age if div==2, detail
sum Age if div==3, detail

sum Lengthm, detail
sum Lengthm if div==1, detail
sum Lengthm if div==2, detail
sum Lengthm if div==3, detail

sum Weightkg, detail
sum Weightkg if div==1, detail
sum Weightkg if div==2, detail
sum Weightkg if div==3, detail

sum NDL_ADF, detail
sum NDL_ADF if div==1, detail
sum NDL_ADF if div==2, detail
sum NDL_ADF if div==3, detail
sum DL_ADF, detail
sum DL_ADF if div==1, detail
sum DL_ADF if div==2, detail
sum DL_ADF if div==3, detail
signrank NDL_ADF= DL_ADF //p= 0.0387
```

ttest DL_ADF=NDL_ADF //p=0.0484, ADF is normally distributed and a paired t-test could be used

sum DL_Nm3BWm_100, detail

sum DL_Nm3BWm_100 if div==1, detail

sum DL_Nm3BWm_100 if div==2, detail

sum DL_Nm3BWm_100 if div==3, detail

sum NDL_Nm3BWm_100, detail

sum NDL_Nm3BWm_100 if div==1, detail

sum NDL_Nm3BWm_100 if div==2, detail

sum NDL_Nm3BWm_100 if div==3, detail

signrank NDL_Nm3BWm_100=DL_Nm3BWm_100 //p=0.043 (Wilcoxon signed-rank test Is a paired test for non-normally distributed data)

sum AFAQ, detail

sum AFAQ if div==1, detail

sum AFAQ if div==2, detail

sum AFAQ if div==3, detail

sum PSS14, detail

sum PSS14 if div==1, detail

sum PSS14 if div==2, detail

sum PSS14 if div==3, detail

sum PSQI, detail

sum PSQI if div==1, detail

sum PSQI if div==2, detail

sum PSQI if div==3, detail

sum GAD7, detail

sum GAD7 if div==1, detail

sum GAD7 if div==2, detail

sum GAD7 if div==3, detail

***Table 2. Injury location and frequency for time-loss injuries and injury problems in the dominant and non-dominant leg in female soccer players over a 10-month season.**

```
// Injury location for TLI//
tab TLI_DL_Länd
tab TLI_DL_Ljumske
tab TLI_DL_Lår
tab TLI_DL_Knä
tab TLI_DL_Fot
di 1+7+3+15+11
di 1/37
di 7/37
di 3/37
di 15/37
di 11/37

tab TLI_NDL_Länd
tab TLI_NDL_Ljumske
tab TLI_NDL_Lår
tab TLI_NDL_Knä
tab TLI_NDL_Fot
di 1+8+4+19+18
di 1/50
di 8/50
di 4/50
di 19/50
di 18/50
di 2+16+8+38+36
di 1+8+4+18+15

di 182/736
signrank TLI_Alla_Segment_NDL =TLI_Alla_Segment_DL //p=0.2776
```

//Injury location for IP (OSTRC)

tab Total_OSTRC_DL_Länd_Counts

tab Total_OSTRC_DL_Ljumske_Counts

tab Total_OSTRC_DL_Lår_Counts

tab Total_OSTRC_DL_Knä_Counts

tab Total_OSTRC_DL_Fot_Counts

tab Total_OSTRC_NDL_Länd_Counts

tab Total_OSTRC_NDL_Ljumske_Counts

tab Total_OSTRC_NDL_Lår_Counts

tab Total_OSTRC_NDL_Knä_Counts

tab Total_OSTRC_NDL_Fot_Counts //further calculations are done in an excel sheet named "statisktik table 3"

ttest Total_OSTRC_Alla_Segm_NDL_Counts== Total_OSTRC_Alla_Segm_DL_Counts //p=0.0803

signrank Total_OSTRC_Alla_Segm_NDL_Counts=Total_OSTRC_Alla_Segm_DL_Counts //p=0.0175

*Table 3. Descriptive statistics for time-loss injuries and injury problems over a 10-month season.

//Exposure days, per player season

total Exposure_Days_Md // 55 728 days, Std.err. 1269.241, CI=53227.88-58228.12

sum Exposure_Days_Md // Observations=244, Mean=228.3934, Std.dev=81.25483, Min=30, Max=300

//Time-loss injuries, per player season

sum TLI_Alla_Segment_DL // Obsesrvations= 244, Mean=0.1516393, Std.dev=0.4026116, Min=0, Max=2

total TLI_Alla_Segment_DL // total=37, Std.err=6.288994, CI=24.6121-49.3879

sum TLI_Alla_Segment_NDL //Observations=244, Mean=0.204918, Std.dev=0.4875153, Min=0, Max=3

total TLI_Alla_Segment_NDL //total=50, Std.err=7.615233, CI=34.99971-65.00029

```
gen TLI_total = TLI_Alla_Segment_DL + TLI_Alla_Segment_NDL
sum TLI_total //observations=244, Mean=0.3565574, Std dev=0.6604011, Min=0, Max=3
total TLI_total //total=87, Std.err.=10.3158, CI=66.68021-107.3198

//Time-loss injury incidence, per 1000 days exposure
//If you want to use scalar function:
summarize TLI_Alla_Segment_NDL
scalar antal_TLI_NDL= r(sum)
summarize Exposure_Days_Md
scalar total_exponering_Md= r(sum)
display "Injury Incidence Rate per 1000 dagar: " //(antal_TLI_NDL / total_exponering_Md) *
1000

//Injury Incidence Rate per 1000 days for the NDL= .89721504

total TLI_Alla_Segment_NDL // 50
total Exposure_Days_Md // 55728
di (50/55728)*1000 //Injury Incidence Rate per 1000 days for the NDL= .89721504
local x = 50
local t = 55728 //Saving my values to estimate the CI
local lower = `=invchi2(2*`x', 0.025) / 2'
local upper = `=invchi2(2*(`x'+1), 0.975) / 2' // CI poisson based
display "IIR = " `=(`x'/`t')*1000'
display "Lower 95% CI = " `=(`lower'/`t')*1000'
display "Upper 95% CI = " `=(`upper'/`t')*1000'  //recalculating to injuries per 1000 days of
exposure. Lower 95% CI = .6659303, Upper 95% CI = 1.1828662


total TLI_Alla_Segment_DL // 37
total Exposure_Days_Md // 55728
di (37/55728)*1000 // Injury Incidence Rate per 1000 days for the DL= .66393913
local x = 37
```

```
local t = 55728

local lower = `=invchi2(2*`x', 0.025) / 2'

local upper = `=invchi2(2*(`x'+1), 0.975) / 2'

display "IIR = " `=(`x'/`t')*1000'

display "Lower 95% CI = " `=(`lower'/`t')*1000'

display "Upper 95% CI = " `=(`upper'/`t')*1000' //Lower 95% CI = .46747442, Upper 95% CI = .91515264



total TLI_total //87 Total TLI

total Exposure_Days_Md //55728 Total exposure

di (87/55728)*1000 // Injury Incidence Rate per 1000 days for both legs= 1.5611542

local x = 87

local t = 55728

scalar lower = invchi2(2*`x', 0.025) / 2

scalar upper = invchi2(2*(`x'+1), 0.975) / 2

display "IIR = " %6.3f (`x'/`t')*1000  //IIR =  1.561

display "Lower 95% CI = " %6.3f (lower/`t')*1000 //Lower 95% CI =  1.250

display "Upper 95% CI = " %6.3f (upper/`t')*1000 //Upper 95% CI =  1.926


//Time-loss injury prevalence, 10-month (number of injured players/total number of players *100)


gen haft_skada_NDL = TLI_Alla_Segment_NDL > 0

tabulate haft_skada_NDL

summarize haft_skada_NDL

display "Prevalens (%): " r(mean) * 100 // Prevalens (%): 17.213115

ci means haft_skada_NDL // CI=0.1244305-0.2198317

proportion haft_skada_NDL //CI=0.1295833-0.2250374  (Använd detta CI)


gen haft_skada_DL = TLI_Alla_Segment_DL > 0

tabulate haft_skada_DL

summarize haft_skada_DL
```

display "Prevalens (%): " r(mean) * 100 //Prevalens (%): 13.52459

ci means haft_skada_DL //CI=0.0920322-0.1784596

proportion haft_skada_DL //CI=0.0976084-0.1844301 (Använd detta CI)

gen haft_skada_totalt = (TLI_Alla_Segment_DL > 0) | (TLI_Alla_Segment_NDL > 0)

tabulate haft_skada_totalt

summarize haft_skada_totalt

display "Total skadeprevalens (%): " r(mean) * 100 // Total skadeprevalens (%): 27.04918

ci means haft_skada_totalt // CI=0.2143605-0.3266232

proportion haft_skada_totalt // CI=0.218231-0.3299851 (Använd detta CI)

//Injury problem, per player season

sum Total_OSTRC_Alla_Segm_DL_Counts //Obserations=244, Mean=3.016393, Std dev=3.56938, Min=0, Max=22

total Total_OSTRC_Alla_Segm_DL_Counts //total=736, Std.err=55.75549, CI=626.1743-845.8257

sum Total_OSTRC_Alla_Segm_NDL_Counts //Observations=244, Mean=2.709016, Std dev=3.253577, Min=0, Max=16

total Total_OSTRC_Alla_Segm_NDL_Counts // Total=661, Std err=50.82249, CI=560.8912-761.1088

gen OSTRC_Total_Counts=Total_OSTRC_Alla_Segm_DL_Counts + Total_OSTRC_Alla_Segm_NDL_Counts

sum OSTRC_Total_Counts // Observations=244, Mean=5.72541, Std dev=6.259348, Min=0, Max=37

total OSTRC_Total_Counts //Total=1397, Std Err=97.77414, CI=1204.407-1589.593

//Injury problem incidence, per 1000 days exposure

total Total_OSTRC_Alla_Segm_DL_Counts

total Exposure_Days_Md

di (736/55728)*1000 //Injury problem Incidence Rate per 1000 days for the DL= 13.207005

```
local x = 736

local t = 55728 //Saving my values to estimate the CI

local lower = `=invchi2(2*`x', 0.025) / 2'

local upper = `=invchi2(2*(`x'+1), 0.975) / 2' // CI poisson based

display "IIR = " `=(`x'/`t')*1000'

display "Lower 95% CI = " `=(`lower'/`t')*1000'

display "Upper 95% CI = " `=(`upper'/`t')*1000' //Lower 95% CI = 12.26997, Upper 95% CI = 14.196622


total Total_OSTRC_Alla_Segm_NDL_Counts

total Exposure_Days_Md

di (661/55728)*1000 //Injury problem Incidence Rate per 1000 days for the NDL= 11.861183

local x = 661

local t = 55728 //Saving my values to estimate the CI

local lower = `=invchi2(2*`x', 0.025) / 2'

local upper = `=invchi2(2*(`x'+1), 0.975) / 2' // CI poisson based

display "IIR = " `=(`x'/`t')*1000'

display "Lower 95% CI = " `=(`lower'/`t')*1000'

display "Upper 95% CI = " `=(`upper'/`t')*1000' //Lower 95% CI = 10.974074, Upper 95% CI = 12.800908


total OSTRC_Total_Counts

total Exposure_Days_Md

di (1397/55728)*1000 //Injury problem Incidence Rate per 1000 days for both legs= 25.068188

local x = 1397

local t = 55728 //Saving my values to estimate the CI

local lower = `=invchi2(2*`x', 0.025) / 2'

local upper = `=invchi2(2*(`x'+1), 0.975) / 2' // CI poisson based

display "IIR = " `=(`x'/`t')*1000'

display "Lower 95% CI = " `=(`lower'/`t')*1000'

display "Upper 95% CI = " `=(`upper'/`t')*1000' //Lower 95% CI = 23.770729, Upper 95% CI = 26.418053
```

```
//Injury problem prevalence, 10-month (number of injured players/total number of players *100)

gen haft_OSTRC_NDL= Total_OSTRC_Alla_Segm_NDL_Counts >0

tab haft_OSTRC_NDL

summarize haft_OSTRC_NDL

display "Prevalens (%): " r(mean) * 100 // Prevalens (%): 74.180328

ci means haft_OSTRC_NDL // CI=0.6865023-0.7971043

proportion haft_OSTRC_NDL //CI=0.68292-0.7930651 (Använd detta CI)


gen haft_OSTRC_DL= Total_OSTRC_Alla_Segm_DL_Counts >0

tab haft_OSTRC_DL

summarize haft_OSTRC_DL

display "Prevalens (%): " r(mean) * 100 // Prevalens (%): 73.770492

ci means haft_OSTRC_DL // CI=0.6821209-0.7932889

proportion haft_OSTRC_DL //CI=0.6786123-0.7893057(Använd detta CI)


gen haft_OSTRC_Total= (Total_OSTRC_Alla_Segm_NDL_Counts >0) | (Total_OSTRC_Alla_Segm_DL_Counts >0)

tabulate haft_OSTRC_Total

summarize haft_OSTRC_Total

display "Total skadeprevalens (%): " r(mean) * 100 // Total skadeprevalens (%): 84.42623

ci means haft_OSTRC_Total // CI=0.7984429-0.8900816

proportion haft_OSTRC_Total // CI=0.7929049-0.8847351(Använd detta CI)
```

*MODEL BUILDING TIME-LOSS INJURIES: CONTROL OF ASSUMPTIONS FOR BOTH NDL AND DL

* STEP 1. DESKRIPTIV ANALYSIS

//Evaluating the covariates for outliers, missing data (most of this is done in study 4 for those variables so this is mostly a quick check) and its relation to the

//outcome variable

//The variable div

poisson TLI_Alla_Segment_NDL div, exposure (Exposure_Days_Md) irr

poisson TLI_Alla_Segment_NDL i.div, exposure (Exposure_Days_Md) irr //***p=0.02

tab div

tab TLI_Alla_Segment_NDL div //relatively few subjects with 2 or more injuries in all divisions.

poisson TLI_Alla_Segment_DL div, exposure (Exposure_Days_Md) irr

poisson TLI_Alla_Segment_DL i.div, exposure (Exposure_Days_Md) irr

tab div

tab TLI_Alla_Segment_DL div //relatively few subjects with 2 or more injuries in all divisions.

//The variable Age

poisson TLI_Alla_Segment_NDL Age, exposure (Exposure_Days_Md) irr //p=0.763

sum Age, detail

graph box Age //Two outliers

scatter Age Subject, mlabel(Subject) //Subjects 76 and 143 is the outliers

poisson TLI_Alla_Segment_NDL Age, exposure (Exposure_Days_Md) irr

poisson TLI_Alla_Segment_NDL Age if Subject!=143 & Subject !=76, exposure (Exposure_Days_Md) irr // Conclusion: No significant change in IRR, CI, Std error or P

poisson TLI_Alla_Segment_DL Age, exposure (Exposure_Days_Md) irr //p=0637

sum Age, detail

graph box Age //Two outliers

scatter Age Subject, mlabel(Subject) //Subjects 76 and 143 is the outliers

poisson TLI_Alla_Segment_DL Age, exposure (Exposure_Days_Md) irr

poisson TLI_Alla_Segment_DL Age if Subject!=143 & Subject !=76, exposure (Exposure_Days_Md) irr // Conclusion: No significant change in IRR, CI, Std error or P

//The variable Weightkg

poisson TLI_Alla_Segment_NDL Weightkg, exposure (Exposure_Days_Md) irr //p=0.168

sum Weightkg, detail

graph box Weightkg //three outliers

scatter Weightkg Subject, mlabel (Subject) //subjects 34, 195 and 231 are outliers

poisson TLI_Alla_Segment_NDL Weightkg, exposure (Exposure_Days_Md) irr

poisson TLI_Alla_Segment_NDL Weightkg if Subject!=34 & Subject !=195 & Subject !=231, exposure (Exposure_Days_Md) irr

// Conclusion: No significant change in IRR, CI, Std error or P

poisson TLI_Alla_Segment_DL Weightkg, exposure (Exposure_Days_Md) irr // p=0.90

sum Weightkg, detail

graph box Weightkg //three outliers

scatter Weightkg Subject, mlabel (Subject) //subjects 34, 195 and 231 are outliers

poisson TLI_Alla_Segment_DL Weightkg, exposure (Exposure_Days_Md) irr

poisson TLI_Alla_Segment_DL Weightkg if Subject!=34 & Subject !=195 & Subject !=231, exposure (Exposure_Days_Md) irr

// Conclusion: No significant change in P value but the IRR goes from 0.99 to 1.005

//The variable Lengthm

poisson TLI_Alla_Segment_NDL Lengthm, exposure (Exposure_Days_Md) irr //p=0.30

sum Lengthm, detail

graph box Lengthm //One outlier

scatter Lengthm Subject, mlabel (Subject) //subject 88 is an outlier

poisson TLI_Alla_Segment_NDL Lengthm, exposure (Exposure_Days_Md) irr

poisson TLI_Alla_Segment_NDL Lengthm if Subject!=88, exposure (Exposure_Days_Md) irr

// Conclusion: No significant change in P value but the IRR goes from 16 to 13

poisson TLI_Alla_Segment_DL Lengthm, exposure (Exposure_Days_Md) irr //p=0.40

sum Lengthm, detail

graph box Lengthm //One outlier

scatter Lengthm Subject, mlabel (Subject) //subject 88 is an outlier

poisson TLI_Alla_Segment_DL Lengthm, exposure (Exposure_Days_Md) irr

poisson TLI_Alla_Segment_DL Lengthm if Subject!=88, exposure (Exposure_Days_Md) irr // Conclusion: No significant change in IRR, CI, Std error or P

//The variable BMI

poisson TLI_Alla_Segment_NDL BMI, exposure (Exposure_Days_Md) irr //**p=0.38

sum BMI, detail

graph box BMI

scatter BMI Subject, mlabel (Subject) //Subject 34 is an outlier

list Subject BMI

poisson TLI_Alla_Segment_NDL BMI if Subject !=34, exposure (Exposure_Days_Md) irr // Conclusion: No significant change in OR, CI, Std error or P

poisson TLI_Alla_Segment_DL BMI, exposure (Exposure_Days_Md) irr //**p=0.58

sum BMI, detail

graph box BMI

scatter BMI Subject, mlabel (Subject) //Subject 34 is an outlier

list Subject BMI

poisson TLI_Alla_Segment_DL BMI if Subject !=34, exposure (Exposure_Days_Md) irr // Conclusion: No significant change in OR, CI, Std error or P

//The variable SLS/passfail

poisson TLI_Alla_Segment_NDL NDL_SLSfailpass, exposure (Exposure_Days_Md) irr //**p=0.86

tab NDL_SLSfailpass

tab NDL_SLSfailpass TLI_Alla_Segment_NDL

```
poisson TLI_Alla_Segment_DL DL_SLSfailpass, exposure ( Exposure_Days_Md ) irr
//**p=0.26

tab DL_SLSfailpass

tab DL_SLSfailpass TLI_Alla_Segment_DL


//The variable SLStotal and

poisson TLI_Alla_Segment_NDL NDL_SLStotal_4cat, exposure ( Exposure_Days_Md ) irr
//**p=0.50

tab NDL_SLStotal_4cat

tab NDL_SLStotal_4cat TLI_Alla_Segment_NDL


poisson TLI_Alla_Segment_DL DL_SLStotal, exposure ( Exposure_Days_Md ) irr
//**p=0.09

tab DL_SLStotal

tab DL_SLStotal TLI_Alla_Segment_DL


//The variable ADF

poisson TLI_Alla_Segment_NDL NDL_ADF, exposure ( Exposure_Days_Md ) irr
//**p=0.03

sum NDL_ADF, detail

graph box NDL_ADF //Two outliers

scatter NDL_ADF Subject, mlabel (Subject) //Subject 109 and 251 is the outliers

list Subject NDL_ADF

poisson TLI_Alla_Segment_NDL NDL_ADF if Subject !=109 & Subject !=251, exposure (
Exposure_Days_Md ) irr //a small change with better P value and IRR


poisson TLI_Alla_Segment_DL DL_ADF, exposure ( Exposure_Days_Md ) irr //**p=0.73

sum DL_ADF, detail

graph box DL_ADF //Three outliers

scatter DL_ADF Subject, mlabel (Subject) //Subject 109, 258 and 6 is the outliers

list Subject DL_ADF

poisson TLI_Alla_Segment_DL DL_ADF if Subject !=109 & Subject !=258 & Subject !=6,
exposure ( Exposure_Days_Md ) irr //A change but not significant
```

//The variable CLAM

poisson TLI_Alla_Segment_NDL NDL_Nm3BWm_100, exposure (Exposure_Days_Md) irr //**p=0.013

sum NDL_Nm3BWm_100, detail

graph box NDL_Nm3BWm_100 //Three outliers

scatter NDL_Nm3BWm_100 Subject, mlabel (Subject) //Subject 261, 90, 41 is the outliers

list Subject NDL_Nm3BWm_100

poisson TLI_Alla_Segment_NDL NDL_Nm3BWm_100 if Subject !=261 & Subject !=90 & Subject !=41, exposure (Exposure_Days_Md) irr //No significant change in OR, CI, Std error or P

poisson TLI_Alla_Segment_DL DL_Nm3BWm_100, exposure (Exposure_Days_Md) irr //**p=0.002

sum DL_Nm3BWm_100, detail

graph box DL_Nm3BWm_100 //four outliers

scatter DL_Nm3BWm_100 Subject, mlabel (Subject) //Subject 261, 245, 226 and 90 is the outliers

list Subject DL_Nm3BWm_100

poisson TLI_Alla_Segment_DL DL_Nm3BWm_100 if Subject !=261 & Subject !=245 & Subject !=226 & Subject !=90, exposure (Exposure_Days_Md) irr //No significant change in OR, CI, Std error or P

//The variable AFAQ

poisson TLI_Alla_Segment_NDL AFAQ, exposure (Exposure_Days_Md) irr //**p=0.14

sum AFAQ, detail

graph box AFAQ //Three outliers

scatter AFAQ Subject, mlabel (Subject) //Subject 80 and 55 and 43 is the outliers

list Subject AFAQ

poisson TLI_Alla_Segment_NDL AFAQ if Subject !=80 & Subject !=55 & Subject !=43, exposure (Exposure_Days_Md) irr //A worse p value

poisson TLI_Alla_Segment_DL AFAQ, exposure (Exposure_Days_Md) irr //**p=0.003

sum AFAQ, detail

graph box AFAQ //three outliers

scatter AFAQ Subject, mlabel (Subject) //Subject 80 and 55 and 43 is the outliers

```
list Subject AFAQ

poisson TLI_Alla_Segment_DL AFAQ if Subject !=80 & Subject !=55 & Subject !=43, exposure ( Exposure_Days_Md ) irr // Better P value and IRR


//The variable PSS14

poisson TLI_Alla_Segment_NDL PSS14, exposure ( Exposure_Days_Md ) irr //**p=0.97

sum PSS14, detail

graph box PSS14 //Tw0 outliers

scatter PSS14 Subject, mlabel (Subject) //Subject 46 and 189 is the outliers

list Subject PSS14

poisson TLI_Alla_Segment_NDL PSS14 if Subject !=46 & Subject !=189, exposure ( Exposure_Days_Md ) irr //No change


poisson TLI_Alla_Segment_DL PSS14, exposure ( Exposure_Days_Md ) irr //**p=0.338

sum PSS14, detail

graph box PSS14 //three outliers

scatter PSS14 Subject, mlabel (Subject) //Subject 46 and 189 is the outliers

list Subject PSS14

poisson TLI_Alla_Segment_DL PSS14 if Subject !=46 & Subject !=189, exposure ( Exposure_Days_Md ) irr //no change


//The variable PSQI (was categorized in study 4 and will be used that way in this study too)

poisson TLI_Alla_Segment_NDL PSQI_cat2, exposure ( Exposure_Days_Md ) irr //**p=0.89

sum PSQI, detail

graph box PSQI //Two outliers

scatter PSQI Subject, mlabel (Subject) //Subject 225 and 92 is the outliers

list Subject PSQI

poisson TLI_Alla_Segment_NDL PSQI_cat2 if Subject !=92 & Subject !=225, exposure ( Exposure_Days_Md ) irr //No change


poisson TLI_Alla_Segment_DL PSS14, exposure ( Exposure_Days_Md ) irr //**p=0.338

sum PSS14, detail

graph box PSS14 //two outliers
```

scatter PSS14 Subject, mlabel (Subject) //Subject 225 and 92 is the outliers

list Subject PSS14

poisson TLI_Alla_Segment_DL PSS14 if Subject !=92 & Subject !=225, exposure (Exposure_Days_Md) irr //no change

//The variable GAD-7 (was categorized in study 4 and will be used that way in this study too)

poisson TLI_Alla_Segment_NDL GAD7, exposure (Exposure_Days_Md) irr //**p=0.055

sum GAD7, detail

graph box GAD7 //four outliers

scatter GAD7 Subject, mlabel (Subject) //Subjects 45, 31, 7 and 230 are the outliers

list Subject GAD7

poisson TLI_Alla_Segment_NDL GAD7 if Subject !=7 & Subject !=31 & Subject !=45 & Subject !=230, exposure (Exposure_Days_Md) irr //a better p value

poisson TLI_Alla_Segment_DL GAD7, exposure (Exposure_Days_Md) irr //**p=0.11

sum GAD7, detail

graph box GAD7//four outliers

scatter GAD7 Subject, mlabel (Subject) //Subjects 45, 31, 7 and 230 are the outliers

list Subject GAD7

poisson TLI_Alla_Segment_DL GAD7 if Subject !=7 & Subject !=31 & Subject !=45 & Subject !=230, exposure (Exposure_Days_Md) irr //better p value and irr

//OVERALL CONCLUSION: no greater outliers due to wrongly registered data or missing data/////////////////////////////

* STEP 2 EVALUATING THE OUTCOME VARAIALBE FOR ZERO INFLATION AND THE OFFSET VARIABLE

```
//Evaluating the outcome variable and exposure (offset variable).

//A.Exposure
sum Exposure_Days_Min, detail //mean 237, max 300, min 30
sum Exposure_Days_Md, detail //mean 228, max 300, min 30
sum Exposure_Days_Max, detail //mean 219, max 300, min 27

poisson TLI_Alla_Segment_NDL BMI, exposure (Exposure_Days_Min) irr
poisson TLI_Alla_Segment_NDL BMI, exposure (Exposure_Days_Md) irr
poisson TLI_Alla_Segment_NDL BMI, exposure (Exposure_Days_Max) irr

poisson TLI_Alla_Segment_NDL Age, exposure (Exposure_Days_Min) irr
poisson TLI_Alla_Segment_NDL Age, exposure (Exposure_Days_Md) irr
poisson TLI_Alla_Segment_NDL Age, exposure (Exposure_Days_Max) irr

poisson TLI_Alla_Segment_NDL NDL_SLStotal_4cat, exposure (Exposure_Days_Min) irr
poisson TLI_Alla_Segment_NDL NDL_SLStotal_4cat, exposure (Exposure_Days_Md) irr
poisson TLI_Alla_Segment_NDL NDL_SLStotal_4cat, exposure (Exposure_Days_Max) irr

poisson TLI_Alla_Segment_NDL NDL_SLSfailpass, exposure (Exposure_Days_Min) irr
poisson TLI_Alla_Segment_NDL NDL_SLSfailpass, exposure (Exposure_Days_Md) irr
poisson TLI_Alla_Segment_NDL NDL_SLSfailpass, exposure (Exposure_Days_Max) irr

poisson TLI_Alla_Segment_NDL NDL_ADF, exposure (Exposure_Days_Min) irr
poisson TLI_Alla_Segment_NDL NDL_ADF, exposure (Exposure_Days_Md) irr
poisson TLI_Alla_Segment_NDL NDL_ADF, exposure (Exposure_Days_Max) irr

poisson TLI_Alla_Segment_NDL NDL_Nm3BWm_100, exposure (Exposure_Days_Min) irr
poisson TLI_Alla_Segment_NDL NDL_Nm3BWm_100, exposure (Exposure_Days_Md) irr
poisson TLI_Alla_Segment_NDL NDL_Nm3BWm_100, exposure (Exposure_Days_Max) irr
```

```
poisson TLI_Alla_Segment_NDL AFAQ, exposure (Exposure_Days_Min) irr
poisson TLI_Alla_Segment_NDL AFAQ, exposure (Exposure_Days_Md) irr
poisson TLI_Alla_Segment_NDL AFAQ, exposure (Exposure_Days_Max) irr

poisson TLI_Alla_Segment_NDL PSS14, exposure (Exposure_Days_Min) irr
poisson TLI_Alla_Segment_NDL PSS14, exposure (Exposure_Days_Md) irr
poisson TLI_Alla_Segment_NDL PSS14, exposure (Exposure_Days_Max) irr

poisson TLI_Alla_Segment_NDL PSQI, exposure (Exposure_Days_Min) irr
poisson TLI_Alla_Segment_NDL PSQI, exposure (Exposure_Days_Md) irr
poisson TLI_Alla_Segment_NDL PSQI, exposure (Exposure_Days_Max) irr

poisson TLI_Alla_Segment_NDL GAD7, exposure (Exposure_Days_Min) irr
poisson TLI_Alla_Segment_NDL GAD7, exposure (Exposure_Days_Md) irr
poisson TLI_Alla_Segment_NDL GAD7, exposure (Exposure_Days_Max) irr


poisson TLI_Alla_Segment_DL BMI, exposure (Exposure_Days_Min) irr
poisson TLI_Alla_Segment_DL BMI, exposure (Exposure_Days_Md) irr
poisson TLI_Alla_Segment_DL BMI, exposure (Exposure_Days_Max) irr

poisson TLI_Alla_Segment_DL Age, exposure (Exposure_Days_Min) irr
poisson TLI_Alla_Segment_DL Age, exposure (Exposure_Days_Md) irr
poisson TLI_Alla_Segment_DL Age, exposure (Exposure_Days_Max) irr

poisson TLI_Alla_Segment_DL DL_SLStotal, exposure (Exposure_Days_Min) irr
poisson TLI_Alla_Segment_DL DL_SLStotal, exposure (Exposure_Days_Md) irr
poisson TLI_Alla_Segment_DL DL_SLStotal, exposure (Exposure_Days_Max) irr

poisson TLI_Alla_Segment_DL DL_SLSfailpass, exposure (Exposure_Days_Min) irr
poisson TLI_Alla_Segment_DL DL_SLSfailpass, exposure (Exposure_Days_Md) irr
```

```
poisson TLI_Alla_Segment_DL DL_SLSfailpass, exposure (Exposure_Days_Max) irr

poisson TLI_Alla_Segment_DL DL_ADF, exposure (Exposure_Days_Min) irr
poisson TLI_Alla_Segment_DL DL_ADF, exposure (Exposure_Days_Md) irr
poisson TLI_Alla_Segment_DL DL_ADF, exposure (Exposure_Days_Max) irr

poisson TLI_Alla_Segment_DL DL_Nm3BWm_100, exposure (Exposure_Days_Min) irr
poisson TLI_Alla_Segment_DL DL_Nm3BWm_100, exposure (Exposure_Days_Md) irr
poisson TLI_Alla_Segment_DL DL_Nm3BWm_100, exposure (Exposure_Days_Max) irr

poisson TLI_Alla_Segment_DL AFAQ, exposure (Exposure_Days_Min) irr
poisson TLI_Alla_Segment_DL AFAQ, exposure (Exposure_Days_Md) irr
poisson TLI_Alla_Segment_DL AFAQ, exposure (Exposure_Days_Max) irr

poisson TLI_Alla_Segment_DL PSS14, exposure (Exposure_Days_Min) irr
poisson TLI_Alla_Segment_DL PSS14, exposure (Exposure_Days_Md) irr
poisson TLI_Alla_Segment_DL PSS14, exposure (Exposure_Days_Max) irr

poisson TLI_Alla_Segment_DL PSQI, exposure (Exposure_Days_Min) irr
poisson TLI_Alla_Segment_DL PSQI, exposure (Exposure_Days_Md) irr
poisson TLI_Alla_Segment_DL PSQI, exposure (Exposure_Days_Max) irr

poisson TLI_Alla_Segment_DL GAD7, exposure (Exposure_Days_Min) irr
poisson TLI_Alla_Segment_DL GAD7, exposure (Exposure_Days_Md) irr
poisson TLI_Alla_Segment_DL GAD7, exposure (Exposure_Days_Max) irr

//OVERALL CONCLUSION for the offset variable: the use of different exposure does not change the relationship with the outcome variable for each individual covariates therefore Exposure_Days_Md will be used/////////////
```

//B.The outcome variable and zero inflation

tab TLI_Alla_Segment_NDL

tab TLI_Alla_Segment_DL //both DL and NDL has a lot of zeroes=people that do not have an injury

//OVERALL CONCLUSION for zero inflation: There is no theoretical risk for zero inflation and structural zeros in this data set.(structural zeros=the subject cannot get an injury due to some reason. Theoretical risk that a subject cannot get injured= e.g. the player did not get so much time on the field during the season)///

*STEP 3. CHECKING FOR THE LINEARITY ON THE LOG SCALE

//Evaluating whether the relationship (of each continuous variable) with the outcome variable is linear on the log scale

//OBS! with the command lowess the outcome variable should be evaluated in the log scale and if the curve is approximately straight there is a linear relationship.

//regarding the polynomial test (inclusion of a quadratic term) the quadratic term should not be significant for a relationship between the outcome variable

//and the covariates (Not on the log scale)

//The variable Age

poisson TLI_Alla_Segment_NDL Age, exposure(Exposure_Days_Md) //OBS! no IRR as the test is done on the log scale

predict log_mu, xb

lowess log_mu Age, mean //the curve is approximately straight

drop log_mu

poisson TLI_Alla_Segment_DL Age, exposure(Exposure_Days_Md)

predict log_mu, xb

lowess log_mu Age, mean //the curve is not fully straight from 16 to 20 years.

drop log_mu

gen Age2 = Age^2

```
poisson TLI_Alla_Segment_NDL Age Age2, exposure(Exposure_Days_Md) irr // n.s
poisson TLI_Alla_Segment_DL Age Age2, exposure(Exposure_Days_Md) irr // n.s
drop Age2

//The variable Weightkg
poisson TLI_Alla_Segment_NDL Weightkg, exposure(Exposure_Days_Md)
predict log_mu, xb
lowess log_mu Weightkg, mean //the curve is linear
drop log_mu

poisson TLI_Alla_Segment_DL Weightkg, exposure(Exposure_Days_Md)
predict log_mu, xb
lowess log_mu Weightkg, mean //the curve is linear
drop log_mu

gen Weightkg2 = Weightkg^2
poisson TLI_Alla_Segment_NDL Weightkg Weightkg2, exposure(Exposure_Days_Md) irr //
n.s
poisson TLI_Alla_Segment_DL Weightkg Weightkg2, exposure(Exposure_Days_Md) irr //
n.s
drop Weightkg2

//The variable Lengthm
poisson TLI_Alla_Segment_NDL Lengthm, exposure(Exposure_Days_Md)
predict log_mu, xb
lowess log_mu Lengthm, mean //the curve is relatively straight
drop log_mu

poisson TLI_Alla_Segment_DL Lengthm, exposure(Exposure_Days_Md)
predict log_mu, xb
lowess log_mu Lengthm, mean //the curve is relatively straight
drop log_mu
```

```
gen Lengthm2 = Lengthm^2
poisson TLI_Alla_Segment_NDL Lengthm Lengthm2, exposure(Exposure_Days_Md) irr //
n.s
poisson TLI_Alla_Segment_DL Lengthm Lengthm2, exposure(Exposure_Days_Md) irr // n.s
drop Lengthm2

//The variable BMI
poisson TLI_Alla_Segment_NDL BMI, exposure(Exposure_Days_Md)
predict log_mu, xb
lowess log_mu BMI, mean //the curve is relatively straight
drop log_mu

poisson TLI_Alla_Segment_DL BMI, exposure(Exposure_Days_Md)
predict log_mu, xb
lowess log_mu BMI, mean //the curve is relatively straight
drop log_mu

gen BMI2 = BMI^2
poisson TLI_Alla_Segment_NDL BMI BMI2, exposure(Exposure_Days_Md) irr // n.s
poisson TLI_Alla_Segment_DL BMI BMI2, exposure(Exposure_Days_Md) irr // n.s
drop BMI2


//The variable ADF
poisson TLI_Alla_Segment_NDL NDL_ADF, exposure(Exposure_Days_Md)
predict log_mu, xb
lowess log_mu NDL_ADF, mean //the curve is relatively straight
drop log_mu

poisson TLI_Alla_Segment_DL DL_ADF, exposure(Exposure_Days_Md)
predict log_mu, xb
```

lowess log_mu DL_ADF, mean //the curve is relatively straight

drop log_mu

gen NDL_ADF2=NDL_ADF^2

poisson TLI_Alla_Segment_NDL NDL_ADF NDL_ADF2, exposure(Exposure_Days_Md) irr // n.s

drop NDL_ADF2

gen DL_ADF2=DL_ADF^2

poisson TLI_Alla_Segment_NDL DL_ADF DL_ADF2, exposure(Exposure_Days_Md) irr // n.s

drop DL_ADF2

//The variable CLAM (

poisson TLI_Alla_Segment_NDL NDL_Nm3BWm_100, exposure(Exposure_Days_Md)

predict log_mu, xb

lowess log_mu NDL_Nm3BWm_100, mean //the curve is not totally straight but has an overall linear appearance

drop log_mu

poisson TLI_Alla_Segment_DL DL_Nm3BWm_100, exposure(Exposure_Days_Md)

predict log_mu, xb

lowess log_mu DL_Nm3BWm_100, mean //the curve is not totally straight but has an overall linear appearance

drop log_mu

gen NDL_Nm3BWm_100_2=NDL_Nm3BWm_100^2

poisson TLI_Alla_Segment_NDL NDL_Nm3BWm_100 NDL_Nm3BWm_100_2, exposure(Exposure_Days_Md) irr // n.s

drop NDL_Nm3BWm_100_2

gen DL_Nm3BWm_100_2=DL_Nm3BWm_100^2

poisson TLI_Alla_Segment_DL DL_Nm3BWm_100 DL_Nm3BWm_100_2, exposure(Exposure_Days_Md) irr // n.s

```
drop DL_Nm3BWm_100_2

//The variable AFAQ
poisson TLI_Alla_Segment_NDL AFAQ, exposure(Exposure_Days_Md)
predict log_mu, xb
lowess log_mu AFAQ, mean //the curve is relatively straight
drop log_mu

poisson TLI_Alla_Segment_DL AFAQ, exposure(Exposure_Days_Md)
predict log_mu, xb
lowess log_mu AFAQ, mean //the curve is relatively straight
drop log_mu

gen AFAQ2=AFAQ^2
poisson TLI_Alla_Segment_NDL AFAQ AFAQ2, exposure(Exposure_Days_Md) irr // n.s
drop AFAQ2

gen AFAQ2=AFAQ^2
poisson TLI_Alla_Segment_DL AFAQ AFAQ2, exposure(Exposure_Days_Md) irr // n.s
drop AFAQ2

//The variable PSS14
poisson TLI_Alla_Segment_NDL PSS14, exposure(Exposure_Days_Md)
predict log_mu, xb
lowess log_mu PSS14, mean //the curve is relatively straight
drop log_mu

poisson TLI_Alla_Segment_DL PSS14, exposure(Exposure_Days_Md)
predict log_mu, xb
lowess log_mu PSS14, mean //the curve is relatively straight
drop log_mu
```

```
gen PSS142=PSS14^2
poisson TLI_Alla_Segment_NDL PSS14 PSS142, exposure(Exposure_Days_Md) irr // n.s
drop PSS142

gen PSS142=PSS14^2
poisson TLI_Alla_Segment_DL PSS14 PSS142, exposure(Exposure_Days_Md) irr // n.s
drop PSS142


//The variable GAD-7
poisson TLI_Alla_Segment_NDL GAD7, exposure(Exposure_Days_Md) //
predict log_mu, xb
lowess log_mu GAD7, mean // the curve is relatively straight
drop log_mu

poisson TLI_Alla_Segment_DL GAD7, exposure(Exposure_Days_Md)
predict log_mu, xb
lowess log_mu GAD7, mean //the curve is relatively straight
drop log_mu

gen GAD72=GAD7^2
poisson TLI_Alla_Segment_NDL GAD7 GAD72, exposure(Exposure_Days_Md) irr // n.s
drop PSS142

gen GAD72=GAD7^2
poisson TLI_Alla_Segment_DL GAD7 GAD72, exposure(Exposure_Days_Md) irr // n.s
drop GAD72

//The variable PSQI
poisson TLI_Alla_Segment_NDL PSQI, exposure(Exposure_Days_Md) //
predict log_mu, xb
lowess log_mu PSQI, mean // the curve is relatively straight
```

drop log_mu

poisson TLI_Alla_Segment_DL PSQI, exposure(Exposure_Days_Md)

predict log_mu, xb

lowess log_mu PSQI, mean //the curve is relatively straight

drop log_mu

gen PSQI2=PSQI^2

poisson TLI_Alla_Segment_NDL PSQI PSQI2, exposure(Exposure_Days_Md) irr // n.s

drop PSQI2

gen PSQI2=PSQI^2

poisson TLI_Alla_Segment_DL PSQI PSQI2, exposure(Exposure_Days_Md) irr // n.s

drop PSQI2

//OVERALL CONCLUSION FOR THE EVALUATION OF THE LINEAR RELATIONSHIP WITH THE OUTCOME VARIABLE ON THE LOG SCALE:

//The continuous variables seem to have a linear relationship with the outcome variable on the log scale. However, as age is categorized in the study of overuse injuries it will also be categorized in the study of TLI//

*STEP 4. CHECKING FOR COLLINEARITY

//To evaluate the values:

//VIF > 10 = a strong collinearity

//VIF > 5 = might be worrying, especially if there is a lot of high values

//Tolerance (= 1/VIF), low values (< 0.1) might be a red flag.

//A. starting with an overall check of the continuous and categorical variables.

pwcorr Age Weightkg Lengthm BMI NDL_Nm3BWm_100 NDL_ADF AFAQ PSS14,sig

pwcorr Age Weightkg Lengthm BMI DL_Nm3BWm_100 DL_ADF AFAQ PSS14,sig

pwcorr Age Weightkg BMI NDL_Nm3BWm_100 NDL_ADF AFAQ PSS14,sig //BMI and weight 0.75

pwcorr Age Weightkg BMI DL_Nm3BWm_100 DL_ADF AFAQ PSS14,sig

pwcorr Age Lengthm BMI NDL_Nm3BWm_100 NDL_ADF AFAQ PSS14,sig

pwcorr Age Lengthm BMI DL_Nm3BWm_100 DL_ADF AFAQ PSS14,sig

pwcorr Age BMI NDL_Nm3BWm_100 NDL_ADF AFAQ PSS14,sig

pwcorr Age BMI DL_Nm3BWm_100 DL_ADF AFAQ PSS14,sig //as I know length and weight has a correlation to BMI those are eliminated. Length by itself is not correlated

//correlated to BMI so it could be left in the model for now. All correlation coefficients are <0.8.

tab div NDL_SLSfailpass, chi2 //p=0.06

tab div DL_SLSfailpass, chi2 //p=0.26

tab div PSQI_cat2, chi2 //p=0.26

tab div GAD7_cat2, chi2 //p=0.20

tab NDL_SLSfailpass PSQI_cat2, chi2 //p=0.20

tab DL_SLSfailpass PSQI_cat2, chi2 //p=0.38

tab NDL_SLSfailpass GAD7_cat2, chi2 //p=0.98

tab DL_SLSfailpass GAD7_cat2, chi2 //p=0.15

tab PSQI_cat2 GAD7_cat2, chi2 //p=0.000

//chi2 is not a correlation measure but says something about the association between two variables. PSQI and GAD has a strong association and should be kept in mind for the estat vif test.//

//B. A more specific test for collinearity.

regress TLI_Alla_Segment_NDL div Age Weightkg Lengthm BMI NDL_SLSfailpass NDL_ADF NDL_Nm3BWm_100 AFAQ PSS14 PSQI_cat2 GAD7_cat2

estat vif

regress TLI_Alla_Segment_NDL div Age Lengthm BMI NDL_SLSfailpass NDL_ADF NDL_Nm3BWm_100 AFAQ PSS14 PSQI_cat2 GAD7_cat2

estat vif

regress TLI_Alla_Segment_NDL div Age Weightkg BMI NDL_SLSfailpass NDL_ADF NDL_Nm3BWm_100 AFAQ PSS14 PSQI_cat2 GAD7_cat2

estat vif

regress TLI_Alla_Segment_NDL div Age BMI NDL_SLSfailpass NDL_ADF NDL_Nm3BWm_100 AFAQ PSS14 PSQI_cat2 GAD7_cat2

estat vif

regress TLI_Alla_Segment_NDL div Age BMI NDL_SLSfailpass NDL_ADF NDL_Nm3BWm_100 AFAQ PSS14 PSQI GAD7 //GAD and PSQI as continuous variables

estat vif

//High VIF values over 100 when weight, length and BMI are included in the same calculation.

//If BMI and length is combined the VIF is 1.0 and if weight and BMI are combined VIF is 2.5. //Conclusion: BMI can in a full model be combined with either weight or length but not for all three together

regress TLI_Alla_Segment_DL div Age Weightkg Lengthm BMI DL_SLSfailpass DL_ADF DL_Nm3BWm_100 AFAQ PSS14 PSQI_cat2 GAD7_cat2

estat vif

regress TLI_Alla_Segment_DL div Age Lengthm BMI DL_SLSfailpass DL_ADF DL_Nm3BWm_100 AFAQ PSS14 PSQI_cat2 GAD7_cat2

estat vif

regress TLI_Alla_Segment_DL div Age Weightkg BMI DL_SLSfailpass DL_ADF DL_Nm3BWm_100 AFAQ PSS14 PSQI_cat2 GAD7_cat2

estat vif

regress TLI_Alla_Segment_DL div Age BMI DL_SLSfailpass DL_ADF DL_Nm3BWm_100 AFAQ PSS14 PSQI_cat2 GAD7_cat2

estat vif

regress TLI_Alla_Segment_DL div Age BMI DL_SLSfailpass DL_ADF DL_Nm3BWm_100 AFAQ PSS14 PSQI GAD7 //GAD and PSQI as continuous variables

estat vif

//High VIF values over 100 when weight, length and BMI are included in the same calculation. If BMI and length is combined the VIF is 1.0 and if weight and BMI are combined VIF is 2.5. Conclusion: BMI can in a full model be combined with either weight or length but not for all three together//

*STEP 5. OVERDISPERSION

//If the variance is not equal to the mean you have an overdispersion, Var(Y)>E(Y). If so, there is a risk to underestimate the SE and to receive small p-values.

//if so, the solution is to change to a nbreg or to correct the SE with vce(robust)

//A. Looking at the crude values for the outcome variable

summarize TLI_Alla_Segment_NDL, detail

summarize TLI_Alla_Segment_DL, detail //Conclusion: The mean is approximately equal to the variance no sign for overdispersion

//B. Comparing the Likelihood ratio-test (Poisson) with the negative binominal regression (nbreg), this should be tested with a "reasonable" and "realistic" presumptive Model.

//To evaluate the output:

//Is alpha = 0 ??. If the hypothesis is rejected (p=<0.05) there is overdispersion and I should use a nbreg, if the hypothesis cannot be rejected (p>0.05)

//there is no overdispersion and a poisson regression is enough.

//α ≈ 0 → almost no overdispersion. α ≈ 0.1–0.5 → weak overdispersion, Poisson could be ok sometimes. α > 1 → obvious overdispersion nbreg much better.

//α several times larger than 1 → extreme overdispersion.

nbreg TLI_Alla_Segment_NDL div Age Lengthm BMI NDL_SLSfailpass NDL_ADF NDL_Nm3BWm_100 AFAQ PSS14 PSQI_cat2 GAD7_cat2, exposure(Exposure_Days_Md) irr //alpha=1.78 , p=0.004

nbreg TLI_Alla_Segment_NDL div Age Lengthm BMI NDL_SLSfailpass NDL_ADF NDL_Nm3BWm_100 AFAQ PSS14 PSQI GAD7, exposure(Exposure_Days_Md) irr //alpha=1.62 , p=0.007

nbreg TLI_Alla_Segment_NDL div Age Weightkg BMI NDL_SLSfailpass NDL_ADF NDL_Nm3BWm_100 AFAQ PSS14 PSQI_cat2 GAD7_cat2, exposure(Exposure_Days_Md) irr //alpha=1.76 , p=0.004

nbreg TLI_Alla_Segment_NDL div Age Weightkg BMI NDL_SLSfailpass NDL_ADF NDL_Nm3BWm_100 AFAQ PSS14 PSQI GAD7, exposure (Exposure_Days_Md) irr //alpha=1.60 , p=0.007

nbreg TLI_Alla_Segment_NDL div Age BMI NDL_SLSfailpass NDL_ADF NDL_Nm3BWm_100 AFAQ PSS14 PSQI_cat2 GAD7_cat2, exposure(Exposure_Days_Md) irr //alpha=1.68 , p=0.005

nbreg TLI_Alla_Segment_NDL div Age BMI NDL_SLSfailpass NDL_ADF NDL_Nm3BWm_100 AFAQ PSS14 PSQI GAD7, exposure(Exposure_Days_Md) irr //alpha=1.52 , p=0.009

nbreg TLI_Alla_Segment_DL div Age Lengthm BMI NDL_SLSfailpass DL_ADF DL_Nm3BWm_100 AFAQ PSS14 PSQI_cat2 GAD7_cat2, exposure(Exposure_Days_Md) irr //alpha=0.67 , p=0.19

nbreg TLI_Alla_Segment_DL div Age Lengthm BMI NDL_SLSfailpass DL_ADF DL_Nm3BWm_100 AFAQ PSS14 PSQI GAD7, exposure(Exposure_Days_Md) irr //alpha=0.93 , p=0.13

nbreg TLI_Alla_Segment_DL div Age Weightkg BMI DL_SLSfailpass DL_ADF DL_Nm3BWm_100 AFAQ PSS14 PSQI_cat2 GAD7_cat2, exposure(Exposure_Days_Md) irr //alpha=0.059 , p=0.47

nbreg TLI_Alla_Segment_DL div Age Weightkg BMI DL_SLSfailpass DL_ADF DL_Nm3BWm_100 AFAQ PSS14 PSQI GAD7, exposure(Exposure_Days_Md) irr //alpha=0.45 , p=0.30

nbreg TLI_Alla_Segment_DL div Age BMI DL_SLSfailpass DL_ADF DL_Nm3BWm_100 AFAQ PSS14 PSQI_cat2 GAD7_cat2, exposure(Exposure_Days_Md) irr //alpha=0.000 , p=0.5

nbreg TLI_Alla_Segment_DL div Age BMI DL_SLSfailpass DL_ADF DL_Nm3BWm_100 AFAQ PSS14 PSQI GAD7, exposure(Exposure_Days_Md) irr //alpha=0.43 , p=0.33

//Conclusion: The NDL show overdispersion but the DL does not in one of the models

//C. Comparing the deviance/ Pearson chi2 with df

//if the p values are >0.05 there is no overdispersion.

//You can also look at Goodness of fit/df. If =0 to 1, no overdispersion, if =1.5 obvious overdispersion, if <1 under dispersion

poisson TLI_Alla_Segment_NDL div Age Lengthm BMI NDL_SLSfailpass NDL_ADF NDL_Nm3BWm_100 AFAQ PSS14 PSQI_cat2 GAD7_cat2, exposure(Exposure_Days_Md) irr

estat gof, pearson // Goodness of fit=437, Df=230,p=0.0000-->overdispersion

poisson TLI_Alla_Segment_NDL div Age Lengthm BMI NDL_SLSfailpass NDL_ADF NDL_Nm3BWm_100 AFAQ PSS14 PSQI GAD7, exposure(Exposure_Days_Md) irr

estat gof, pearson

poisson TLI_Alla_Segment_NDL div Age Weightkg BMI NDL_SLSfailpass NDL_ADF NDL_Nm3BWm_100 AFAQ PSS14 PSQI_cat2 GAD7_cat2, exposure(Exposure_Days_Md) irr

estat gof, pearson // Approximately as above

poisson TLI_Alla_Segment_NDL div Age Weightkg BMI NDL_SLSfailpass NDL_ADF NDL_Nm3BWm_100 AFAQ PSS14 PSQI GAD7, exposure(Exposure_Days_Md) irr

estat gof, pearson

poisson TLI_Alla_Segment_NDL div Age BMI NDL_SLSfailpass NDL_ADF NDL_Nm3BWm_100 AFAQ PSS14 PSQI_cat2 GAD7_cat2, exposure(Exposure_Days_Md) irr

estat gof, pearson // Approximately as above

poisson TLI_Alla_Segment_NDL div Age BMI NDL_SLSfailpass NDL_ADF NDL_Nm3BWm_100 AFAQ PSS14 PSQI GAD7, exposure(Exposure_Days_Md) irr

estat gof, pearson

poisson TLI_Alla_Segment_DL div Age Lengthm BMI NDL_SLSfailpass DL_ADF DL_Nm3BWm_100 AFAQ PSS14 PSQI_cat2 GAD7_cat2, exposure(Exposure_Days_Md) irr

estat gof, pearson //Goodness of fit=493, Df=229,p=0.0000-->overdispersion

poisson TLI_Alla_Segment_DL div Age Lengthm BMI NDL_SLSfailpass DL_ADF DL_Nm3BWm_100 AFAQ PSS14 PSQI GAD7, exposure(Exposure_Days_Md) irr

estat gof, pearson

poisson TLI_Alla_Segment_DL div Age Weightkg BMI DL_SLSfailpass DL_ADF DL_Nm3BWm_100 AFAQ PSS14 PSQI_cat2 GAD7_cat2, exposure(Exposure_Days_Md) irr

estat gof, pearson ////Goodness of fit=531, Df=229,p=0.0000-->overdispersion

poisson TLI_Alla_Segment_DL div Age Weightkg BMI DL_SLSfailpass DL_ADF DL_Nm3BWm_100 AFAQ PSS14 PSQI GAD7, exposure(Exposure_Days_Md) irr

estat gof, pearson

poisson TLI_Alla_Segment_DL div Age BMI DL_SLSfailpass DL_ADF DL_Nm3BWm_100 AFAQ PSS14 PSQI_cat2 GAD7_cat2, exposure(Exposure_Days_Md) irr

estat gof, pearson // Approximately as above

poisson TLI_Alla_Segment_DL div Age BMI DL_SLSfailpass DL_ADF DL_Nm3BWm_100 AFAQ PSS14 PSQI GAD7, exposure(Exposure_Days_Md) irr

estat gof, pearson

//OVERALL CONCLUSION: There is an obvious overdispersion and therefore nbreg will be used instead of a poisson regression. //OBS!GOF test has been done on the Model without and without categorization of GAD and PSQI which gives the same results

*STEP 5. UNIVARIATE MODELS

//Given that the data contained clustered observations due to repeated measurements per individual, all analyses employed

//robust standard errors with clustering at the individual level (vce(cluster Subject)). nbreg is also used due to overdispersion

// a. Division

nbreg TLI_Alla_Segment_NDL div, exposure(Exposure_Days_Md) vce(cluster Subject) irr

nbreg TLI_Alla_Segment_NDL i.div, exposure(Exposure_Days_Md) vce(cluster Subject) irr

nbreg TLI_Alla_Segment_DL div, exposure(Exposure_Days_Md) vce(cluster Subject) irr

nbreg TLI_Alla_Segment_DL i.div, exposure(Exposure_Days_Md) vce(cluster Subject) irr

//b. Age

nbreg TLI_Alla_Segment_NDL Age_cat, exposure(Exposure_Days_Md) vce(cluster Subject) irr

nbreg TLI_Alla_Segment_NDL i.Age_cat, exposure(Exposure_Days_Md) vce(cluster Subject) irr

nbreg TLI_Alla_Segment_DL Age_cat, exposure(Exposure_Days_Md) vce(cluster Subject) irr

nbreg TLI_Alla_Segment_DL i.Age_cat, exposure(Exposure_Days_Md) vce(cluster Subject) irr

//c. Length

nbreg TLI_Alla_Segment_NDL Length100, exposure(Exposure_Days_Md) vce(cluster Subject) irr

nbreg TLI_Alla_Segment_DL Length100, exposure(Exposure_Days_Md) vce(cluster Subject) irr

//d. BMI

nbreg TLI_Alla_Segment_NDL BMI, exposure(Exposure_Days_Md) vce(cluster Subject) irr

nbreg TLI_Alla_Segment_DL BMI, exposure(Exposure_Days_Md) vce(cluster Subject) irr

//e. SLS fail/pass

nbreg TLI_Alla_Segment_NDL NDL_SLSfailpass, exposure(Exposure_Days_Md) vce(cluster Subject) irr

nbreg TLI_Alla_Segment_DL DL_SLSfailpass, exposure(Exposure_Days_Md) vce(cluster Subject) irr

//f. ADF

nbreg TLI_Alla_Segment_NDL NDL_ADF , exposure(Exposure_Days_Md) vce(cluster Subject) irr

nbreg TLI_Alla_Segment_DL DL_ADF , exposure(Exposure_Days_Md) vce(cluster Subject) irr

//g. CLAM

nbreg TLI_Alla_Segment_NDL NDL_Nm3BWm_100, exposure(Exposure_Days_Md) vce(cluster Subject) irr

nbreg TLI_Alla_Segment_DL DL_Nm3BWm_100, exposure(Exposure_Days_Md) vce(cluster Subject) irr

//h. AFAQ

nbreg TLI_Alla_Segment_NDL AFAQ , exposure(Exposure_Days_Md) vce(cluster Subject) irr

nbreg TLI_Alla_Segment_DL AFAQ , exposure(Exposure_Days_Md) vce(cluster Subject) irr

//i. PSS14

nbreg TLI_Alla_Segment_NDL PSS14 , exposure(Exposure_Days_Md) vce(cluster Subject) irr

nbreg TLI_Alla_Segment_DL PSS14 , exposure(Exposure_Days_Md) vce(cluster Subject) irr

//j. PSQI

nbreg TLI_Alla_Segment_NDL PSQI_cat2 , exposure(Exposure_Days_Md) vce(cluster Subject) irr

nbreg TLI_Alla_Segment_DL PSQI_cat2 , exposure(Exposure_Days_Md) vce(cluster Subject) irr

//k. GAD7

nbreg TLI_Alla_Segment_NDL GAD7_cat2 , exposure(Exposure_Days_Md) vce(cluster Subject) irr

nbreg TLI_Alla_Segment_NDL i.GAD7_cat2 , exposure(Exposure_Days_Md) vce(cluster Subject) irr

nbreg TLI_Alla_Segment_DL GAD7_cat2 , exposure(Exposure_Days_Md) vce(cluster Subject) irr

nbreg TLI_Alla_Segment_DL i.GAD7_cat2 , exposure(Exposure_Days_Md) vce(cluster Subject) irr

*MODEL BUILDING WITH PURPOSEFUL SELECTION FOR TIME-LOSS INJURY: NON-DOMINANT LEG

*STEP 0. Candidate list

//div: P=0.03 for one division. The variable stays

//Age: An apriori variable selected from the literature (will be categorized as it is so in the study or overuse injuries)

//Length: P=0.45 and is therefore eliminated

//BMI: P=0.39 and is therefore eliminated

//The SLS: P=0.80 and is therefore eliminated

//ADF: P=0.04. The variable stays

//CLAM: P=0.02. The variable stays

//AFAQ: P=0.2. The variable stays

//PSS-14:P=0.92 and is therefore eliminated

//PSQI: P=0.95 and is therefore eliminated

//GAD-7: P=0.21 The variable stays

*STEP 1. Full base Model

//Starting with a full Model and saves AIC and BIC. AIC/BIC is a reference point. If a reduced model has equal or lower AIC/BIC it is a better balance between

//fit and simplicity. If AIC/BIC increases when a variable is eliminated it probably contributed to the Model even though the p-value was low.

nbreg TLI_Alla_Segment_NDL i.div i.Age_cat NDL_ADF NDL_Nm3BWm_100 AFAQ GAD7,exposure(Exposure_Days_Md) vce(cluster Subject) irr

est store full_1_noCAT

estat ic //AIC=294.7962 BIC=329.6856, Wald=22.77 and pseudo R2=0.699

*STEP 2. Wald test and confounding check for each variable

*AFAQ

//a. Wald test:

//Eliminating and testing one variable at a time with the wald test (LR test is invalid for models with robust vce).

//If p<0.05 the variable contributes significantly and it should stay in the model.

//If p>0.05 the variable does not contribute to the model and could be eliminated.

est store full_1_noCAT

testparm AFAQ //p=0.4776

//b. Confounding-check

//Even though a variable is non-significant, it could be a confounder and should therefore stay in the Model

//The rule of thumb is that a change of 10% or more of the IRR is a confounder (comparing the full Model with the reduces Model)

nbreg TLI_Alla_Segment_NDL i.div i.Age_cat NDL_ADF NDL_Nm3BWm_100 GAD7,exposure(Exposure_Days_Md) vce(cluster Subject) irr //full Model without AFAQ

est store reduced_1_noCAT

est table full_1_noCAT reduced_1_noCAT, eform b(%9.3f) se(%9.3f) stats(N ll) //no changes >10% is noticed. The variable AFAQ is eliminated

//c. new model without AFAQ

nbreg TLI_Alla_Segment_NDL i.div i.Age_cat NDL_ADF NDL_Nm3BWm_100 GAD7,exposure(Exposure_Days_Md) vce(cluster Subject) irr //The new full model

est store full_2_noCAT

estat ic //AIC=293.1821 , BIC=324.5826 If AIC and BIC are the same or lower it is a better balance between fit and simplicity which is good. If AIC and BIC rise

//significantly the variable taken away probably contributed to the model even though the p value was poor.

//CONCLUSION: AFAQ is taken away

*GAD-7

//a. Wald test

est store full_2_noCAT

testparm GAD7 //p=0.153

//b. Confounding check

nbreg TLI_Alla_Segment_NDL i.div i.Age_cat NDL_ADF NDL_Nm3BWm_100,exposure(Exposure_Days_Md) vce(cluster Subject) irr //The new full model

est store reduced_2_noCAT

est table full_2_noCAT reduced_2_noCAT, eform b(%9.3f) se(%9.3f) stats(N ll) //no changes >10% is noticed. The variable GAD-7 is eliminated

//c. new model without GAD-7

nbreg TLI_Alla_Segment_NDL i.div i.Age_cat NDL_ADF NDL_Nm3BWm_100,exposure(Exposure_Days_Md) vce(cluster Subject) irr //The new full model

est store full_3_noCAT

estat ic //AIC=293.2522 , BIC=321.1967, Wald=17.02 and Pseudo R2= 0.06

//CONCLUSION: GAD-7 is taken away

*Division

//According to the descriptive analysis this variable has few subjects with >=2 injuries in all divisions. The statistics for the new full model above

//shows that the significant division 2 has a great CI, probably due to this.

//a. Wald test

est store full_3_noCAT

testparm i.div //p=0.0353

//b. Confounding check

nbreg TLI_Alla_Segment_NDL i.Age_cat NDL_ADF NDL_Nm3BWm_100,exposure(Exposure_Days_Md) vce(cluster Subject) irr //The new full model

est store reduced_3_noCAT

est table full_3_noCAT reduced_3_noCAT, eform b(%9.3f) se(%9.3f) stats(N ll) //no changes >10% is noticed.

//c. new model without i.div

nbreg TLI_Alla_Segment_NDL i.Age_cat NDL_ADF NDL_Nm3BWm_100,exposure(Exposure_Days_Md) vce(cluster Subject) irr //The new full model

est store full_4_noCAT

estat ic //AIC=295.5805 , BIC=316.5389, Wald=11.56 and Pseudo R2= 0.04

//Conclusion:

//1. The Wald test shows that i.div contributes to the model and therefore should stay in the model (statistical argument).

//2. The reduced model does not show any greater differences regarding the other variables p value, IRR, CI and SE.

//3. AIC is slightly higher (2 units) in the reduced model and BIC is lower in the reduced model (5 units).

//4. Descriptive statistics shows that in each division there is few subjects that have two or more injuries which probably is reflected //in the greater CI shown in the full model which indicates considerable uncertainty in the estimate. Furthermore, the trend that players //in lower divisions are at higher risk is not totally stringent as there is a 3 times higher IRR in div 2 //compared to div 1 but the ratio is almost 1 and non-significant for div 3.

//5. In a scientifical theoretical perspective division might not be important (no major studies found on that subject and div is //also contextual dependent. in a logical common sense perspective female in higher divisions could have less injuries due to better //skills and better physical ability but on the other hand be at more at risk due to more exposure. To report non stringent results with uncertain //estimates for variables that are contextual dependent might not provide any major advantage. //This means that the reduced model might be slightly less predictive (AIC,less fit) but on the other hand more parsimonious (BIC). //Choosing the reduced model gives a more parsimonious model with approximately the same prediction/fit but without the statistical //uncertainty and non-stringent trend. The disadvantage is that we

lose the information about division in general. //The variable div is taken away from the model

*STEP 3. Interaction terms

//Chooses to test interaction for age x CLAM, age x ADF and ADF x CLAM.

//If the interaction term is significant and/or gives a theoretical reasonable pattern the interaction terms stay in the Model.

//AIC and BIC in the model with interaction terms is compared to the model without interactions, if AIC decreases a lot and the interaction term is theoretical

//reasonable, the terms stay in the Model.

nbreg TLI_Alla_Segment_NDL i.Age_cat NDL_ADF NDL_Nm3BWm_100,exposure(Exposure_Days_Md) vce(cluster Subject) irr

//This is the new full model. //AIC=295.5805 , BIC=316.5389, Wald=11.56 and Pseudo R2= 0.04

nbreg TLI_Alla_Segment_NDL i.Age_cat##c.NDL_Nm3BWm_100 NDL_ADF ,exposure(Exposure_Days_Md) vce(cluster Subject) irr //Age X CLAM=non-significant

estat ic //AIC=295 , BIC=323 AIC and BIC are about the same for AIC but worse for BIC

nbreg TLI_Alla_Segment_NDL i.Age_cat##c.NDL_ADF NDL_Nm3BWm_100, exposure(Exposure_Days_Md) vce(cluster Subject) irr //Age X ADF=non-significant

estat ic //AIC=299.3223 , BIC=327.2668 both AIC and BIC increase a little bit

nbreg TLI_Alla_Segment_NDL i.Age_cat c.NDL_ADF##c.NDL_Nm3BWm_100, exposure(Exposure_Days_Md) vce(cluster Subject) irr //ADF X CLAM=non-significant

estat ic //AIC=297.111 , BIC=321.5625 AIC and BIC increase a little bit

nbreg TLI_Alla_Segment_NDL i.Age_cat NDL_ADF NDL_Nm3BWm_100,exposure(Exposure_Days_Md) vce(cluster Subject) irr //This is the new full model

//CONCLUSION: No interactions seen

//Testing if there would have been any interaction if div was included:

//TEST MED DIV

nbreg TLI_Alla_Segment_NDL i.div i.Age_cat NDL_ADF NDL_Nm3BWm_100,exposure(Exposure_Days_Md) vce(cluster Subject) irr

//AIC=293.2522 , BIC=321.1967, Wald=17.02 and Pseudo R2= 0.06

//Age x div

nbreg TLI_Alla_Segment_NDL i.div##i.Age_cat NDL_ADF NDL_Nm3BWm_100,exposure(Exposure_Days_Md) vce(cluster Subject) irr //non-significant

//age x adf

nbreg TLI_Alla_Segment_NDL i.div i.Age_cat##c.NDL_ADF NDL_Nm3BWm_100,exposure(Exposure_Days_Md) vce(cluster Subject) irr //non-significant

//age x clam

nbreg TLI_Alla_Segment_NDL i.div i.Age_cat##c.NDL_Nm3BWm_100 NDL_ADF ,exposure(Exposure_Days_Md) vce(cluster Subject) irr //non-significant

//div x adf

nbreg TLI_Alla_Segment_NDL i.div##c.NDL_ADF i.Age_cat NDL_Nm3BWm_100,exposure(Exposure_Days_Md) vce(cluster Subject) irr //non-significant

//div x clam

nbreg TLI_Alla_Segment_NDL i.div##c.NDL_Nm3BWm_100 i.Age_cat NDL_ADF ,exposure(Exposure_Days_Md) vce(cluster Subject) irr //non-significant

*STEP 4. Model fit and diagnostic

//Pearson residuals >2 could be deviant and residuals >3 should definitely be scrutinized (is the Model sensitive to outliers)

//First a visual inspection should be done from scatters, secondly the outliers should be listed and scrutinized regarding their data (data input), thirdly a sensitivity

//analysis should be done to see if the conclusions hold up (model robustness) or not (discussion that the Model is affected by outliers)

//Alt 2: A more complete diagnostic where glm with the nbinom-family is used to receive Pearson residuals/deviance and influential statistics

//OBS! this is just for diagnostics and should not replace nbreg Modelling

nbreg TLI_Alla_Segment_NDL i.Age_cat NDL_ADF NDL_Nm3BWm_100,exposure(Exposure_Days_Md) vce(cluster Subject) irr //This is the new full model

//AIC=295.5805 , BIC=316.5389, Wald=11.56 and Pseudo R2= 0.04

glm TLI_Alla_Segment_NDL i.Age_cat NDL_ADF NDL_Nm3BWm_100, family(nbinomial `a') link(log) exposure(Exposure_Days_Md) nolog

predict muhat_glm_NDL_noCAT, mu

predict pearson_glm_NDL_noCAT, pearson

predict devres_glm_NDL_noCAT, deviance

predict hat_glm_NDL_noCAT, hat

predict cook_glm_NDL_noCAT, cooksd

scatter pearson_glm_NDL_noCAT muhat_glm_NDL_noCAT, yline(0) title("Pearson residuals (glm_NDL_noCAT)") // There is a lot of data points >3.

list Subject muhat_glm_NDL_noCAT pearson_glm_NDL_noCAT if abs(pearson_glm_NDL_noCAT) > 3 //Subject 97, 168, 253, 255, 172, 180, 54, 20, 102, 81, 246, 165 are outliers. Subject 262 has missing values

list if Subject == 97 | Subject == 168| Subject ==253 | Subject == 255| Subject == 172| Subject == 180| Subject == 54| Subject == 20| Subject == 102| Subject == 81| Subject ==246| Subject == 165

list Subject TLI_Alla_Segment_NDL Age NDL_Nm3BWm_100 NDL_ADF pearson_glm_NDL_noCAT if Subject == 97 | Subject == 168| Subject ==253 | Subject == 255| Subject == 172| Subject == 180| Subject == 54| Subject == 20| Subject == 102| Subject == 81| Subject ==246| Subject == 165

//Conclusion:No data has been recorded incorrectly. Subject 262 has missing data for CLAM

nbreg TLI_Alla_Segment_NDL i.Age_cat NDL_ADF NDL_Nm3BWm_100, exposure(Exposure_Days_Md) vce(cluster Subject) irr //Exclusion of outliers should be done on the nbreg model with vce

nbreg TLI_Alla_Segment_NDL i.Age_cat NDL_ADF NDL_Nm3BWm_100 if Subject!=97 & Subject!=168 & Subject!=253 & Subject!=255 & Subject!=172 & Subject!=180 &

Subject!=54 & Subject!=20 & Subject!=102 & Subject!=81 & Subject!=246 & Subject!=165, exposure(Exposure_Days_Md) vce(cluster Subject) irr

//Exclusion of all outliers changes the p value from significant to n.s regarding ADF. The direction is still the same

nbreg TLI_Alla_Segment_NDL i.Age_cat NDL_ADF NDL_Nm3BWm_100 if Subject!=246, exposure(Exposure_Days_Md) vce(cluster Subject) irr //changes the p value to n.s

nbreg TLI_Alla_Segment_NDL i.Age_cat NDL_ADF NDL_Nm3BWm_100 if Subject!=102, exposure(Exposure_Days_Md) vce(cluster Subject) irr //changes the p value to n.s

//Conclusion: When eliminating all outliers at once my general conclusion still holds but Pseudo R2 goes from //0.04 to 0.07 which means better model adaption, Wald chi2 goes from 13 to 22 meaning that the explanatory //power of the model is stronger. The p value for ADL is getting N.S. This means that my results are robust regarding //direction but sensitive to some data points as some outliers affect the strength of the causal relationship //(especially subject 246 and 102 who has low ADF and more TLI).

//b. Analysis of deviance residuals

//Deviance residuals: Values greater than 2-3 could indicate deviance.

scatter devres_glm_NDL_noCAT muhat_glm_NDL_noCAT, yline(0) title("Deviance residuals (glm_NDL_noCAT)")

//Residuals are in reasonable limits (<3), the curves have no systematic patterns, no signs of gross misspecification.

//Leverage graphs (hat values):

//How much an observation affects its prediction (if it is an influential point. Pearson and deviance residuals show if an observation

//is an outlier, but leverage shows if the outlier is an influential point, i.e. how much the outlier affect the model),

//2p/n is a simple cut off. in my case: di (2*5)/231=.04. That will say: hat-values greater than 0.1–0.2 could be values to check.

scatter hat_glm_NDL_noCAT muhat_glm_NDL_noCAT, title("Leverage vs. Predicted values") ytitle("Leverage (hat_NDL_noCAT)") xtitle("Predicted counts (muhat_NDL_noCAT)") // There is no data point over 0.1

list Subject hat_glm_NDL_noCAT muhat_glm_NDL_noCAT if hat_glm_NDL_noCAT > 0.2 //

list Subject hat_glm_NDL_noCAT muhat_glm_NDL_noCAT if hat_glm_NDL_noCAT > 0.1 //

//Conclusion: no observations seen for deviance residuals or leverage grafs

//Cook´s distance:

//High estimates of pearson/deviance residuals and high estimates of leverage will give a high cooks d.

//Cooks d tells you how much your model would be affected if one observation was taken away.

//Cook’s D has no definitive cut off. A rule of thumb is that values >1.0 in greater data sets are problematic.

//In smaller data sets as this values 0.05-0.1 could be troublesome and values >0.1 should absolutely be scrutinized.

//Another conservative rule is 4/n. In my case n ≈ 231 → 4/231 ≈ 0.017. so, for this material values from 0.02 could be of interest

scatter cook_glm_NDL_noCAT muhat_glm_NDL_noCAT, title("Cook's distance") //one Subject= 0.15 and the rest are <0.1

sort cook_glm_NDL_noCAT

list Subject Age TLI_Alla_Segment_NDL Exposure_Days_Md NDL_Nm3BWm_100 NDL_ADF if _n > _N-5

// No incorrect data found. 0.15 and 0.1 is higher than normal but still far under the critical limit 1. Subject 81, 253, 102, 246, 262 has high values. 262 is missing data.

nbreg TLI_Alla_Segment_NDL i.Age_cat NDL_ADF NDL_Nm3BWm_100, exposure(Exposure_Days_Md) vce(cluster Subject) irr

nbreg TLI_Alla_Segment_NDL i.Age_cat NDL_ADF NDL_Nm3BWm_100 if Subject!=253 & Subject!=81 & Subject!=102 & Subject!=246, exposure(Exposure_Days_Md) vce(cluster Subject) irr

//Exclusion of those Subjects changes the p value for ADF to be insignificant, p=0.26.

nbreg TLI_Alla_Segment_NDL i.Age_cat NDL_ADF NDL_Nm3BWm_100 if Subject!=253 & Subject!=81 & Subject!=102 & Subject!=246, exposure(Exposure_Days_Md) vce(cluster Subject) irr

nbreg TLI_Alla_Segment_NDL i.Age_cat NDL_ADF NDL_Nm3BWm_100 if Subject!=253 , exposure(Exposure_Days_Md) vce(cluster Subject) irr

nbreg TLI_Alla_Segment_NDL i.Age_cat NDL_ADF NDL_Nm3BWm_100 if Subject!=81 , exposure(Exposure_Days_Md) vce(cluster Subject) irr

nbreg TLI_Alla_Segment_NDL i.Age_cat NDL_ADF NDL_Nm3BWm_100 if Subject!=102, exposure(Exposure_Days_Md) vce(cluster Subject) irr

nbreg TLI_Alla_Segment_NDL i.Age_cat NDL_ADF NDL_Nm3BWm_100 if Subject!=246, exposure(Exposure_Days_Md) vce(cluster Subject) irr

//Conclusion: Subject 246 and 102 affects the Model

//Sort cook's distance

sort cook_glm_NDL_noCAT

list Subject cook_glm_NDL_noCAT muhat_glm_NDL_noCAT pearson_glm_NDL_noCAT devres_glm_NDL_noCAT hat_glm_NDL_noCAT in -10/l //

list Subject cook_glm_NDL_noCAT muhat_glm_NDL_noCAT pearson_glm_NDL_noCAT devres_glm_NDL_noCAT hat_glm_NDL_noCAT if cook_glm_NDL_noCAT > 0.05

//Subject 20, 165, 81, 253, 102, 246 has high values. 246 has 1.5 and the other are <0.1 (as a sensitivity test use this code as it focuses on observations with

//high cooks D. This due to those observations could change the coefficients and p values and this is what I want to check with a sensitivity test)

nbreg TLI_Alla_Segment_NDL i.Age_cat NDL_ADF NDL_Nm3BWm_100 if Subject!=246, exposure(Exposure_Days_Md) vce(cluster Subject) irr //Subject 246 influences the ADF and changes the p value.

nbreg TLI_Alla_Segment_NDL i.Age_cat NDL_ADF NDL_Nm3BWm_100 if Subject!=102, exposure(Exposure_Days_Md) vce(cluster Subject) irr //Subject 102 influences the ADF and changes the p value.

sum NDL_ADF, detail //Max=57, Min= 33. Subject 246 has ADF=35 which is within Q1. In other words, Subject 246 is no real outlier but her observation has a great impact on

//the model.

list Subject muhat_glm_NDL_noCAT hat_glm_NDL_noCAT devres_glm_NDL_noCAT pearson_glm_NDL_noCAT cook_glm_NDL_noCAT if hat_glm_NDL_noCAT > 0.08 | abs(devres_glm_NDL_noCAT) > 3 | abs(pearson_glm_NDL_noCAT) > 3 |cook_glm_NDL_noCAT > 0.05

//both subject 102 and 246 are troublesome (use this code as screening/diagnostic as it shows all types of observations that could be problematic)

//CONCLUSION: Subject 246 and 102 affect the p value for ADL but not the direction.

//Subject 20, 165, 81 and 253 gives the model a better fit (about the same Pseudo R^2 and log likelihood but better AIC and BIC). //This means that those are influential cases but does not affect the significance or direction of the Model. //But Subject 246 does!!

//It is therefore important to address this in the study: to say that our sensitivity analysis for robustness showed that //the effect of ADF is not totally stable. So, the results for ADF should be interpreted with caution//In other words, the model are robust regarding the direction but a little bit sensitive regarding //significance where some data points affect the p value.

//c. Specification Test-Link test

//The link test could indicate that the model is not an appropriate representation of the relationship between the outcome and predictors (misspecification), and furthermore that

//important predictors have been omitted or are represented incorrectly in the model. Hatsq should be n.s and hat should be significant. However, In the literature it is

//mentioned that _hat should be significant since it is the predicted value for the model. In a negative binomial regression Model the pseudo R2 most often are very low

//(0.05-0.10), see STATA manual or Hilbe 2011. Hilbe discuss the general weak predictive value in NB-models, especially with overdispersion and many zeroes.

nbreg TLI_Alla_Segment_NDL i.Age_cat NDL_ADF NDL_Nm3BWm_100, exposure(Exposure_Days_Md) vce(cluster Subject) irr

linktest //hatsq p=0.018, hat p=0.021.Some misspecification is seen

*Conclusion: The model is mis specified and different solutions are tested below

//d. other test to see what misses the model

glm TLI_Alla_Segment_NDL i.Age_cat NDL_ADF NDL_Nm3BWm_100, family(nbinomial) link(log) exposure(Exposure_Days_Md)

linktest //both hatsq and hat are significant

nbreg TLI_Alla_Segment_NDL i.div i.Age_cat NDL_ADF NDL_Nm3BWm_100, exposure(Exposure_Days_Md) vce(cluster Subject) irr

linktest //hatsq p=0.11, hat p=0.26. i.e. if div is included in the model the specificity of the Model improves.

nbreg TLI_Alla_Segment_NDL i.Age_cat NDL_ADF NDL_Nm3BWm_100 if Subject!=246 & Subject!=102, exposure(Exposure_Days_Md) vce(cluster Subject) irr

linktest //both hastq and hat are non-significant

*CONCLUSION: the model will be better specified if the previous div will be included

//e. The new Model will be tested for cooks d to see if there are some changes. There is no reason to test pearson, residual deviance ant leverage one more time

nbreg TLI_Alla_Segment_NDL i.Age_cat i.div NDL_ADF NDL_Nm3BWm_100,exposure(Exposure_Days_Md) vce(cluster Subject) irr //This is the new full model

//AIC=293.2522 , BIC=321.1967, Wald=17 and Pseudo R2= 0.06

glm TLI_Alla_Segment_NDL i.Age_cat i.div NDL_ADF NDL_Nm3BWm_100, family(nbinomial `a') link(log) exposure(Exposure_Days_Md) nolog

predict muhat_glm_NDL_div_noCAT, mu

predict pearson_glm_NDL_div_noCAT, pearson

predict devres_glm_NDL_div_noCAT, deviance

predict hat_glm_NDL_div_noCAT, hat

predict cook_glm_NDL_div_noCAT, cooksd

//Sort cook's distance

sort cook_glm_NDL_div_noCAT

list Subject cook_glm_NDL_div_noCAT muhat_glm_NDL_div_noCAT pearson_glm_NDL_div_noCAT devres_glm_NDL_div_noCAT hat_glm_NDL_div_noCAT in -10/l //

list Subject cook_glm_NDL_div_noCAT muhat_glm_NDL_div_noCAT pearson_glm_NDL_div_noCAT devres_glm_NDL_div_noCAT hat_glm_NDL_div_noCAT if cook_glm_NDL_div_noCAT > 0.05

//Subject 20, 165, 81, 102, 246 has high values. 246 has 0.089 and 102 has 0.069

nbreg TLI_Alla_Segment_NDL i.Age_cat i.div NDL_ADF NDL_Nm3BWm_100 if Subject!=246, exposure(Exposure_Days_Md) vce(cluster Subject) irr //Subject 246 influences the ADF and changes the p value.

nbreg TLI_Alla_Segment_NDL i.Age_cat i.div NDL_ADF NDL_Nm3BWm_100 if Subject!=102, exposure(Exposure_Days_Md) vce(cluster Subject) irr //Subject 102 influences the ADF and changes the p value.

list Subject muhat_glm_NDL_div_noCAT hat_glm_NDL_div_noCAT devres_glm_NDL_div_noCAT pearson_glm_NDL_div_noCAT cook_glm_NDL_div_noCAT if hat_glm_NDL_div_noCAT > 0.08 | abs(devres_glm_NDL_div_noCAT) > 3 | abs(pearson_glm_NDL_div_noCAT) > 3 |cook_glm_NDL_div_noCAT >0.05

//both subject 102 and 246 are troublesome

**CONCLUSION:

//After including div in the model, influence diagnostics showed a general reduction in Cook's distance values/Subjects 102 and 246, which previously had the highest influence (Cook's D = 0.09–0.16), showed markedly lower values (0.07 and 0.09, respectively),

//indicating that the inclusion of div improved model specification and reduced undue influence. Although some observations Subjects 20, 81, and 165)still exhibited large residuals (|Pearson| > 4), their leverage remained low and they //exerted limited influence on model coefficients.

//As ADF is affected of one specifik observation, a further analysis is done here

nbreg TLI_Alla_Segment_NDL i.Age_cat i.div NDL_ADF NDL_Nm3BWm_100,exposure(Exposure_Days_Md) vce(cluster Subject) irr

margins, at(NDL_ADF=(0(5)45)) predict(n)

marginsplot, x(NDL_ADF) ytitle("Predicted number of injuries") xtitle("NDL_ADF") title("Predicted number of injuries by NDL_ADF") ylabel(, angle(horizontal))

*THIS IS THE FINAL MODEL FOR TLI NON-DOMINANT LEG:

nbreg TLI_Alla_Segment_NDL i.Age_cat i.div NDL_ADF NDL_Nm3BWm_100,exposure(Exposure_Days_Md) vce(cluster Subject) irr

//OBS!! For the method in the article, it is important to notice that our sensitivty analysis for robustness showed that the effect of

//ADF is not totally stable. So, the results for ADF should be interpreted with caution. See a written proposal off that in the word document

//"Analys av outliers I slutgiltig Model". In other words the model is robust regarding direction but are a little bit sensitive regarding significance meaning

//that some data points affect the model's precision.//AIC=293.2522 , BIC=321.1967, Wald=17 and Pseudo R2= 0.06

*MODEL BUILDING WITH PURPOSEFUL SELECTION FOR TIME-LOSS INJURY: DOMINANT LEG

*STEP 0. Candidate list

//div: P=0.44, the variable is excluded.

//Age: An apriori variable selected from the literature (will be categorized as it is so in the study or overuse injuries)

//Length: P=0.36, the variable is excluded

//BMI: p=0.56, the variable is excluded

//The SLS: P=0.34, the variable is excluded

//ADF: P=0.84, the variable is excluded

//CLAM: P=0.00, the variable stays in the model

//AFAQ: P=0.00, the variable stays in the model

//PSS-14:P=0.44, the variable is excluded

//PSQI: P=0.46, the variable is excluded

//GAD-7: P=0.03, the variable stays in the model

*STEP 1. Full base Model

//Starting with a full Model and saves AIC and BIC. AIC/BIC is a reference point. If a reduced model has equal or lower AIC/BIC it is a better balance between

//fit and simplicity. If AIC/BIC increases when a variable is eliminated it probably contributed to the Model even though the p-value was low.

nbreg TLI_Alla_Segment_DL i.Age_cat DL_Nm3BWm_100 AFAQ GAD7, exposure(Exposure_Days_Md) vce(cluster Subject) irr

est store full_1_DL_noCAT

estat ic //AIC=235.5605 BIC=259.954, Wald chi2=16.55 and R2=0.0719

*STEP 2. Wald test and confounding check for each variable

*GAD-7

//a. Wald test:

//Eliminating and testing one variable at a time with the wald test (LR test is invalid for models with robust vce).

//If p<0.05 the variable contributes significantly and it should stay in the model.

//If p>0.05 the variable does not contribute to the model and could be eliminated.

est store full_1_DL_noCAT

testparm GAD7 //p=0.27

//b. Confounding check

//Even though a variable is non-significant, it could be a confounder and should therefore stay in the Model

//The rule of thumb is that a change of 10% or more of the IRR is a confounder (comparing the full Model with the reduced Model)

nbreg TLI_Alla_Segment_DL i.Age_cat DL_Nm3BWm_100 AFAQ, exposure(Exposure_Days_Md) vce(cluster Subject) irr //GAD-7 is taken away.

est store reduced_1_DL_noCAT

est table full_1_DL_noCAT reduced_1_DL_noCAT, eform b(%9.3f) se(%9.3f) stats(N ll) //no changes greater than 10% found

//CONCLUSION: GAD-7 is excluded from the model

*STEP 3. Interaction terms

//Chooses to test interaction for age x CLAM, age x AFAQ,

//If the interaction term is significant and/or gives a theoretical reasonable pattern the interaction terms stay in the Model.

//AIC and BIC in the model with interaction terms is compared to the model without interactions, if AIC decreases a lot and the interaction term is theoretical

//reasonable, the terms stay in the Model.

nbreg TLI_Alla_Segment_DL i.Age_cat DL_Nm3BWm_100 AFAQ, exposure(Exposure_Days_Md) vce(cluster Subject) irr //this is the new full model to compare with:

//AIC=234.5966 BIC=255.5054, wald chi2=15 and R2=0.067

//age x AFAQ

nbreg TLI_Alla_Segment_DL i.Age_cat##c.AFAQ DL_Nm3BWm_100 , exposure(Exposure_Days_Md) vce(cluster Subject) irr //No significant interaction

//AIC=235.4798, BIC=263.3582

//Age X CLAM

nbreg TLI_Alla_Segment_DL i.Age_cat##c.DL_Nm3BWm_100 AFAQ, exposure(Exposure_Days_Md) vce(cluster Subject) irr //for cat 20-24 there is a significant interaction

//AIC=233.8537, BIC=261.7321, Wald=29, R2=0.087-->AIC and R2 is a little bit better although BIC is worse

nbreg TLI_Alla_Segment_DL i.Age_cat##c.DL_Nm3BWm_100 AFAQ, exposure(Exposure_Days_Md) vce(cluster Subject)

testparm i.Age_cat#c.DL_Nm3BWm_100 //p=0.0468, the interaction i significant

//Conclusion: There is a significant interaction for Age and CLAM

******HOW TO INTERPRET THE INTERACTION TERM NUMERICALLY, 16-19 IS THE REFERENCE CATEGORY******

//IRR_DL_16-19=0.950

di 0.950*1.048 //IRR_DL_20-24=0.950*1.047=.9956

di 0.950*1.011 //IRR_DL_>24=0.950*1.011=0.96045

//lincom gives the IRR, p value and CI for the protective aspect of hip strength in each Age cat

nbreg TLI_Alla_Segment_DL i.Age_cat##c.DL_Nm3BWm_100 AFAQ, exposure(Exposure_Days_Md) vce(cluster Subject) irr

lincom DL_Nm3BWm_100, eform //IRR=0.95, p=0.95

lincom DL_Nm3BWm_100 + 2.Age_cat#c.DL_Nm3BWm_100, eform //IRR=0.995, P=0.71

lincom DL_Nm3BWm_100 + 3.Age_cat#c.DL_Nm3BWm_100, eform //IRR=0.96, P=0.008

//A plot on the linear relation

margins Age_cat, at(DL_Nm3BWm_100=(70(5)110))

marginsplot, x(DL_Nm3BWm_100) ylabel(, angle(0)) ytitle("Predicted injury rate") title("Interaction: Age × Hip Strength")

//"Strength was significantly protective in the youngest (15–19 years) and oldest (>24 years) groups, with IRRs of 0.95 (p = 0.002) and 0.96 (p = 0.008), respectively.

//In contrast, no meaningful association was observed in the 20–24-year group (IRR = 1.00, p = 0.71). Although the interaction between strength and the >24-year group

//was not statistically significant, lincom tests confirmed that the combined effect of strength within this age category remained statistically significant."

//CONCLUSION: There is a significant interaction for Age and CLAM

//This is the new full model

nbreg TLI_Alla_Segment_DL i.Age_cat##c.DL_Nm3BWm_100 AFAQ, exposure(Exposure_Days_Md) vce(cluster Subject) irr

//AIC=233.8537, BIC=261.7321, Wald=29, R2=0.087-->AIC and R2 is a little bit better although BIC is worse

*STEP 4. Model fit and diagnostic

//Pearson residuals >2 could be deviant and residuals >3 should definitely be scrutinized (is the Model sensitive to outliers)

//First a visual inspection should be done from scatters, secondly the outliers should be listed and scrutinized regarding their data (data input), thirdly a sensitivity

//analysis should be done to see if the conclusions hold up (model robustness) or not (discussion that the Model is affected by outliers)

//Alt 2: A more complete diagnostic where glm with the nbinom-family is used to receive Pearson residuals/deviance and influential statistics

//OBS! this is just for diagnostics and should not replace nbreg Modelling

//This is the new full model

nbreg TLI_Alla_Segment_DL i.Age_cat##c.DL_Nm3BWm_100 AFAQ, exposure(Exposure_Days_Md) vce(cluster Subject) irr

//AIC=233.8537, BIC=261.7321, Wald=29, R2=0.087

glm TLI_Alla_Segment_DL i.Age_cat##c.DL_Nm3BWm_100 AFAQ, family(nbinomial `a') link(log) exposure(Exposure_Days_Md) nolog

predict muhat_glm_DL_noCAT, mu

predict pearson_glm_DL_noCAT, pearson

predict devres_glm_DL_noCAT, deviance

predict hat_glm_DL_noCAT, hat

predict cook_glm_DL_noCAT, cooksd

scatter pearson_glm_DL_noCAT muhat_glm_DL_noCAT, yline(0) title("Pearson residuals (glm,DL_noCAT)") //There is a lot of observation >3

//But there are some outliers with residuals between 4 to 8.

list Subject muhat_glm_DL_noCAT pearson_glm_DL_noCAT if abs(pearson_glm_DL_noCAT) > 3 //Subject 262 have missing data.

//Subject 106,123,162, 37, 17, 157, 173, 32, 207, 107, 64, 136, 264, 63, 180 are outliers

list if Subject == 106 | Subject == 123| Subject == 37 | Subject == 17| Subject == 157| Subject == 173| Subject == 32| Subject == 207| Subject == 107 | Subject == 64| Subject == 136| Subject == 63| Subject == 264| Subject == 180

list Subject TLI_Alla_Segment_DL i.Age_cat##c.DL_Nm3BWm_100 AFAQ pearson_glm_DL_noCAT if Subject == 106 | Subject == 123| Subject == 37 | Subject == 17| Subject == 157| Subject == 173| Subject == 32| Subject == 207| Subject == 107 | Subject == 64| Subject == 136| Subject == 63| Subject == 264| Subject == 180

//207 have missing data

list Subject TLI_Alla_Segment_DL i.Age_cat DL_Nm3BWm_100 AFAQ pearson_glm_DL_noCAT if Subject == 106 | Subject == 123| Subject == 37 | Subject == 17| Subject == 157| Subject == 173| Subject == 32| Subject == 207| Subject == 107 | Subject == 64| Subject == 136| Subject == 63| Subject == 264| Subject == 180

//Conclusion:No data has been recorded incorrectly. //Subject 207 and 262 have missing data and are not outliers. //subject 106, 157, 107, 64 and 63 has values far over 3

nbreg TLI_Alla_Segment_DL i.Age_cat##c.DL_Nm3BWm_100 AFAQ, exposure(Exposure_Days_Md) vce(cluster Subject) irr //Exclusion of outliers should be done on the nbreg model with vce

nbreg TLI_Alla_Segment_DL i.Age_cat##c.DL_Nm3BWm_100 AFAQ if Subject!=106 & Subject!=123 & Subject!=162 & Subject!=37 & Subject!=17 & Subject!=157 & Subject!=173 & Subject!=32 & Subject!=207 & Subject!=107 & Subject!=64 & Subject!=136 & Subject!=63 & Subject!=264 & Subject!=180, exposure(Exposure_Days_Md) vce(cluster Subject) irr

//Elimination of all those subjects affects the model so that the interaction term for age cat 20-24 is n.s. No change in direction for any variable

nbreg TLI_Alla_Segment_DL i.Age_cat##c.DL_Nm3BWm_100 AFAQ if Subject!=106 & Subject!=157 & Subject!=63 & Subject!=107 & Subject!=64, exposure(Exposure_Days_Md) vce(cluster Subject) irr

//no greater change when eliminating those with the highest residuals

//Conclusion: OBS!! Excluding all outliers or just the outliers with highest residuals//does not affect the model regarding the direction of the IRR but in one case the p value//However, this change eliminates the significant difference between the age groups which is a positive change

//b. Analysis of deviance residuals

//Deviance residuals: Values greater than 2-3 could indicate deviance.

scatter devres_glm_DL_noCAT muhat_glm_DL_noCAT, yline(0) title("Deviance residuals (glm, DL_noCAT)")

//Residuals are in reasonable limits (<3), the curves have no systematic patterns, no signs of gross misspecification.

//c. Leverage graphs (hat values):

//How much an observation affects its prediction (if it is an influential point. Pearson and deviance residuals show if an observation

//is an outlier, but leverage shows if the outlier is an influential point, i.e. how much the outlier affect the model),

//2p/n is a simple cut off. in my case: di (2*7)/241=0.05809129. That will say: hat-values greater than 0.1–0.2 could be values to check.

scatter hat_glm_DL_noCAT muhat_glm_DL_noCAT, title("Leverage vs. Predicted values") ytitle("Leverage (hat_DL_noCAT)") xtitle("Predicted counts (muhat_DL_noCAT)")

// There are a lot of data points over 0.06

list Subject hat_glm_DL_noCAT muhat_glm_DL_noCAT if hat_glm_DL_noCAT > 0.03 //

gsort -hat_glm_DL_noCAT

list Subject hat_glm_DL_noCAT muhat_glm_DL_noCAT in 1/10 //The ten highest data points are: Subject 261, 80, 76, 186, 206, 12, 268, 43, 10, 188

nbreg TLI_Alla_Segment_DL i.Age_cat##c.DL_Nm3BWm_100 AFAQ, exposure(Exposure_Days_Md) vce(cluster Subject) irr //Full Model

//AIC=233.8537, BIC=261.7321, Wald=29, R2=0.087

nbreg TLI_Alla_Segment_DL i.Age_cat##c.DL_Nm3BWm_100 AFAQ if Subject!=261, exposure(Exposure_Days_Md) vce(cluster Subject) irr //no changes when the highest hat is excluded

nbreg TLI_Alla_Segment_DL i.Age_cat##c.DL_Nm3BWm_100 AFAQ if Subject!=80 & Subject!=76 & Subject!=261, exposure(Exposure_Days_Md) vce(cluster Subject) irr

//no changes when the three highest hat is excluded

nbreg TLI_Alla_Segment_DL i.Age_cat##c.DL_Nm3BWm_100 AFAQ if Subject!=80 & Subject!=261 & Subject!=76 & Subject!=12 & Subject!=186 & Subject!=206 & Subject!=268 & Subject!=43 & Subject!=10 & Subject!=188, exposure(Exposure_Days_Md) vce(cluster Subject) irr

//AIC=214.0287 , 241.568, Wald=17.6, R2=0.07. Exclusion of all those variables, changes the significance of hip strength to p=0.054

//and the interaction terms will be n.s. Furthermore, the direction of the category >24 will change direction but are still very close to 1.0

//i.e. the message that there is no greater difference regarding the protective effect of hip strength in the different age categories still stands.

//however, the effect of hip strength will change from significant to n.s

//Conclusion: exclusion of all influential points does not change the direction of hip strength but its significance. //However, the ten highest influential points have to be excluded to see this change so no solitary influential point exists.

//d. Cook´s distance:

//High estimates of pearson/deviance residuals and high estimates of leverage will give a high cooks d.

//Cooks d tells you how much your model would be affected if one observation was taken away.

//Cook’s D has no definitive cut off. A rule of thumb is that values >1.0 in greater data sets are problematic.

//In smaller data sets as this values 0.05-0.1 could be troublesome and values >0.1 should absolutely be scrutinized.

//Another conservative rule is 4/n. In my case n ≈ 241 → 4/241 ≈ 0.017. so, for this material values from 0.02 could be of interest

scatter cook_glm_DL_noCAT muhat_glm_DL_noCAT, title("Cook's distance") // a lot of subjects over 0.017

sort cook_glm_DL_noCAT

list Subject TLI_Alla_Segment_DL Age DL_Nm3BWm_100 AFAQ Exposure_Days_Md if _n > _N-5 //Gives the 5 highest cooks d values

//Subject 162, 207 and 262 has missing data and are not outliers. 63 och 242 has the highest cooks D.

nbreg TLI_Alla_Segment_DL i.Age_cat##c.DL_Nm3BWm_100 AFAQ if Subject!=63 & Subject!=242, exposure(Exposure_Days_Md) vce(cluster Subject) irr

//Exclusion of subject 63 and 242 does not change the model

nbreg TLI_Alla_Segment_DL i.Age_cat##c.DL_Nm3BWm_100 AFAQ if Subject!=63 & Subject!=242& Subject!=162 & Subject!=207 & Subject!=262, exposure(Exposure_Days_Md) vce(cluster Subject) irr

//Exclusion of subject the five highest Cooks D does not change the model

list Subject muhat_glm_DL_noCAT hat_glm_DL_noCAT devres_glm_DL_noCAT pearson_glm_DL_noCAT cook_glm_DL_noCAT if hat_glm_DL_noCAT > 0.03 | abs(devres_glm_DL_noCAT) > 3 | abs(pearson_glm_DL_noCAT) > 3 | cook_glm_DL_noCAT > 0.02

//no cook´s D over 0.07 which is good. Does not see anything that could affect the model

//CONCLUSION: The model is not in a significant way affected by any outliers, residuals or leverage points //both regarding direction (robustness) and precision (p value)

//c. Specification test-Linktest

//The linktest could indicate that the model is not an appropriate representation of the relationship between the outcome and predictors (misspecification), and furthermore that

//important predictors have been omitted or are represented incorrectly in the model. Hatsq should be n.s and hat should be significant. However, In the literature it is

//mentioned that _hat should be significant since it is the predicted value for the model. In a negative binomial regression Model the pseudo R2 most often are very low

//(0.05-0.10), see STATA manual or Hilbe 2011. Hilbe discuss the general weak predictive value in NB-models, especially with overdispersion and many zeroes.

nbreg TLI_Alla_Segment_DL i.Age_cat##c.DL_Nm3BWm_100 AFAQ, exposure(Exposure_Days_Md) vce(cluster Subject) irr

linktest //Conclusion:_hatsq=non-significant. This indicates that the model is correctly specified and are the most important finding from this test.

//furthermore, _hat is non-significant p=0.95.

//AFAQ HAS A HIGH IRR, 8% INCREASE FOR EACH UNIT, IS THE RELATIONSHIP TO THE OUTCOME REALLY LINEAR?

//Testing the linearity with the outcome TLI

nbreg TLI_Alla_Segment_DL c.AFAQ c.AFAQ#c.AFAQ, exposure(Exposure_Days_Md) vce(cluster Subject)

//the interaction term is n.s. If the interaction term is n.s the relation is probably linear

nbreg TLI_Alla_Segment_DL AFAQ, exposure(Exposure_Days_Md) vce(cluster Subject) irr

margins, at(AFAQ=(0(5)45)) predict(ir)

marginsplot, x(AFAQ) ytitle("Predicted incidence rate (IR)") xtitle("AFAQ score") title("Predicted injury rate by AFAQ") ylabel(, angle(horizontal))

//margins and marginsplot suggest a curvilinear relationship

//Testing the linearity for AFAQ in the full model

nbreg TLI_Alla_Segment_DL i.Age_cat##c.DL_Nm3BWm_100 c.AFAQ c.AFAQ#c.AFAQ, exposure(Exposure_Days_Md) vce(cluster Subject)

//The interaction term is significant p=0.048, suggesting a significant quadratic effect and a curvilinear relationship

nbreg TLI_Alla_Segment_DL i.Age_cat##c.DL_Nm3BWm_100 AFAQ, exposure(Exposure_Days_Md) vce(cluster Subject) irr

margins, at(AFAQ=(0(5)45)) predict(n)

marginsplot, x(AFAQ) ytitle("Predicted number of time-loss injuries") xtitle("AFAQ score") title("Predicted number of time-loss injuries by AFAQ") ylabel(, angle(horizontal))

//margins and marginsplot suggest a curvilinear relationship

nbreg TLI_Alla_Segment_DL i.Age_cat##c.DL_Nm3BWm_100 AFAQ, exposure(Exposure_Days_Md) vce(cluster Subject) irr

margins, at(AFAQ=(0(1)45)) predict(n)

marginsplot, ytitle("Predicted injuries (n)") xtitle("AFAQ score") title("Predicted injuries by AFAQ (full model)") ylabel(, angle(horizontal))

//margins and marginsplot suggest a curvilinear relationship

//An alternative for figure 1 with a 95% confidence band across the full observed AFAQ range

nbreg TLI_Alla_Segment_DL i.Age_cat##c.DL_Nm3BWm_100 AFAQ, exposure(Exposure_Days_Md) vce(cluster Subject) irr

margins, at(AFAQ=(10(1)45)) predict(n)

marginsplot, x(AFAQ) recast(line) recastci(rarea) ytitle("Predicted number of time-loss injuries") xtitle("AFAQ score") ylabel(, angle(horizontal))

//"In the full model, including age, hip strength, and AFAQ, marginal effects showed that the risk of injury increased with higher AFAQ scores.

//Adding a quadratic term for AFAQ revealed a significant non-linear effect (p = 0.048), indicating that the increase in risk was more pronounced at higher scores.

*THIS IS THE FINAL MODEL FOR TLI FOR THE DOMINANT

nbreg TLI_Alla_Segment_DL i.Age_cat##c.DL_Nm3BWm_100 AFAQ, exposure(Exposure_Days_Md) vce(cluster Subject) irr //Full Model

//AIC=233.8537, BIC=261.7321, Wald=29, R2=0.087

//OBS!! This model is robust both regarding direction and precision (p-value). However, "In the full model, including age, hip strength, and AFAQ, marginal effects showed that

//the risk of injury increased with higher AFAQ scores. Adding a quadratic term for AFAQ revealed a significant non-linear effect (p = 0.048), indicating that the increase

****
* Mean centering of hip strength for Table 4
****

sum DL_Nm3BWm_100, detail
gen DL_strength_c = DL_Nm3BWm_100 - 97.05113
nbreg TLI_Alla_Segment_DL i.Age_cat##c.DL_strength_c AFAQ, exposure(Exposure_Days_Md) vce(cluster Subject) irr

*MODEL BUILDING OVERUSE INJURY: CONTROL OF ASSUMPTIONS FOR BOTH NDL AND DL

* STEP 1. DESKRIPTIV ANALYSIS

//Evaluating the covariates for outliers, missing data (most of this is done in study 4 for those variables so this is mostly a quick check) and its relation to the

//outcome variable

//The variable div

poisson Total_OSTRC_Alla_Segm_NDL_Counts div, exposure (Exposure_Days_Md) irr

poisson Total_OSTRC_Alla_Segm_NDL_Counts i.div, exposure (Exposure_Days_Md) irr //n.s

tab div

tab Total_OSTRC_Alla_Segm_NDL_Counts div // a lot of zeroes

poisson Total_OSTRC_Alla_Segm_DL_Counts div, exposure (Exposure_Days_Md) irr //p=0.02***

poisson Total_OSTRC_Alla_Segm_DL_Counts i.div, exposure (Exposure_Days_Md) irr //P=0.02 in div 3

tab div

tab Total_OSTRC_Alla_Segm_DL_Counts div // a lot of zeroes

//The variable Age

poisson Total_OSTRC_Alla_Segm_NDL_Counts Age, exposure (Exposure_Days_Md) irr //p=0.017***

sum Age, detail

graph box Age //Two outliers

scatter Age Subject, mlabel(Subject) //Subjects 76 and 143 is the outliers

poisson Total_OSTRC_Alla_Segm_NDL_Counts Age, exposure (Exposure_Days_Md) irr

poisson Total_OSTRC_Alla_Segm_NDL_Counts Age if Subject!=143 & Subject !=76, exposure (Exposure_Days_Md) irr // Conclusion: No significant change in IRR, CI, Std error or P

poisson Total_OSTRC_Alla_Segm_DL_Counts Age, exposure (Exposure_Days_Md) irr //p=0.002***

sum Age, detail

graph box Age //Two outliers

scatter Age Subject, mlabel(Subject) //Subjects 76 and 143 is the outliers

poisson Total_OSTRC_Alla_Segm_DL_Counts Age, exposure (Exposure_Days_Md) irr

poisson Total_OSTRC_Alla_Segm_DL_Counts Age if Subject!=143 & Subject !=76, exposure (Exposure_Days_Md) irr // Conclusion: No significant change in IRR, CI, Std error or P

//The variable Weightkg

poisson Total_OSTRC_Alla_Segm_NDL_Counts Weightkg, exposure (Exposure_Days_Md) irr //p=0.16

sum Weightkg, detail

graph box Weightkg //three outliers

scatter Weightkg Subject, mlabel (Subject) //subject 34, 195 and 231 are outliers

poisson Total_OSTRC_Alla_Segm_NDL_Counts Weightkg, exposure (Exposure_Days_Md) irr

poisson Total_OSTRC_Alla_Segm_NDL_Counts Weightkg if Subject!=34 & Subject !=195 & Subject !=231, exposure (Exposure_Days_Md) irr

// Conclusion: No significant change in IRR, CI, Std error or P

poisson Total_OSTRC_Alla_Segm_DL_Counts Weightkg, exposure (Exposure_Days_Md) irr // p=0.77

sum Weightkg, detail

graph box Weightkg //three outliers

scatter Weightkg Subject, mlabel (Subject) //subject 34, 195 and 231 are outliers

poisson Total_OSTRC_Alla_Segm_DL_Counts Weightkg, exposure (Exposure_Days_Md) irr

poisson Total_OSTRC_Alla_Segm_DL_Counts Weightkg if Subject!=34 & Subject !=195 & Subject !=231, exposure (Exposure_Days_Md) irr

// Conclusion: No significant change in P value but the IRR goes from 0.99 to 1.00

//The variable Lengthm

poisson Total_OSTRC_Alla_Segm_NDL_Counts Lengthm, exposure (Exposure_Days_Md) irr //p=0.14

sum Lengthm, detail

graph box Lengthm //One outlier

scatter Lengthm Subject, mlabel (Subject) //subject 88 is an outlier

poisson Total_OSTRC_Alla_Segm_NDL_Counts Lengthm, exposure (Exposure_Days_Md) irr

poisson Total_OSTRC_Alla_Segm_NDL_Counts Lengthm if Subject!=88, exposure (Exposure_Days_Md) irr

// Conclusion: The P value is getting closer to 0.05 and IRR changes from 0.35 to 0.25

poisson Total_OSTRC_Alla_Segm_DL_Counts Lengthm, exposure (Exposure_Days_Md) irr //p=0.072

sum Lengthm, detail

graph box Lengthm //One outlier

scatter Lengthm Subject, mlabel (Subject) //subject 88 is an outlier

poisson Total_OSTRC_Alla_Segm_DL_Counts Lengthm, exposure (Exposure_Days_Md) irr

poisson Total_OSTRC_Alla_Segm_DL_Counts Lengthm if Subject!=88, exposure (Exposure_Days_Md) irr // Conclusion: The P values get significant och IRR drops a little bit

//The variable BMI

poisson Total_OSTRC_Alla_Segm_NDL_Counts BMI, exposure (Exposure_Days_Md) irr //p=0.66

sum BMI, detail

graph box BMI

scatter BMI Subject, mlabel (Subject) //Subject 34 i an outlier

list Subject BMI

poisson Total_OSTRC_Alla_Segm_NDL_Counts BMI if Subject !=34, exposure (Exposure_Days_Md) irr // Conclusion: No significant change in OR, CI, Std error or P

poisson Total_OSTRC_Alla_Segm_DL_Counts BMI, exposure (Exposure_Days_Md) irr //p=0.22

sum BMI, detail

graph box BMI

scatter BMI Subject, mlabel (Subject) //Subject 34 i an outlier

list Subject BMI

poisson Total_OSTRC_Alla_Segm_DL_Counts BMI if Subject !=34, exposure (Exposure_Days_Md) irr // Conclusion: No significant change in OR, CI, Std error or P

//The variable SLS/passfail

poisson Total_OSTRC_Alla_Segm_NDL_Counts NDL_SLSfailpass, exposure (Exposure_Days_Md) irr //p=0.01***

tab NDL_SLSfailpass

tab NDL_SLSfailpass Total_OSTRC_Alla_Segm_NDL_Counts

poisson Total_OSTRC_Alla_Segm_DL_Counts DL_SLSfailpass, exposure (Exposure_Days_Md) irr //p=0.006

tab DL_SLSfailpass

tab DL_SLSfailpass Total_OSTRC_Alla_Segm_DL_Counts

//The variable ADF

poisson Total_OSTRC_Alla_Segm_NDL_Counts NDL_ADF, exposure (Exposure_Days_Md) irr //p=0.21

sum NDL_ADF, detail

graph box NDL_ADF //Two outliers

scatter NDL_ADF Subject, mlabel (Subject) //Subject 109 and 251 is the outliers

list Subject NDL_ADF

poisson Total_OSTRC_Alla_Segm_NDL_Counts NDL_ADF if Subject !=109 & Subject !=251, exposure (Exposure_Days_Md) irr //No significant change in OR, CI, Std error or P

poisson Total_OSTRC_Alla_Segm_DL_Counts DL_ADF, exposure (Exposure_Days_Md) irr //p=0.0001

sum DL_ADF, detail

graph box DL_ADF //Three outliers

scatter DL_ADF Subject, mlabel (Subject) //Subject 109, 258 and 6 is the outliers

list Subject DL_ADF

poisson Total_OSTRC_Alla_Segm_DL_Counts DL_ADF if Subject !=109 & Subject !=258 & Subject !=6, exposure (Exposure_Days_Md) irr //A small change in IRR

//The variable CLAM

poisson Total_OSTRC_Alla_Segm_NDL_Counts NDL_Nm3BWm_100, exposure (Exposure_Days_Md) irr //p=0.8

sum NDL_Nm3BWm_100, detail

graph box NDL_Nm3BWm_100 //Three outliers

scatter NDL_Nm3BWm_100 Subject, mlabel (Subject) //Subject 261, 90, 41 is the outliers

list Subject NDL_Nm3BWm_100

poisson Total_OSTRC_Alla_Segm_NDL_Counts NDL_Nm3BWm_100 if Subject !=261 & Subject !=90 & Subject !=41, exposure (Exposure_Days_Md) irr //No significant change in OR, CI, Std error or P

poisson Total_OSTRC_Alla_Segm_DL_Counts DL_Nm3BWm_100, exposure (Exposure_Days_Md) irr //p=0.17

sum DL_Nm3BWm_100, detail

graph box DL_Nm3BWm_100 //four outliers

scatter DL_Nm3BWm_100 Subject, mlabel (Subject) //Subject 261, 245, 226 and 90 is the outliers

list Subject DL_Nm3BWm_100

poisson Total_OSTRC_Alla_Segm_DL_Counts DL_Nm3BWm_100 if Subject !=261 & Subject !=245 & Subject !=226 & Subject !=90, exposure (Exposure_Days_Md) irr //No significant change in OR, CI, Std error or P

//The variable AFAQ

poisson Total_OSTRC_Alla_Segm_NDL_Counts AFAQ, exposure (Exposure_Days_Md) irr //p=0.0000***

sum AFAQ, detail

graph box AFAQ //Three outliers

scatter AFAQ Subject, mlabel (Subject) //Subject 80 and 55 and 43 is the outliers

list Subject AFAQ

poisson Total_OSTRC_Alla_Segm_NDL_Counts AFAQ if Subject !=80 & Subject !=55 & Subject !=43, exposure (Exposure_Days_Md) irr //No changes

poisson Total_OSTRC_Alla_Segm_DL_Counts AFAQ, exposure (Exposure_Days_Md) irr //p=0.00***

sum AFAQ, detail

graph box AFAQ //three outliers

scatter AFAQ Subject, mlabel (Subject) //Subject 80 and 55 and 43 is the outliers

list Subject AFAQ

poisson Total_OSTRC_Alla_Segm_DL_Counts AFAQ if Subject !=80 & Subject !=55 & Subject !=43, exposure (Exposure_Days_Md) irr //No changes

//The variable PSS14

poisson Total_OSTRC_Alla_Segm_NDL_Counts PSS14, exposure (Exposure_Days_Md) irr //p=0.15

sum PSS14, detail

graph box PSS14 //Tw0 outliers

scatter PSS14 Subject, mlabel (Subject) //Subject 46 and 189 is the outliers

list Subject PSS14

poisson Total_OSTRC_Alla_Segm_NDL_Counts PSS14 if Subject !=46 & Subject !=189, exposure (Exposure_Days_Md) irr //No change

poisson Total_OSTRC_Alla_Segm_DL_Counts PSS14, exposure (Exposure_Days_Md) irr //p=0.04***

sum PSS14, detail

graph box PSS14 //three outliers

scatter PSS14 Subject, mlabel (Subject) //Subject 46 and 189 is the outliers

list Subject PSS14

poisson Total_OSTRC_Alla_Segm_DL_Counts PSS14 if Subject !=46 & Subject !=189, exposure (Exposure_Days_Md) irr //no change

//The variable PSQI

poisson Total_OSTRC_Alla_Segm_NDL_Counts PSQI_cat2, exposure (Exposure_Days_Md) irr //p=0.14

sum PSQI, detail

graph box PSQI //Two outliers

scatter PSQI Subject, mlabel (Subject) //Subject 225 and 92 is the outliers

list Subject PSQI

tab PSQI_cat2 Total_OSTRC_Alla_Segm_NDL_Counts // a lot of zeroes for higher values of OSTRC

poisson Total_OSTRC_Alla_Segm_NDL_Counts PSQI_cat2 if Subject !=92 & Subject !=225, exposure (Exposure_Days_Md) irr //No change

poisson Total_OSTRC_Alla_Segm_DL_Counts PSS14, exposure (Exposure_Days_Md) irr //p=0.04***

sum PSS14, detail

graph box PSS14 //two outliers

scatter PSS14 Subject, mlabel (Subject) //Subject 225 and 92 is the outliers

list Subject PSS14

tab PSQI_cat2 Total_OSTRC_Alla_Segm_DL_Counts // a lot of zeroes for higher values of OSTRC

poisson Total_OSTRC_Alla_Segm_DL_Counts PSS14 if Subject !=92 & Subject !=225, exposure (Exposure_Days_Md) irr //no change

//The variable GAD-7

poisson Total_OSTRC_Alla_Segm_NDL_Counts GAD7, exposure (Exposure_Days_Md) irr //p=0.01***

sum GAD7, detail

graph box GAD7 //four outliers

scatter GAD7 Subject, mlabel (Subject) //Subject 45, 31, 7 and 230 are the outliers

list Subject GAD7

tab GAD7_cat2 Total_OSTRC_Alla_Segm_NDL_Counts // a lot of zeroes

poisson Total_OSTRC_Alla_Segm_NDL_Counts GAD7 if Subject !=7 & Subject !=31 & Subject !=45 & Subject !=230, exposure (Exposure_Days_Md) irr //No changes

poisson Total_OSTRC_Alla_Segm_DL_Counts GAD7, exposure (Exposure_Days_Md) irr //p=0.02

sum GAD7, detail

graph box GAD7//four outliers

scatter GAD7 Subject, mlabel (Subject) //Subject 45, 31, 7 and 230 are the outliers

list Subject GAD7

tab GAD7_cat2 Total_OSTRC_Alla_Segm_DL_Counts // a lot of zeroes

poisson Total_OSTRC_Alla_Segm_DL_Counts GAD7 if Subject !=7 & Subject !=31 & Subject !=45 & Subject !=230, exposure (Exposure_Days_Md) irr //no changes

//OVERALL CONCLUSION: no greater outliers due to wrongly registered data or missing data///////////////////////////

* STEP 2 EVALUATING THE OUTCOME VARAIALBE FOR ZERO INFLATION AND THE OFFSET VARIABLE

//Evaluating the outcome variable and exposure (offset variable).

//A.Exposure

sum Exposure_Days_Min, detail //mean 237, max 300, min 30

sum Exposure_Days_Md, detail //mean 228, max 300, min 30

sum Exposure_Days_Max, detail //mean 219, max 300, min 27

poisson Total_OSTRC_Alla_Segm_NDL_Counts BMI, exposure (Exposure_Days_Min) irr

poisson Total_OSTRC_Alla_Segm_NDL_Counts BMI, exposure (Exposure_Days_Md) irr

poisson Total_OSTRC_Alla_Segm_NDL_Counts BMI, exposure (Exposure_Days_Max) irr

poisson Total_OSTRC_Alla_Segm_NDL_Counts Age, exposure (Exposure_Days_Min) irr

poisson Total_OSTRC_Alla_Segm_NDL_Counts Age, exposure (Exposure_Days_Md) irr

poisson Total_OSTRC_Alla_Segm_NDL_Counts Age, exposure (Exposure_Days_Max) irr

poisson Total_OSTRC_Alla_Segm_NDL_Counts NDL_SLSfailpass, exposure (Exposure_Days_Min) irr

poisson Total_OSTRC_Alla_Segm_NDL_Counts NDL_SLSfailpass, exposure (Exposure_Days_Md) irr

poisson Total_OSTRC_Alla_Segm_NDL_Counts NDL_SLSfailpass, exposure (Exposure_Days_Max) irr

poisson Total_OSTRC_Alla_Segm_NDL_Counts NDL_ADF, exposure (Exposure_Days_Min) irr

poisson Total_OSTRC_Alla_Segm_NDL_Counts NDL_ADF, exposure (Exposure_Days_Md) irr

poisson Total_OSTRC_Alla_Segm_NDL_Counts NDL_ADF, exposure (Exposure_Days_Max) irr

poisson Total_OSTRC_Alla_Segm_NDL_Counts NDL_Nm3BWm_100, exposure (Exposure_Days_Min) irr

poisson Total_OSTRC_Alla_Segm_NDL_Counts NDL_Nm3BWm_100, exposure (Exposure_Days_Md) irr

poisson Total_OSTRC_Alla_Segm_NDL_Counts NDL_Nm3BWm_100, exposure (Exposure_Days_Max) irr

poisson Total_OSTRC_Alla_Segm_NDL_Counts AFAQ, exposure (Exposure_Days_Min) irr

poisson Total_OSTRC_Alla_Segm_NDL_Counts AFAQ, exposure (Exposure_Days_Md) irr

poisson Total_OSTRC_Alla_Segm_NDL_Counts AFAQ, exposure (Exposure_Days_Max) irr

poisson Total_OSTRC_Alla_Segm_NDL_Counts PSS14, exposure (Exposure_Days_Min) irr

poisson Total_OSTRC_Alla_Segm_NDL_Counts PSS14, exposure (Exposure_Days_Md) irr

poisson Total_OSTRC_Alla_Segm_NDL_Counts PSS14, exposure (Exposure_Days_Max) irr

poisson Total_OSTRC_Alla_Segm_NDL_Counts PSQI, exposure (Exposure_Days_Min) irr

poisson Total_OSTRC_Alla_Segm_NDL_Counts PSQI, exposure (Exposure_Days_Md) irr

poisson Total_OSTRC_Alla_Segm_NDL_Counts PSQI, exposure (Exposure_Days_Max) irr

poisson Total_OSTRC_Alla_Segm_NDL_Counts GAD7, exposure (Exposure_Days_Min) irr

poisson Total_OSTRC_Alla_Segm_NDL_Counts GAD7, exposure (Exposure_Days_Md) irr

poisson Total_OSTRC_Alla_Segm_NDL_Counts GAD7, exposure (Exposure_Days_Max) irr

poisson Total_OSTRC_Alla_Segm_DL_Counts BMI, exposure (Exposure_Days_Min) irr

poisson Total_OSTRC_Alla_Segm_DL_Counts BMI, exposure (Exposure_Days_Md) irr

poisson Total_OSTRC_Alla_Segm_DL_Counts BMI, exposure (Exposure_Days_Max) irr

poisson Total_OSTRC_Alla_Segm_DL_Counts Age, exposure (Exposure_Days_Min) irr

poisson Total_OSTRC_Alla_Segm_DL_Counts Age, exposure (Exposure_Days_Md) irr

poisson Total_OSTRC_Alla_Segm_DL_Counts Age, exposure (Exposure_Days_Max) irr

poisson Total_OSTRC_Alla_Segm_DL_Counts DL_SLSfailpass, exposure (Exposure_Days_Min) irr //p=0.07

poisson Total_OSTRC_Alla_Segm_DL_Counts DL_SLSfailpass, exposure (Exposure_Days_Md) irr //p=0.06

poisson Total_OSTRC_Alla_Segm_DL_Counts DL_SLSfailpass, exposure (Exposure_Days_Max) irr //p=0.047

poisson Total_OSTRC_Alla_Segm_DL_Counts DL_ADF, exposure (Exposure_Days_Min) irr

poisson Total_OSTRC_Alla_Segm_DL_Counts DL_ADF, exposure (Exposure_Days_Md) irr

poisson Total_OSTRC_Alla_Segm_DL_Counts DL_ADF, exposure (Exposure_Days_Max) irr

poisson Total_OSTRC_Alla_Segm_DL_Counts DL_Nm3BWm_100, exposure (Exposure_Days_Min) irr

poisson Total_OSTRC_Alla_Segm_DL_Counts DL_Nm3BWm_100, exposure (Exposure_Days_Md) irr

poisson Total_OSTRC_Alla_Segm_DL_Counts DL_Nm3BWm_100, exposure (Exposure_Days_Max) irr

poisson Total_OSTRC_Alla_Segm_DL_Counts AFAQ, exposure (Exposure_Days_Min) irr

poisson Total_OSTRC_Alla_Segm_DL_Counts AFAQ, exposure (Exposure_Days_Md) irr

poisson Total_OSTRC_Alla_Segm_DL_Counts AFAQ, exposure (Exposure_Days_Max) irr

poisson Total_OSTRC_Alla_Segm_DL_Counts PSS14, exposure (Exposure_Days_Min) irr

poisson Total_OSTRC_Alla_Segm_DL_Counts PSS14, exposure (Exposure_Days_Md) irr

poisson Total_OSTRC_Alla_Segm_DL_Counts PSS14, exposure (Exposure_Days_Max) irr

poisson Total_OSTRC_Alla_Segm_DL_Counts PSQI, exposure (Exposure_Days_Min) irr

poisson Total_OSTRC_Alla_Segm_DL_Counts PSQI, exposure (Exposure_Days_Md) irr

poisson Total_OSTRC_Alla_Segm_DL_Counts PSQI, exposure (Exposure_Days_Max) irr

poisson Total_OSTRC_Alla_Segm_DL_Counts GAD7, exposure (Exposure_Days_Min) irr

poisson Total_OSTRC_Alla_Segm_DL_Counts GAD7, exposure (Exposure_Days_Md) irr

poisson Total_OSTRC_Alla_Segm_DL_Counts GAD7, exposure (Exposure_Days_Max) irr

//OVERALL CONCLUSION for the offset variable: the use of different exposure does not change //the relationship with the outcome variable for each individual covariates (except for SLS passfail) therefore //Exposure_Days_Md will be used

//B.The outcome variable and zero inflation

tab Total_OSTRC_Alla_Segm_NDL_Counts

tab Total_OSTRC_Alla_Segm_DL_Counts //In relation to subjects with injuries both DL and NDL do not have to many zeroes=people that did not get any injury

//OVERALL CONCLUSION for zero inflation: There is no theoretical risk for zero inflation and //structural zeros in this data set.(structural zeros=the subject cannot get an injury due to some reason. //Theoretical risk that a subject cannot get injured= e.g. the player did not get so much time //on the field during the season)///

*STEP 3. CHECKING FOR THE LINEARITY ON THE LOG SCALE

//Evaluating whether the relationship (of each continuous variable) with the outcome variable is linear on the log scale

//OBS! with the command lowess the outcome variable should be evaluated in the log scale and if the curve is approximately straight there is a linear relationship.

//regarding the polynomial test (inclusion of a quadratic term) the quadratic term should not be significant for a relationship between the outcome variable

//and the covariates (Not on the log scale)

//The variable Age

poisson Total_OSTRC_Alla_Segm_NDL_Counts Age, exposure(Exposure_Days_Md) //OBS! no IRR as the test is done on the log scale

predict log_mu, xb

lowess log_mu Age, mean //the curve is not fully straight from 16 to 20 years.

twoway (scatter log_mu Age, msymbol(o))(lowess log_mu Age, bwidth(0.8)),title("lowess b=0.8") //more obvious that the variable is not linear

drop log_mu

```
poisson Total_OSTRC_Alla_Segm_DL_Counts Age, exposure(Exposure_Days_Md)

predict log_mu, xb

lowess log_mu Age, mean //the curve is not fully straight from 16 to 20 years.

twoway (scatter log_mu Age, msymbol(o))(lowess log_mu Age, bwidth(0.8)),title("lowess b=0.8") //more obvious that the variable is not linear

drop log_mu


gen Age2 = Age^2

poisson Total_OSTRC_Alla_Segm_NDL_Counts Age Age2, exposure(Exposure_Days_Md) irr // p=0.002***

poisson Total_OSTRC_Alla_Segm_DL_Counts Age Age2, exposure(Exposure_Days_Md) irr // n.s

drop Age2


poisson Total_OSTRC_Alla_Segm_NDL_Counts Age, exposure(Exposure_Days_Md)

est store lin

poisson Total_OSTRC_Alla_Segm_NDL_Counts Age Age2, exposure(Exposure_Days_Md)

est store quad

lrtest lin quad //p=0.006***

drop Age2


poisson Total_OSTRC_Alla_Segm_DL_Counts Age, exposure(Exposure_Days_Md)

est store lin

poisson Total_OSTRC_Alla_Segm_DL_Counts Age Age2, exposure(Exposure_Days_Md)

est store quad

lrtest lin quad //P=0.17

drop Age2


//Conclusion: Age is not linear for the NDL but for the DL
```

```
//The variable Weightkg

poisson Total_OSTRC_Alla_Segm_NDL_Counts Weightkg, exposure(Exposure_Days_Md)

predict log_mu, xb

lowess log_mu Weightkg, mean //the curve is linear

twoway (scatter log_mu Weightkg, msymbol(o))(lowess log_mu Weightkg,
bwidth(0.8)),title("lowess b=0.8") //The variable is not linear with low weight

drop log_mu


poisson Total_OSTRC_Alla_Segm_DL_Counts Weightkg, exposure(Exposure_Days_Md)

predict log_mu, xb

lowess log_mu Weightkg, mean //the curve is linear

twoway (scatter log_mu Weightkg, msymbol(o))(lowess log_mu Weightkg,
bwidth(0.8)),title("lowess b=0.8") //About the same as for the NDL

drop log_mu

gen Weightkg2 = Weightkg^2

poisson Total_OSTRC_Alla_Segm_NDL_Counts Weightkg Weightkg2,
exposure(Exposure_Days_Md) irr //p=0.00***

poisson Total_OSTRC_Alla_Segm_DL_Counts Weightkg Weightkg2,
exposure(Exposure_Days_Md) irr // p=0.00***

drop Weightkg2


poisson Total_OSTRC_Alla_Segm_NDL_Counts Weightkg, exposure(Exposure_Days_Md)

est store lin

poisson Total_OSTRC_Alla_Segm_NDL_Counts Weightkg Weightkg2,
exposure(Exposure_Days_Md)

est store quad

lrtest lin quad //P=0.00000

drop Weightkg2


poisson Total_OSTRC_Alla_Segm_DL_Counts Weightkg, exposure(Exposure_Days_Md)

est store lin

poisson Total_OSTRC_Alla_Segm_DL_Counts Weightkg Weightkg2,
exposure(Exposure_Days_Md)

est store quad
```

lrtest lin quad //P=0.00000

drop Weightkg2

//Conclusion: Weight is not linear for NDL and DL

//The variable Lengthm

poisson Total_OSTRC_Alla_Segm_NDL_Counts Lengthm, exposure(Exposure_Days_Md)

predict log_mu, xb

lowess log_mu Lengthm, mean //the curve is relatively straight

twoway (scatter log_mu Lengthm, msymbol(o))(lowess log_mu Lengthm, bwidth(0.8)),title("lowess b=0.8") //there is a plateau in the middle

drop log_mu

poisson Total_OSTRC_Alla_Segm_DL_Counts Lengthm, exposure(Exposure_Days_Md)

predict log_mu, xb

lowess log_mu Lengthm, mean //the curve is relatively straight

twoway (scatter log_mu Lengthm, msymbol(o))(lowess log_mu Lengthm, bwidth(0.8)),title("lowess b=0.8") //there is a plateau in the middle

drop log_mu

gen Lengthm2 = Lengthm^2

poisson Total_OSTRC_Alla_Segm_NDL_Counts Lengthm Lengthm2, exposure(Exposure_Days_Md) irr // p=0.04***

poisson Total_OSTRC_Alla_Segm_DL_Counts Lengthm Lengthm2, exposure(Exposure_Days_Md) irr // n.s

drop Lengthm2

poisson Total_OSTRC_Alla_Segm_NDL_Counts Lengthm, exposure(Exposure_Days_Md)

est store lin

poisson Total_OSTRC_Alla_Segm_NDL_Counts Lengthm Lengthm2, exposure(Exposure_Days_Md)

est store quad

lrtest lin quad //P=0.03****

drop Weightkg2

```
poisson Total_OSTRC_Alla_Segm_DL_Counts Lengthm, exposure(Exposure_Days_Md)

est store lin

poisson Total_OSTRC_Alla_Segm_DL_Counts Lengthm Lengthm2, exposure(Exposure_Days_Md)

est store quad

lrtest lin quad //P=0.066

drop Weightkg2

//Conclusion: Length is not linear for the NDL but for the DL



//The variable BMI

poisson Total_OSTRC_Alla_Segm_NDL_Counts BMI, exposure(Exposure_Days_Md)

predict log_mu, xb

lowess log_mu BMI, mean //the curve is relatively straight

twoway (scatter log_mu BMI, msymbol(o))(lowess log_mu BMI, bwidth(0.8)),title("lowess b=0.8") // the curve is not linear for small BMI

drop log_mu


poisson Total_OSTRC_Alla_Segm_DL_Counts BMI, exposure(Exposure_Days_Md)

predict log_mu, xb

lowess log_mu BMI, mean //the curve is relatively straight

twoway (scatter log_mu BMI, msymbol(o))(lowess log_mu BMI, bwidth(0.8)),title("lowess b=0.8")

// the curve is not linear for small BMI and have a reversed direction for those with higher BMI

drop log_mu


gen BMI2 = BMI^2

poisson Total_OSTRC_Alla_Segm_NDL_Counts BMI BMI2, exposure(Exposure_Days_Md) irr // p=0.00***

poisson Total_OSTRC_Alla_Segm_DL_Counts BMI BMI2, exposure(Exposure_Days_Md) irr // p=0.00***

drop BMI2
```

```
poisson Total_OSTRC_Alla_Segm_NDL_Counts BMI, exposure(Exposure_Days_Md)

est store lin

poisson Total_OSTRC_Alla_Segm_NDL_Counts BMI BMI2,
exposure(Exposure_Days_Md)

est store quad

lrtest lin quad //P=0.0000***

drop BMI2


poisson Total_OSTRC_Alla_Segm_DL_Counts BMI, exposure(Exposure_Days_Md)

est store lin

poisson Total_OSTRC_Alla_Segm_DL_Counts BMI BMI2, exposure(Exposure_Days_Md)

est store quad

lrtest lin quad //P=0.0013

drop BMI2


nbreg Total_OSTRC_Alla_Segm_NDL_Counts c.BMI c.BMI#c.BMI,
exposure(Exposure_Days_Md) vce(cluster Subject) irr

margins, at(BMI=(15(1)35)) atmeans

marginsplot

//Conclusion: BMI is not linear for any leg


//The variable ADF

poisson Total_OSTRC_Alla_Segm_NDL_Counts NDL_ADF,
exposure(Exposure_Days_Md)

predict log_mu, xb

lowess log_mu NDL_ADF, mean //the curve is relatively straight

twoway (scatter log_mu NDL_ADF, msymbol(o))(lowess log_mu NDL_ADF,
bwidth(0.8)),title("lowess b=0.8") // the curve is linear

drop log_mu


poisson Total_OSTRC_Alla_Segm_DL_Counts DL_ADF, exposure(Exposure_Days_Md)

predict log_mu, xb
```

```
lowess log_mu DL_ADF, mean //the curve is relatively straight

twoway (scatter log_mu DL_ADF, msymbol(o))(lowess log_mu DL_ADF,
bwidth(0.8)),title("lowess b=0.8") //the curve is relative ok

drop log_mu


gen NDL_ADF2=NDL_ADF^2

poisson Total_OSTRC_Alla_Segm_NDL_Counts NDL_ADF NDL_ADF2,
exposure(Exposure_Days_Md) irr // n.s

drop NDL_ADF2


gen DL_ADF2=DL_ADF^2

poisson Total_OSTRC_Alla_Segm_DL_Counts DL_ADF DL_ADF2,
exposure(Exposure_Days_Md) irr // p=0.003***

drop DL_ADF2



poisson Total_OSTRC_Alla_Segm_NDL_Counts NDL_ADF,
exposure(Exposure_Days_Md)

est store lin

poisson Total_OSTRC_Alla_Segm_NDL_Counts NDL_ADF NDL_ADF2,
exposure(Exposure_Days_Md)

est store quad

lrtest lin quad //P=0.25

drop NDL_ADF2


poisson Total_OSTRC_Alla_Segm_DL_Counts DL_ADF, exposure(Exposure_Days_Md)

est store lin

poisson Total_OSTRC_Alla_Segm_DL_Counts DL_ADF DL_ADF2,
exposure(Exposure_Days_Md)

est store quad

lrtest lin quad //P=0.04

drop DL_ADF2


//Conclusion: ADF is not linear for the DL but for the NDL the LR test but the curves are ok.
```

//The variable CLAM (

poisson Total_OSTRC_Alla_Segm_NDL_Counts NDL_Nm3BWm_100, exposure(Exposure_Days_Md)

predict log_mu, xb

lowess log_mu NDL_Nm3BWm_100, mean //the curve is not totally straight but has an overall linear appearance

twoway (scatter log_mu NDL_Nm3BWm_100, msymbol(o))(lowess log_mu NDL_Nm3BWm_100, bwidth(0.8)),title("lowess b=0.8") //The curve is relatively linear

drop log_mu

poisson Total_OSTRC_Alla_Segm_DL_Counts DL_Nm3BWm_100, exposure(Exposure_Days_Md)

predict log_mu, xb

lowess log_mu DL_Nm3BWm_100, mean //the curve is not totally straight but has an overall linear appearance

twoway (scatter log_mu DL_Nm3BWm_100, msymbol(o))(lowess log_mu DL_Nm3BWm_100, bwidth(0.8)),title("lowess b=0.8") //The curve is relatively linear

drop log_mu

gen NDL_Nm3BWm_100_2=NDL_Nm3BWm_100^2

poisson Total_OSTRC_Alla_Segm_NDL_Counts NDL_Nm3BWm_100 NDL_Nm3BWm_100_2, exposure(Exposure_Days_Md) irr // n.s

drop NDL_Nm3BWm_100_2

gen DL_Nm3BWm_100_2=DL_Nm3BWm_100^2

poisson Total_OSTRC_Alla_Segm_DL_Counts DL_Nm3BWm_100 DL_Nm3BWm_100_2, exposure(Exposure_Days_Md) irr // n.s

drop DL_Nm3BWm_100_2

poisson Total_OSTRC_Alla_Segm_NDL_Counts NDL_Nm3BWm_100, exposure(Exposure_Days_Md)

est store lin

poisson Total_OSTRC_Alla_Segm_NDL_Counts NDL_Nm3BWm_100 NDL_Nm3BWm_100_2, exposure(Exposure_Days_Md)

est store quad

```
lrtest lin quad //P=0.21

drop NDL_Nm3BWm_100_2


poisson Total_OSTRC_Alla_Segm_DL_Counts DL_Nm3BWm_100,
exposure(Exposure_Days_Md)

est store lin

poisson Total_OSTRC_Alla_Segm_DL_Counts DL_Nm3BWm_100 DL_Nm3BWm_100_2,
exposure(Exposure_Days_Md)

est store quad

lrtest lin quad //P=0.11

drop DL_Nm3BWm_100_2


//Conclusion: CLAM is linear with the outcome variable




//The variable AFAQ

poisson Total_OSTRC_Alla_Segm_NDL_Counts AFAQ, exposure(Exposure_Days_Md)

predict log_mu, xb

lowess log_mu AFAQ, mean //the curve is relatively straight

twoway (scatter log_mu AFAQ, msymbol(o))(lowess log_mu AFAQ,
bwidth(0.8)),title("lowess b=0.8") //Looks relatively linear

drop log_mu


poisson Total_OSTRC_Alla_Segm_DL_Counts AFAQ, exposure(Exposure_Days_Md)

predict log_mu, xb

lowess log_mu AFAQ, mean //the curve is relatively straight

twoway (scatter log_mu AFAQ, msymbol(o))(lowess log_mu AFAQ,
bwidth(0.8)),title("lowess b=0.8") //Looks relatively linear

drop log_mu


gen AFAQ2=AFAQ^2

poisson Total_OSTRC_Alla_Segm_NDL_Counts AFAQ AFAQ2,
exposure(Exposure_Days_Md) irr // p=0.03***
```

drop AFAQ2

gen AFAQ2=AFAQ^2

poisson Total_OSTRC_Alla_Segm_DL_Counts AFAQ AFAQ2, exposure(Exposure_Days_Md) irr // n.s

drop AFAQ2

poisson Total_OSTRC_Alla_Segm_NDL_Counts AFAQ, exposure(Exposure_Days_Md)

est store lin

poisson Total_OSTRC_Alla_Segm_NDL_Counts AFAQ AFAQ2, exposure(Exposure_Days_Md)

est store quad

lrtest lin quad //P=0.04***

drop AFAQ2

poisson Total_OSTRC_Alla_Segm_DL_Counts AFAQ, exposure(Exposure_Days_Md)

est store lin

poisson Total_OSTRC_Alla_Segm_DL_Counts AFAQ AFAQ2, exposure(Exposure_Days_Md)

est store quad

lrtest lin quad //P=0.60

drop AFAQ2

//Conclusion: For the LR test the DL is n.s but the NDL are. The curves look good for both legs

//The variable PSS14

poisson Total_OSTRC_Alla_Segm_NDL_Counts PSS14, exposure(Exposure_Days_Md)

predict log_mu, xb

lowess log_mu PSS14, mean //the curve is relatively straight

twoway (scatter log_mu PSS14, msymbol(o))(lowess log_mu PSS14, bwidth(0.8)),title("lowess b=0.8") //looks relatively ok

drop log_mu

```
poisson Total_OSTRC_Alla_Segm_DL_Counts PSS14, exposure(Exposure_Days_Md)

predict log_mu, xb

lowess log_mu PSS14, mean //the curve is relatively straight

twoway (scatter log_mu PSS14, msymbol(o))(lowess log_mu PSS14,
bwidth(0.8)),title("lowess b=0.8") //looks relatively ok

drop log_mu


gen PSS142=PSS14^2

poisson Total_OSTRC_Alla_Segm_NDL_Counts PSS14 PSS142,
exposure(Exposure_Days_Md) irr // n.s

drop PSS142


gen PSS142=PSS14^2

poisson Total_OSTRC_Alla_Segm_DL_Counts PSS14 PSS142,
exposure(Exposure_Days_Md) irr // p=0.005

drop PSS142



poisson Total_OSTRC_Alla_Segm_NDL_Counts PSS14, exposure(Exposure_Days_Md)

est store lin

poisson Total_OSTRC_Alla_Segm_NDL_Counts PSS14 PSS142,
exposure(Exposure_Days_Md)

est store quad

lrtest lin quad //P=0.06

drop PSS142


poisson Total_OSTRC_Alla_Segm_DL_Counts PSS14, exposure(Exposure_Days_Md)

est store lin

poisson Total_OSTRC_Alla_Segm_DL_Counts PSS14 PSS142,
exposure(Exposure_Days_Md)

est store quad

lrtest lin quad //P=0.003****

drop PSS142
```

//Conclusion: PSS 14 is n.s for the NDL but significant for the DL. The curves look ok

//The variable GAD-7

poisson Total_OSTRC_Alla_Segm_NDL_Counts GAD7, exposure(Exposure_Days_Md) //

predict log_mu, xb

lowess log_mu GAD7, mean // the curve is relatively straight

twoway (scatter log_mu GAD7, msymbol(o))(lowess log_mu GAD7, bwidth(0.8)),title("lowess b=0.8") //looks ok but have a small nonlinearity with small values

drop log_mu

poisson Total_OSTRC_Alla_Segm_DL_Counts GAD7, exposure(Exposure_Days_Md)

predict log_mu, xb

lowess log_mu GAD7, mean //the curve is relatively straight

twoway (scatter log_mu GAD7, msymbol(o))(lowess log_mu GAD7, bwidth(0.8)),title("lowess b=0.8") //looks ok but have a small nonlinearity with small values

drop log_mu

gen GAD72=GAD7^2

poisson Total_OSTRC_Alla_Segm_NDL_Counts GAD7 GAD72, exposure(Exposure_Days_Md) irr // p=0.02

drop GAD72

gen GAD72=GAD7^2

poisson Total_OSTRC_Alla_Segm_DL_Counts GAD7 GAD72, exposure(Exposure_Days_Md) irr // p=0.003

drop GAD72

poisson Total_OSTRC_Alla_Segm_NDL_Counts GAD7, exposure(Exposure_Days_Md)

est store lin

poisson Total_OSTRC_Alla_Segm_NDL_Counts GAD7 GAD72, exposure(Exposure_Days_Md)

est store quad

lrtest lin quad //P=0.02****

```
drop GAD72

poisson Total_OSTRC_Alla_Segm_DL_Counts GAD7, exposure(Exposure_Days_Md)
est store lin
poisson Total_OSTRC_Alla_Segm_DL_Counts GAD7 GAD72,
exposure(Exposure_Days_Md)
est store quad
lrtest lin quad //P=0.002****
drop GAD72
//Conclusion: GAD is not linear


//The variable PSQI
poisson Total_OSTRC_Alla_Segm_NDL_Counts PSQI, exposure(Exposure_Days_Md) //
predict log_mu, xb
lowess log_mu PSQI, mean // the curve is relatively straight
twoway (scatter log_mu PSQI, msymbol(o))(lowess log_mu PSQI, bwidth(0.8)),title("lowess
b=0.8") //does not look linear
drop log_mu

poisson Total_OSTRC_Alla_Segm_DL_Counts PSQI, exposure(Exposure_Days_Md)
predict log_mu, xb
lowess log_mu PSQI, mean //the curve is relatively straight
twoway (scatter log_mu PSQI, msymbol(o))(lowess log_mu PSQI, bwidth(0.8)),title("lowess
b=0.8") //does not look linear
drop log_mu

gen PSQI2=PSQI^2
poisson Total_OSTRC_Alla_Segm_NDL_Counts PSQI PSQI2,
exposure(Exposure_Days_Md) irr // p=0.001
drop PSQI2

gen PSQI2=PSQI^2
```

poisson Total_OSTRC_Alla_Segm_DL_Counts PSQI PSQI2, exposure(Exposure_Days_Md) irr // p=0.00

drop PSQI2

poisson Total_OSTRC_Alla_Segm_NDL_Counts PSQI, exposure(Exposure_Days_Md)

est store lin

poisson Total_OSTRC_Alla_Segm_NDL_Counts PSQI PSQI2, exposure(Exposure_Days_Md)

est store quad

lrtest lin quad //P=0.001****

drop PSQI2

poisson Total_OSTRC_Alla_Segm_DL_Counts PSQI, exposure(Exposure_Days_Md)

est store lin

poisson Total_OSTRC_Alla_Segm_DL_Counts PSQI PSQI2, exposure(Exposure_Days_Md)

est store quad

lrtest lin quad //P=0.001****

drop PSQI2

//Conclusion: PSQI is not linear

//OVERALL CONCLUSION FOR THE EVALUATION OF THE LINEAR RELATIONSHIP WITH THE OUTCOME VARIABLE ON THE LOG SCALE:

//Conclusion: Age is not linear for the NDL but for the DL on the LR test but the curves look bad (Age will be categorized)

//Conclusion: Weight is not linear for any leg

//Conclusion: Length is not linear for the NDL but for the DL

//Conclusion: BMI is not linear for any leg BMI will be categorized or used with a quadratic term)

//Conclusion: ADF is not linear for the DL but for the NDL regarding the LR test but the curves are ok. (The variable could be used as it is)

//Conclusion: CLAM is linear with the outcome variable

//Conclusion: AFAQ is for the DL n.s but for the NDL significant. The curves look good for both legs (The variable could be used as it is)

//Conclusion: PSS 14 is n.s for the NDL but significant for the DL. The curves look ok (The variable could be used as it is)

//Conclusion: GAD is not linear (The variable will be categorized)

//Conclusion: PSQI is not linear (The variable will be categorized)

*STEP 4. CHECKING FOR COLLINEARITY

//To evaluate the values:

//VIF > 10 = a strong collinearity

//VIF > 5 = might be worrying, especially if there is a lot of high values

//Tolerance (= 1/VIF), low values (< 0.1) might be a red flag.

//A. starting with an overall check of the continuous and categorical variables.

pwcorr Age Weightkg Lengthm BMI NDL_Nm3BWm_100 NDL_ADF AFAQ PSS14,sig

pwcorr Age Weightkg Lengthm BMI DL_Nm3BWm_100 DL_ADF AFAQ PSS14,sig

pwcorr Age Weightkg BMI NDL_Nm3BWm_100 NDL_ADF AFAQ PSS14,sig //BMI and weight 0.75

pwcorr Age Weightkg BMI DL_Nm3BWm_100 DL_ADF AFAQ PSS14,sig

pwcorr Age Lengthm BMI NDL_Nm3BWm_100 NDL_ADF AFAQ PSS14,sig

pwcorr Age Lengthm BMI DL_Nm3BWm_100 DL_ADF AFAQ PSS14,sig

pwcorr Age BMI NDL_Nm3BWm_100 NDL_ADF AFAQ PSS14,sig

pwcorr Age BMI DL_Nm3BWm_100 DL_ADF AFAQ PSS14,sig //as I know length and weight has a correlation to BMI those are eliminated.

//Length by itself is not correlated to BMI so it could be left in the model for now. All correlation coefficients are <0.8.

tab div NDL_SLSfailpass, chi2 //p=0.06

tab div DL_SLSfailpass, chi2 //p=0.26

tab div PSQI_cat2, chi2 //p=0.26

tab div GAD7_cat2, chi2 //p=0.20

tab NDL_SLSfailpass PSQI_cat2, chi2 //p=0.20

tab DL_SLSfailpass PSQI_cat2, chi2 //p=0.38

tab NDL_SLSfailpass GAD7_cat2, chi2 //p=0.98

tab DL_SLSfailpass GAD7_cat2, chi2 //p=0.15

tab PSQI_cat2 GAD7_cat2, chi2 //p=0.000

//chi2 is not a correlation measure but says something about the association between two variables. PSQI and GAD has a strong

//association and should be kept in mind for the estat vif test.

//B. A more specific test for collinearity.

regress Total_OSTRC_Alla_Segm_NDL_Counts div Age Weightkg Lengthm BMI NDL_SLSfailpass NDL_ADF NDL_Nm3BWm_100 AFAQ PSS14 PSQI_cat2 GAD7_cat2

estat vif

regress Total_OSTRC_Alla_Segm_NDL_Counts div Age Lengthm BMI NDL_SLSfailpass NDL_ADF NDL_Nm3BWm_100 AFAQ PSS14 PSQI_cat2 GAD7_cat2

estat vif //no problem with length and BMI together

regress Total_OSTRC_Alla_Segm_NDL_Counts div Age Weightkg BMI NDL_SLSfailpass NDL_ADF NDL_Nm3BWm_100 AFAQ PSS14 PSQI_cat2 GAD7_cat2

estat vif //No problem with weight and BMI together

regress Total_OSTRC_Alla_Segm_NDL_Counts div Age BMI NDL_SLSfailpass NDL_ADF NDL_Nm3BWm_100 AFAQ PSS14 PSQI_cat2 GAD7_cat2

estat vif

regress Total_OSTRC_Alla_Segm_NDL_Counts div Age BMI NDL_SLSfailpass NDL_ADF NDL_Nm3BWm_100 AFAQ PSS14 PSQI GAD7 //GAD and PSQI as continuous variables

estat vif

//High VIF values over 100 when weight, length and BMI are included in the same calculation.

//If BMI and length is combined the VIF is 1.0 and if weight and BMI are combined VIF is 2.5.

//Conclusion: BMI can in a full model be combined with either weight or length but not for all three together

regress Total_OSTRC_Alla_Segm_DL_Counts div Age Weightkg Lengthm BMI DL_SLSfailpass DL_ADF DL_Nm3BWm_100 AFAQ PSS14 PSQI_cat2 GAD7_cat2

estat vif

regress Total_OSTRC_Alla_Segm_DL_Counts div Age Lengthm BMI DL_SLSfailpass DL_ADF DL_Nm3BWm_100 AFAQ PSS14 PSQI_cat2 GAD7_cat2

estat vif

regress Total_OSTRC_Alla_Segm_DL_Counts div Age Weightkg BMI DL_SLSfailpass DL_ADF DL_Nm3BWm_100 AFAQ PSS14 PSQI_cat2 GAD7_cat2

estat vif

regress Total_OSTRC_Alla_Segm_DL_Counts div Age BMI DL_SLSfailpass DL_ADF DL_Nm3BWm_100 AFAQ PSS14 PSQI_cat2 GAD7_cat2

estat vif

regress Total_OSTRC_Alla_Segm_DL_Counts div Age BMI DL_SLSfailpass DL_ADF DL_Nm3BWm_100 AFAQ PSS14 PSQI GAD7 //GAD and PSQI as continuous variables

estat vif

//High VIF values over 100 when weight, length and BMI are included in the same calculation.

//If BMI and length is combined the VIF is 1.0 and if weight and BMI are combined VIF is 2.5. //Conclusion: BMI can in a full model be combined with either weight or length but not for all three together

*STEP 5. OVERDISPERSION

//If the variance is not equal to the mean you have an overdispersion, Var(Y)>E(Y). If so, there is a risk to underestimate the SE and to receive small p-values.

//if so, the solution is to change to a nbreg or to correct the SE with vce(robust)

//A. Looking at the crude values for the outcome variable

summarize Total_OSTRC_Alla_Segm_NDL_Counts, detail

summarize Total_OSTRC_Alla_Segm_DL_Counts, detail //Conclusion: The mean is not equal to the variance, this could be a sign of overdispersion

//B. Comparing the Likelihood ratio-test (Poisson) with the negative binominal regression (nbreg), this should be tested with a "reasonable" and "realistic" presumptive Model.

//To evaluate the output:

//Is alpha = 0 ??. If the hypothesis is rejected (p=<0.05) there is overdispersion and I should use a nbreg, if the hypothesis cannot be rejected (p>0.05)

//there is no overdispersion and a poisson regression is enough.

//α ≈ 0 → almost no overdispersion. α ≈ 0.1–0.5 → weak overdispersion, Poisson could be ok sometimes. α > 1 → obvious overdispersion nbreg much better.

//α several times larger than 1 → extreme overdispersion.

nbreg Total_OSTRC_Alla_Segm_NDL_Counts div Age Lengthm BMI NDL_SLSfailpass NDL_ADF NDL_Nm3BWm_100 AFAQ PSS14 PSQI_cat2 GAD7_cat2, exposure(Exposure_Days_Md) irr //alpha=0.93 , p=0.000

nbreg Total_OSTRC_Alla_Segm_NDL_Counts div Age Lengthm BMI NDL_SLSfailpass NDL_ADF NDL_Nm3BWm_100 AFAQ PSS14 PSQI GAD7, exposure(Exposure_Days_Md) irr //alpha=0.93 , p=0.000

nbreg Total_OSTRC_Alla_Segm_NDL_Counts div Age Weightkg BMI NDL_SLSfailpass NDL_ADF NDL_Nm3BWm_100 AFAQ PSS14 PSQI_cat2 GAD7_cat2, exposure(Exposure_Days_Md) irr //alpha=0.93 , p=0.000

nbreg Total_OSTRC_Alla_Segm_NDL_Counts div Age Weightkg BMI NDL_SLSfailpass NDL_ADF NDL_Nm3BWm_100 AFAQ PSS14 PSQI GAD7, exposure (Exposure_Days_Md) irr //alpha=0.93 , p=0.000

nbreg Total_OSTRC_Alla_Segm_NDL_Counts div Age BMI NDL_SLSfailpass NDL_ADF NDL_Nm3BWm_100 AFAQ PSS14 PSQI_cat2 GAD7_cat2, exposure(Exposure_Days_Md) irr //alpha=0.93 , p=0.000

nbreg Total_OSTRC_Alla_Segm_NDL_Counts div Age BMI NDL_SLSfailpass NDL_ADF NDL_Nm3BWm_100 AFAQ PSS14 PSQI GAD7, exposure(Exposure_Days_Md) irr //alpha=0.93 , p=0.000

nbreg Total_OSTRC_Alla_Segm_DL_Counts div Age Lengthm BMI NDL_SLSfailpass DL_ADF DL_Nm3BWm_100 AFAQ PSS14 PSQI_cat2 GAD7_cat2, exposure(Exposure_Days_Md) irr //alpha=0.89 , p=0.00

nbreg Total_OSTRC_Alla_Segm_DL_Counts div Age Lengthm BMI NDL_SLSfailpass DL_ADF DL_Nm3BWm_100 AFAQ PSS14 PSQI GAD7, exposure(Exposure_Days_Md) irr //alpha=0.89 , p=0.00

nbreg Total_OSTRC_Alla_Segm_DL_Counts div Age Weightkg BMI DL_SLSfailpass DL_ADF DL_Nm3BWm_100 AFAQ PSS14 PSQI_cat2 GAD7_cat2, exposure(Exposure_Days_Md) irr //alpha=0.88, p=0.00

nbreg Total_OSTRC_Alla_Segm_DL_Counts div Age Weightkg BMI DL_SLSfailpass DL_ADF DL_Nm3BWm_100 AFAQ PSS14 PSQI GAD7, exposure(Exposure_Days_Md) irr //alpha=0.88, p=0.00

nbreg Total_OSTRC_Alla_Segm_DL_Counts div Age BMI DL_SLSfailpass DL_ADF DL_Nm3BWm_100 AFAQ PSS14 PSQI_cat2 GAD7_cat2, exposure(Exposure_Days_Md) irr //alpha=0.88, p=0.00

nbreg Total_OSTRC_Alla_Segm_DL_Counts div Age BMI DL_SLSfailpass DL_ADF DL_Nm3BWm_100 AFAQ PSS14 PSQI GAD7, exposure(Exposure_Days_Md) irr //alpha=0.89, p=0.00

//Conclusion: There is obvious overdispersion

//C. Comparing the deviance/ pearson chi2 with df

//if the p values are >0.05 there is no overdispersion.

//You can also look at the quota Goodness of fit/df. If =0 to 1 no overdispersion, if =1.5 obvious overdispersion, if <1 under dispersion

poisson Total_OSTRC_Alla_Segm_NDL_Counts div Age Lengthm BMI NDL_SLSfailpass NDL_ADF NDL_Nm3BWm_100 AFAQ PSS14 PSQI_cat2 GAD7_cat2, exposure(Exposure_Days_Md) irr

estat gof, pearson // Goodness of fit=919, Df=230,p=0.0000. 919/230=3.99-->overdispersion

poisson Total_OSTRC_Alla_Segm_NDL_Counts div Age Lengthm BMI NDL_SLSfailpass NDL_ADF NDL_Nm3BWm_100 AFAQ PSS14 PSQI GAD7, exposure(Exposure_Days_Md) irr

estat gof, pearson

poisson Total_OSTRC_Alla_Segm_NDL_Counts div Age Weightkg BMI NDL_SLSfailpass NDL_ADF NDL_Nm3BWm_100 AFAQ PSS14 PSQI_cat2 GAD7_cat2, exposure(Exposure_Days_Md) irr

estat gof, pearson

poisson Total_OSTRC_Alla_Segm_NDL_Counts div Age Weightkg BMI NDL_SLSfailpass NDL_ADF NDL_Nm3BWm_100 AFAQ PSS14 PSQI GAD7, exposure(Exposure_Days_Md) irr

estat gof, pearson

poisson Total_OSTRC_Alla_Segm_NDL_Counts div Age BMI NDL_SLSfailpass NDL_ADF NDL_Nm3BWm_100 AFAQ PSS14 PSQI_cat2 GAD7_cat2, exposure(Exposure_Days_Md) irr

estat gof, pearson

poisson Total_OSTRC_Alla_Segm_NDL_Counts div Age BMI NDL_SLSfailpass NDL_ADF NDL_Nm3BWm_100 AFAQ PSS14 PSQI GAD7, exposure(Exposure_Days_Md) irr

estat gof, pearson //Obvious overdispersion for all models

poisson Total_OSTRC_Alla_Segm_DL_Counts div Age Lengthm BMI NDL_SLSfailpass DL_ADF DL_Nm3BWm_100 AFAQ PSS14 PSQI_cat2 GAD7_cat2, exposure(Exposure_Days_Md) irr

estat gof, pearson //Goodness of fit=852, Df=229,p=0.0000, 852/229-->overdispersion

poisson Total_OSTRC_Alla_Segm_DL_Counts div Age Lengthm BMI NDL_SLSfailpass DL_ADF DL_Nm3BWm_100 AFAQ PSS14 PSQI GAD7, exposure(Exposure_Days_Md) irr

estat gof, pearson

poisson Total_OSTRC_Alla_Segm_DL_Counts div Age Weightkg BMI DL_SLSfailpass DL_ADF DL_Nm3BWm_100 AFAQ PSS14 PSQI_cat2 GAD7_cat2, exposure(Exposure_Days_Md) irr

estat gof, pearson

poisson Total_OSTRC_Alla_Segm_DL_Counts div Age Weightkg BMI DL_SLSfailpass DL_ADF DL_Nm3BWm_100 AFAQ PSS14 PSQI GAD7, exposure(Exposure_Days_Md) irr

estat gof, pearson

poisson Total_OSTRC_Alla_Segm_DL_Counts div Age BMI DL_SLSfailpass DL_ADF DL_Nm3BWm_100 AFAQ PSS14 PSQI_cat2 GAD7_cat2, exposure(Exposure_Days_Md) irr

estat gof, pearson

poisson Total_OSTRC_Alla_Segm_DL_Counts div Age BMI DL_SLSfailpass DL_ADF DL_Nm3BWm_100 AFAQ PSS14 PSQI GAD7, exposure(Exposure_Days_Md) irr

estat gof, pearson //Obvious overdispersion for all models

//OVERALL CONCLUSION: There is an obvious overdispersion for all Models.

*STEP 5. UNIVARIATE MODELS

//Given that the data contained clustered observations due to repeated measurements per individual, all analyses employed

//robust standard errors with clustering at the individual level (vce(cluster Subject)). nbreg is also used due to overdispersion

// a. Division

nbreg Total_OSTRC_Alla_Segm_NDL_Counts div, exposure(Exposure_Days_Md) vce(cluster Subject) irr

nbreg Total_OSTRC_Alla_Segm_NDL_Counts i.div, exposure(Exposure_Days_Md) vce(cluster Subject) irr

nbreg Total_OSTRC_Alla_Segm_DL_Counts div, exposure(Exposure_Days_Md) vce(cluster Subject) irr

nbreg Total_OSTRC_Alla_Segm_DL_Counts i.div, exposure(Exposure_Days_Md) vce(cluster Subject) irr

//b. Age

nbreg Total_OSTRC_Alla_Segm_NDL_Counts Age_cat, exposure(Exposure_Days_Md) vce(cluster Subject) irr

nbreg Total_OSTRC_Alla_Segm_NDL_Counts i.Age_cat, exposure(Exposure_Days_Md) vce(cluster Subject) irr

nbreg Total_OSTRC_Alla_Segm_DL_Counts Age_cat, exposure(Exposure_Days_Md) vce(cluster Subject) irr

nbreg Total_OSTRC_Alla_Segm_DL_Counts i.Age_cat, exposure(Exposure_Days_Md) vce(cluster Subject) irr

//c. Length

nbreg Total_OSTRC_Alla_Segm_NDL_Counts Length100, exposure(Exposure_Days_Md) vce(cluster Subject) irr

nbreg Total_OSTRC_Alla_Segm_DL_Counts Length100, exposure(Exposure_Days_Md) vce(cluster Subject) irr

//d. BMI_cat

nbreg Total_OSTRC_Alla_Segm_NDL_Counts BMI_cat, exposure(Exposure_Days_Md) vce(cluster Subject) irr

nbreg Total_OSTRC_Alla_Segm_NDL_Counts i.BMI_cat, exposure(Exposure_Days_Md) vce(cluster Subject) irr

nbreg Total_OSTRC_Alla_Segm_DL_Counts BMI_cat, exposure(Exposure_Days_Md) vce(cluster Subject) irr

nbreg Total_OSTRC_Alla_Segm_DL_Counts i.BMI_cat, exposure(Exposure_Days_Md) vce(cluster Subject) irr

//e. SLS fail/pass

nbreg Total_OSTRC_Alla_Segm_NDL_Counts NDL_SLSfailpass, exposure(Exposure_Days_Md) vce(cluster Subject) irr

nbreg TLI_Alla_Segment_DL DL_SLSfailpass, exposure(Exposure_Days_Md) vce(cluster Subject) irr

//f. ADF

nbreg Total_OSTRC_Alla_Segm_NDL_Counts NDL_ADF , exposure(Exposure_Days_Md) vce(cluster Subject) irr

nbreg Total_OSTRC_Alla_Segm_DL_Counts DL_ADF , exposure(Exposure_Days_Md) vce(cluster Subject) irr

//g. CLAM

nbreg Total_OSTRC_Alla_Segm_NDL_Counts NDL_Nm3BWm_100, exposure(Exposure_Days_Md) vce(cluster Subject) irr

nbreg Total_OSTRC_Alla_Segm_DL_Counts DL_Nm3BWm_100, exposure(Exposure_Days_Md) vce(cluster Subject) irr

//h. AFAQ

nbreg Total_OSTRC_Alla_Segm_NDL_Counts AFAQ , exposure(Exposure_Days_Md) vce(cluster Subject) irr

nbreg Total_OSTRC_Alla_Segm_DL_Counts AFAQ , exposure(Exposure_Days_Md) vce(cluster Subject) irr

//i. PSS14

nbreg Total_OSTRC_Alla_Segm_NDL_Counts PSS14 , exposure(Exposure_Days_Md) vce(cluster Subject) irr

nbreg Total_OSTRC_Alla_Segm_DL_Counts PSS14 , exposure(Exposure_Days_Md) vce(cluster Subject) irr

//j. PSQI

nbreg Total_OSTRC_Alla_Segm_NDL_Counts PSQI_cat2 , exposure(Exposure_Days_Md) vce(cluster Subject) irr

nbreg Total_OSTRC_Alla_Segm_DL_Counts PSQI_cat2 , exposure(Exposure_Days_Md) vce(cluster Subject) irr

//k. GAD7

nbreg Total_OSTRC_Alla_Segm_NDL_Counts GAD7_cat2 , exposure(Exposure_Days_Md) vce(cluster Subject) irr

nbreg Total_OSTRC_Alla_Segm_NDL_Counts i.GAD7_cat2 , exposure(Exposure_Days_Md) vce(cluster Subject) irr

nbreg Total_OSTRC_Alla_Segm_DL_Counts GAD7_cat2 , exposure(Exposure_Days_Md) vce(cluster Subject) irr

nbreg Total_OSTRC_Alla_Segm_DL_Counts i.GAD7_cat2 , exposure(Exposure_Days_Md) vce(cluster Subject) irr

//l. Weight

nbreg Total_OSTRC_Alla_Segm_NDL_Counts Lengthm, exposure(Exposure_Days_Md) vce(cluster Subject) irr //p=0.433

nbreg Total_OSTRC_Alla_Segm_DL_Counts Lengthm, exposure(Exposure_Days_Md) vce(cluster Subject) irr //p=0.29

*MODEL BUILDING WITH PURPOSEFUL SELECTION FOR OVERUSE INJURY: NON-DOMINANT LEG

*STEP 0. Candidate list

//div: n.s p=0.70. this variable is eliminated due to p values

//Age: An apriori variable selected from the literature

//weight: p=0.43, is not included due to p value but also due to collinearity if weight, Length and BMI are included in the same model. This variable also has issues with non-linearity on the log scale

//Length: n.s 0.43. this variable is eliminated due to p value; it also has issues for the NDL regarding the linearity on the log scale

//BMI: p=0.00 for some categories, is not an apriori variable for overuse injuries ***See discussion below regarding BMI

//The SLS: n.s p=0.11. Stays in the Model

//ADF: n.s p=0.92 this variable is eliminated due to p values

//CLAM: n.s p=0.70 this variable is eliminated due to p values

//AFAQ: P=0.00. The variable stays

//PSS-14:n.s p=0.57 this variable is eliminated due to p values

//PSQI: n.s p=0.85 this variable is eliminated due to p values

//GAD-7: n.s p=0.10 Stays in the Model

*STEP 1. Full base Model

//Starting with a full Model and saves AIC and BIC. AIC/BIC is a reference point. If a reduced model has equal or lower AIC/BIC it is a better balance between

//fit and simplicity. If AIC/BIC increases when a variable is eliminated it probably contributed to the Model even though the p-value was low.

nbreg Total_OSTRC_Alla_Segm_NDL_Counts i.Age_cat NDL_SLSfailpass AFAQ i.GAD7_cat2 ,exposure(Exposure_Days_Md) vce(cluster Subject) irr //without BMI

est store full_1_OSTRC_NDL

estat ic //AIC=1055   BIC=1083

nbreg Total_OSTRC_Alla_Segm_NDL_Counts i.Age_cat i.BMI_cat NDL_SLSfailpass AFAQ i.GAD7_cat2 ,exposure(Exposure_Days_Md) vce(cluster Subject) irr //BMI categorized

est store full_1_OSTRC_NDL

estat ic //AIC=1043   BIC=1077

gen BMI2 = BMI^2

nbreg Total_OSTRC_Alla_Segm_NDL_Counts i.Age_cat BMI BMI2 NDL_SLSfailpass AFAQ i.GAD7_cat2 ,exposure(Exposure_Days_Md) vce(cluster Subject) irr //BMI squared

est store full_1_OSTRC_NDL

estat ic //AIC=1051   BIC=1086

drop BMI2

tab BMI_cat

summarize BMI, detail

histogram BMI, width(1) normal

margins, at(BMI=(18(1)28))

marginsplot, ciopts(lcolor(gs12)) title("Predicerad incidens över BMI") xtitle("BMI") ytitle("Predicerat antal händelser") recast(line)

***Discussion about BMI:

//BMI has very few observations in cat 1, 3 and 4. This means that the extrapolation of a squared term will be meaningless. //Categorization of the variable will make the model more stable, more meaningful and easier to interpret but //the great CI shows that the interpretation must be done with caution. As BMI is not an a priori variable for //overuse injury it could also be excluded with the arguments above.

//CONLUSION: BMI is taken away from the model after sensitivity testing showing CI for BMI ranging from 1.88 to 28

nbreg Total_OSTRC_Alla_Segm_NDL_Counts i.Age_cat NDL_SLSfailpass AFAQ i.GAD7_cat2 ,exposure(Exposure_Days_Md) vce(cluster Subject) irr //this is the full model to start with

est store full_1_OSTRC_NDL

estat ic //AIC=1055 BIC=1083

*STEP 2. Wald test and confounding check for each variable

*The SLS

//a. Wald test:

//Eliminating and testing one variable at a time with the wald test (LR test is invalid for models with robust vce).

//If p<0.05 the variable contributes significantly and it should stay in the model.

//If p>0.05 the variable does not contribute to the model and could be eliminated.

nbreg Total_OSTRC_Alla_Segm_NDL_Counts i.Age_cat NDL_SLSfailpass AFAQ i.GAD7_cat2 ,exposure(Exposure_Days_Md) vce(cluster Subject) irr

est store full_1_OSTRC_NDL

testparm NDL_SLSfailpass //p=0.43

//b. Confounding-check

//Even though a variable is non-significant, it could be a confounder and should therefore stay in the Model

//The rule of thumb is that a change of 10% or more of the IRR is a confounder (comparing the full Model with the reduces Model)

nbreg Total_OSTRC_Alla_Segm_NDL_Counts i.Age_cat AFAQ i.GAD7_cat2 ,exposure(Exposure_Days_Md) vce(cluster Subject) irr //full Model without SLS

est store reduced_1_OSTRC_NDL

est table full_1_OSTRC_NDL reduced_1_OSTRC_NDL, eform b(%9.3f) se(%9.3f) stats(N ll) //no changes >10% is noticed between full_1 and reduced Model. The variable SLS is eliminated

//c. new model without SLS

nbreg Total_OSTRC_Alla_Segm_NDL_Counts i.Age_cat AFAQ i.GAD7_cat2 ,exposure(Exposure_Days_Md) vce(cluster Subject) irr //The new full model

est store full_2_OSTRC_NDL

estat ic //AIC=1054 BIC=1078 If AIC and BIC are the same or lower it is a better balance between fit and simplicity which is good. If AIC and BIC rise

//significantly the variable taken away probably contributed to the model even though the p value was poor.

//CONCLUSION: SLS is taken away

*GAD-7

//a. Wald test

est store full_2_OSTRC_NDL

testparm i.GAD7_cat2 //p=0.59

//b. Confounding check

nbreg Total_OSTRC_Alla_Segm_NDL_Counts i.Age_cat AFAQ, exposure(Exposure_Days_Md) vce(cluster Subject) irr //full model without GAD

est store reduced_2_OSTRC_NDL

est table full_2_OSTRC_NDL reduced_2_OSTRC_NDL, eform b(%9.3f) se(%9.3f) stats(N ll) //no changes >10% is noticed between full_1 and reduced Model. The variable GAD is eliminated

//c. new model without GAD-7

nbreg Total_OSTRC_Alla_Segm_NDL_Counts i.Age_cat AFAQ, exposure(Exposure_Days_Md) vce(cluster Subject) irr //The new full model

est store full_3_OSTRC_NDL

estat ic //AIC=1051, BIC=1068

//CONCLUSION: GAD-7 is taken away

//CONCLUSION: This is the new full Model:

nbreg Total_OSTRC_Alla_Segm_NDL_Counts i.Age_cat AFAQ, exposure(Exposure_Days_Md) vce(cluster Subject) irr //The new full model

*STEP 3. Interaction terms

//If the interaction term is significant and/or gives a theoretical reasonable pattern the interaction terms stay in the Model.

//AIC and BIC in the model with interaction terms is compared to the model without interactions, if AIC decreases a lot and the interaction term is theoretical

//reasonable, the terms stay in the Model.

//The new full model is frugal and does not need to be tested for interaction. On the other hand, for TLI we saved BMI as an a priori variable but not for OSTRC.

//BMI and div had an interaction for TLI. This has also been tested for OSTRC as seen below:

nbreg Total_OSTRC_Alla_Segm_NDL_Counts i.Age_cat i.div##c.BMI i.div##c.BMI2 AFAQ ,exposure(Exposure_Days_Md) vce(cluster Subject) irr //both BMI and BMI2 are significant

testparm i.div#c.BMI i.div#c.BMI2 //n.s p=0.15

margins div, at(BMI=(15(1)35))

marginsplot

//CONCLUSION: No need to test interactions for this Model and the test for interaction

//between div and BMI was non-significant.

*STEP 4. Model fit and diagnostic

//Alt 2: A more complete diagnostic where glm with the nbinom-family is used to receive Pearson residuals/deviance and influential statistics

//OBS! this is just for diagnostics and should not replace nbreg Modelling

glm Total_OSTRC_Alla_Segm_NDL_Counts i.Age_cat AFAQ, family(nbinomial `a') link(log) exposure(Exposure_Days_Md) nolog

predict muhat_glm_OSTRC_NDL, mu

predict pearson_glm_OSTRC_NDL, pearson

predict devres_glm_OSTRC_NDL, deviance

predict hat_glm_OSTRC_NDL, hat

predict cook_glm_OSTRC_NDL, cooksd

scatter pearson_glm_OSTRC_NDL muhat_glm_OSTRC_NDL, yline(0) title("Pearson residuals (glm)") //Most of the observations are localized around -1 to 2. But there are some outliers with residuals between 3 to 4

list Subject muhat_glm_OSTRC_NDL pearson_glm_OSTRC_NDL if abs(pearson_glm) > 3 //Subject 20, 81, 165, 262, 172, 216, 225, 168, 180, 208, 55, 206 have residuals >3 and are outliers

list if Subject == 20 | Subject == 81 | Subject == 165 | Subject == 262 | Subject == 172| Subject == 216| Subject == 225| Subject == 168 | Subject == 180| Subject == 208| Subject == 206 | Subject == 55

list Subject Total_OSTRC_Alla_Segm_NDL_Counts Age_cat AFAQ pearson_glm_OSTRC_NDL if Subject == 20 | Subject == 81 | Subject == 165 | Subject == 262 | Subject == 172| Subject == 216| Subject == 225| Subject == 168 | Subject == 180| Subject == 208| Subject == 206 | Subject == 55

//Conclusion:No data has been recorded incorrectly.

nbreg Total_OSTRC_Alla_Segm_NDL_Counts i.Age_cat AFAQ, exposure(Exposure_Days_Md) vce(cluster Subject) irr //Exclusion of outliers should be done on the nbreg model with vce

nbreg Total_OSTRC_Alla_Segm_NDL_Counts i.Age_cat AFAQ if Subject!=20 & Subject!=81 & Subject!=165 & Subject!=262 & Subject!=172 & Subject!=216 & Subject!=225 & Subject!=168 & Subject!=180 & Subject!=208 & Subject!=55 & Subject!=206, exposure(Exposure_Days_Md) vce(cluster Subject) irr

//exclusion of outliers

//Conclusion: When eliminating all outliers at once my general conclusion still holds, no greater change//in direction, p value, pseudo R2, wald

//b. Analysis of deviance residuals

//Deviance residuals: Values greater than 2-3 could indicate deviance.

scatter devres_glm_OSTRC_NDL muhat_glm_OSTRC_NDL, yline(0) title("Deviance residuals (glm)") //Residuals are in reasonable limits (<3), the curves have no systematic patterns,

//no signs of gross misspecification.

//Leverage grafs (hat values): influential points

//How much an observation affects its prediction, 2p/n is a simple cut off. in my case: di (2*5)/243=.04

//That will say: hat-values greater than 0.04 could be values to check.

scatter hat_glm_OSTRC_NDL muhat_glm_OSTRC_NDL, title("Leverage vs. Predicted values") ytitle("Leverage (hat)") xtitle("Predicted counts (muhat)") // Three points are >0.04

list Subject hat_glm_OSTRC_NDL muhat_glm_OSTRC_NDL if hat_glm_OSTRC_NDL > 0.04 //Subject 162 have missing values, 43, 80 and 55 has high leverage

nbreg Total_OSTRC_Alla_Segm_NDL_Counts i.Age_cat AFAQ, exposure(Exposure_Days_Md) vce(cluster Subject) irr //Exclusion of influential points should be done on the nbreg model with vce

nbreg Total_OSTRC_Alla_Segm_NDL_Counts i.Age_cat AFAQ if Subject!=162 & Subject!=43 & Subject!=80 & Subject!=55, exposure(Exposure_Days_Md) vce(cluster Subject) irr //exclusion of influential points

//Conclusion: the model is not affected by exclusion of influential points with high leverage

sort cook_glm_OSTRC_NDL

list Subject Total_OSTRC_Alla_Segm_NDL_Counts i.Age_cat AFAQ Exposure_Days_Md if _n > _N-5

// No incorrect data found. 0.02 is higher than normal but still far under the critical limit 1. Subject 185, 181, 182, 16 has high values and 162 has missing values

nbreg Total_OSTRC_Alla_Segm_NDL_Counts i.Age_cat AFAQ, exposure(Exposure_Days_Md) vce(cluster Subject) irr

nbreg Total_OSTRC_Alla_Segm_NDL_Counts i.Age_cat AFAQ if Subject!=185 & Subject!=181 & Subject!=182 & Subject!=16, exposure(Exposure_Days_Md) vce(cluster Subject) irr

//exclusion of those subjects does not change the Model except for the n.s age cat that goes from 1.02 to 0.97

//Conclusion: The model is not affected by those influential points

//Sort cook's distance (influential points)

sort cook_glm_OSTRC_NDL

list Subject cook_glm_OSTRC_NDL muhat_glm_OSTRC_NDL pearson_glm_OSTRC_NDL devres_glm_OSTRC_NDL hat_glm_OSTRC_NDL in -10/l //

list Subject cook_glm_OSTRC_NDL muhat_glm_OSTRC_NDL pearson_glm_OSTRC_NDL devres_glm_OSTRC_NDL hat_glm_OSTRC_NDL if cook_glm_OSTRC_NDL > 0.05 //subject 182, 181 and 16 has cooks' distance >0.05

//as a sensitivity test use this code as it focuses on observations with cooks D. This due to those observations could change the coefficients and p

//values and this is what I want to check with a sensitivity test

nbreg Total_OSTRC_Alla_Segm_NDL_Counts i.Age_cat AFAQ, exposure(Exposure_Days_Md) vce(cluster Subject) irr

nbreg Total_OSTRC_Alla_Segm_NDL_Counts i.Age_cat AFAQ if Subject!=182 & Subject!=181 & Subject!=16, exposure(Exposure_Days_Md) vce(cluster Subject) irr //does not change the model except for the n.s age cat that goes from 1.03 to 0.92

list Subject muhat_glm_OSTRC_NDL hat_glm_OSTRC_NDL devres_glm_OSTRC_NDL pearson_glm_OSTRC_NDL cook_glm_OSTRC_NDL if hat_glm_OSTRC_NDL > 0.04 | abs(devres_glm_OSTRC_NDL) > 3 | abs(pearson_glm_OSTRC_NDL) > 3 |cook_glm_OSTRC_NDL > 0.05

//subjects in this list that are influential points are: 80, 43, 210, 86,55,81, 185, 181, 182, 16

//(use this code as screening/diagnostic as it shows all types of observations that could be problematic)

nbreg Total_OSTRC_Alla_Segm_NDL_Counts i.Age_cat AFAQ, exposure(Exposure_Days_Md) vce(cluster Subject) irr

nbreg Total_OSTRC_Alla_Segm_NDL_Counts i.Age_cat AFAQ if Subject!=43 & Subject!=80 & Subject!=210 & Subject!=86 & Subject!=55 & Subject!=81 & Subject!=185 & Subject!=181 & Subject!=182 & Subject!=16, exposure(Exposure_Days_Md) vce(cluster Subject) irr

// no greater changes

//CONCLUSION: The model is robust and precise

//c. Specification test-Linktest

//The linktest could indicate that the model is not an appropriate representation of the relationship between the outcome and predictors (misspecification), and furthermore that

//important predictors have been omitted or are represented incorrectly in the model.

nbreg Total_OSTRC_Alla_Segm_NDL_Counts i.Age_cat AFAQ, exposure(Exposure_Days_Md) vce(cluster Subject) irr

linktest //Conclusion:_hatsq=non-significant. This indicates that the model is correctly specified and are the most important finding from this test.

//and _hat is significant: In the literature it is mentioned that _hat should be significant since it is the predicted value for the model.

//In a negative binomial regression Model the pseudo R2 most often are very low (0.05-0.10), see STATA manual or Hilbe 2011. Hilbe discuss the general

//weak predictive value in NB-models, especially with overdispersion and many zeroes.

//AFAQ HAS A HIGH IRR, 4% INCREASE FOR EACH UNIT, IS THE RELATIONSHIP TO THE OUTCOME REALLY LINEAR?

nbreg Total_OSTRC_Alla_Segm_NDL_Counts i.Age_cat AFAQ, exposure(Exposure_Days_Md) vce(cluster Subject) irr

//Testing the linearity of AFAQ with the outcome OSTRC

nbreg Total_OSTRC_Alla_Segm_NDL_Counts c.AFAQ c.AFAQ#c.AFAQ, exposure(Exposure_Days_Md) vce(cluster Subject) irr

//the interaction term is n.s. If the interaction term is n.s the relation is probably linear

nbreg Total_OSTRC_Alla_Segm_NDL_Counts AFAQ, exposure(Exposure_Days_Md) vce(cluster Subject) irr

margins, at(AFAQ=(0(5)45)) predict(ir)

marginsplot, x(AFAQ) ytitle("Predicted incidence rate (IR)") xtitle("AFAQ score") title("Predicted injury rate by AFAQ") ylabel(, angle(horizontal))

//margins and marginsplot suggest a curvilinear relationship

//Testing the linearity for AFAQ in the full model

nbreg Total_OSTRC_Alla_Segm_NDL_Counts i.Age_cat c.AFAQ c.AFAQ#c.AFAQ, exposure(Exposure_Days_Md) vce(cluster Subject) irr

//The interaction term is n,s p=0.18, suggesting linear relationship

nbreg Total_OSTRC_Alla_Segm_NDL_Counts i.Age_cat AFAQ, exposure(Exposure_Days_Md) vce(cluster Subject) irr

margins, at(AFAQ=(0(5)45)) predict(n)

marginsplot, x(AFAQ) ytitle("Predicted number of injuries") xtitle("AFAQ score") title("Predicted number of injuries by AFAQ") ylabel(, angle(horizontal))

//margins and marginsplot suggest an almost linear relationship

nbreg Total_OSTRC_Alla_Segm_NDL_Counts i.Age_cat AFAQ, exposure(Exposure_Days_Md) vce(cluster Subject) irr

margins, at(AFAQ=(0(1)45)) predict(n)

marginsplot, x(AFAQ) ytitle("Predicted number of injuries") xtitle("AFAQ score") title("Predicted number of injuries by AFAQ") ylabel(, angle(horizontal))

//margins and marginsplot suggest an almost linear relationship

*CONCLUSION: It seems that AFAQ´s relation to the outcome is almost linear

*THIS IS THE FINAL MODEL FOR OVERUSE INJURIES FOR THE NON-DOMINANT LEG:

nbreg Total_OSTRC_Alla_Segm_NDL_Counts i.Age_cat AFAQ, exposure(Exposure_Days_Md) vce(cluster Subject) irr

//This model is the model is robust and precise. For OSTRC BMI is not included as an a priori variable as there is no theoretical evidence for this.

//Furthermore, the variable BMI was not linear with the outcome variable on the log scale meaning it had to be squared or categorized. Those maneuverers

//showed to be meaningless due to few values for some categories and to great extrapolation.

//It seems that AFAQ´s relation to the outcome is almost linear

*MODEL BUILDING WITH PURPOSEFUL SELECTION FOR OVERUSE INJURIES: DOMINANT LEG

*STEP 0. Candidate list

//div: P=0.14, stays in the model

//Age: An apriori variable selected from the literature

//Length: P=0.29 excluded due to p value

//BMI: p=0.05 for some variables *** se discussion below

//The SLS: P=0.34

//ADF: P=0.24

//CLAM: P=0.79

//AFAQ: P=0.00

//PSS-14:P=0.35

//PSQI: P=0.47

//GAD-7: P=0.06 for some categories

*STEP 1. Full base Model

//Starting with a full Model and saves AIC and BIC. AIC/BIC is a reference point. If a reduced model has equal or lower AIC/BIC it is a better balance between

//fit and simplicity. If AIC/BIC increases when a variable is eliminated it probably contributed to the Model even though the p-value was low.

nbreg Total_OSTRC_Alla_Segm_DL_Counts i.div i.Age_cat DL_ADF AFAQ i.GAD7_cat2 ,exposure(Exposure_Days_Md) vce(cluster Subject) irr //without BMI

est store full_1_OSTRC_DL

estat ic //AIC=1086   BIC=1121

nbreg Total_OSTRC_Alla_Segm_DL_Counts i.div i.Age_cat i.BMI_cat DL_ADF AFAQ i.GAD7_cat2 ,exposure(Exposure_Days_Md) vce(cluster Subject) irr //with BMI_cat

est store full_1_OSTRC_DL

estat ic //AIC=1071   BIC=1113

```
gen BMI2 = BMI^2

nbreg Total_OSTRC_Alla_Segm_DL_Counts i.div i.Age_cat BMI BMI2  DL_ADF AFAQ i.GAD7_cat2 ,exposure(Exposure_Days_Md) vce(cluster Subject) irr //with BMI squared

est store full_1_OSTRC_DL

estat ic //AIC=1085   BIC=1127

drop BMI2


tab BMI_cat


summarize BMI, detail

histogram BMI, width(1) normal

margins, at(BMI=(18(1)28))

marginsplot, ciopts(lcolor(gs12)) title("Predicerad incidens över BMI") xtitle("BMI") ytitle("Predicerat antal händelser") recast(line)


***Discussion about BMI: (same discussion and decision as for the NDL)

//BMI has very few observations in cat 1, 3 and 4. This means that the extrapolation of a squared term will be meaningless.

//Categorization of the variable will make the model more stable, more meaningful and easier to interpret but

//the great CI shows that the interpretation must be done with caution. As BMI is not an a

//overuse injury it could also be excluded with the arguments above.

//CONLUSION: BMI is taken away from the model after sensitivity testing showing CI for BMI ranging from 0.9 to 18


nbreg Total_OSTRC_Alla_Segm_DL_Counts i.div i.Age_cat DL_ADF AFAQ i.GAD7_cat2 ,exposure(Exposure_Days_Md) vce(cluster Subject) irr //this is the full model to work with

est store full_1_OSTRC_DL

estat ic //AIC=1086   BIC=1121
```

*STEP 2. Wald test and confounding check for each variable

*i.div

//a. Wald test:

//Eliminating and testing one variable at a time with the wald test (LR test is invalid for models with robust vce).

//If p<0.05 the variable contributes significantly and it should stay in the model.

//If p>0.05 the variable does not contribute to the model and could be eliminated.

nbreg Total_OSTRC_Alla_Segm_DL_Counts i.div i.Age_cat DL_ADF AFAQ i.GAD7_cat2 ,exposure(Exposure_Days_Md) vce(cluster Subject) irr

est store full_1_OSTRC_DL

testparm i.div //p=0.79

testparm i.GAD7_cat2 //p=0.37

//b. Confounding-check

//Even though a variable is non-significant, it could be a confounder and should therefore stay in the Model

//The rule of thumb is that a change of 10% or more of the IRR is a confounder (comparing the full Model with the reduces Model)

nbreg Total_OSTRC_Alla_Segm_DL_Counts i.Age_cat DL_ADF AFAQ i.GAD7_cat2 ,exposure(Exposure_Days_Md) vce(cluster Subject) irr //full Model without i.div

est store reduced_1_OSTRC_DL

est table full_1_OSTRC_DL reduced_1_OSTRC_DL, eform b(%9.3f) se(%9.3f) stats(N ll) //no changes >10% is noticed between full_1 and reduced Model. The variable i.div is eliminated

//c. new model without i.div

nbreg Total_OSTRC_Alla_Segm_DL_Counts i.Age_cat DL_ADF AFAQ i.GAD7_cat2, exposure(Exposure_Days_Md) vce(cluster Subject) irr //The new full model

est store full_2_OSTRC_DL

estat ic //AIC=1082 BIC=1110 If AIC and BIC are the same or lower it is a better balance between fit and simplicity which is good. If AIC and BIC rise

//significantly the variable taken away probably contributed to the model even though the p value was poor.

//CONCLUSION: i.div is taken away

*i.GAD7_cat2

```
//a. Wald test

est store full_2_OSTRC_DL

testparm i.GAD7_cat2 //p=0.41


//b. Confounding check

nbreg Total_OSTRC_Alla_Segm_DL_Counts i.Age_cat DL_ADF AFAQ ,
exposure(Exposure_Days_Md) vce(cluster Subject) irr  //full model without i.GAD

est store reduced_2_OSTRC_DL

est table full_2_OSTRC_DL reduced_2_OSTRC_DL, eform b(%9.3f) se(%9.3f) stats(N ll)
//no changes >10% is noticed between full_1 and reduced Model. The variable GAD is
eliminated


//c. new model without GAD-7

nbreg Total_OSTRC_Alla_Segm_DL_Counts i.Age_cat DL_ADF AFAQ ,
exposure(Exposure_Days_Md) vce(cluster Subject) irr  //The new full model

est store full_3_OSTRC_DL

estat ic //AIC=1080, BIC=1101

//CONCLUSION: GAD-7 is taken away


*DL_ADF

//a. Wald test

est store full_3_OSTRC_DL

testparm DL_ADF //p=0.07


//b. Confounding check

nbreg Total_OSTRC_Alla_Segm_DL_Counts i.Age_cat AFAQ ,
exposure(Exposure_Days_Md) vce(cluster Subject) irr  //full model without DL_ADF

est store reduced_3_OSTRC_DL

est table full_3_OSTRC_DL reduced_3_OSTRC_DL, eform b(%9.3f) se(%9.3f) stats(N ll)
//no changes >10% is noticed between full_1 and reduced Model. The variable GAD is
eliminated


//c. model without DL_ADF

nbreg Total_OSTRC_Alla_Segm_DL_Counts i.Age_cat AFAQ ,
exposure(Exposure_Days_Md) vce(cluster Subject) irr  //Model without DL_ADF
```

est store full_4_OSTRC_DL

estat ic //AIC=1082, BIC=1099

//CONCLUSION: The model with or without DL_ADF is about the same regarding robustness. //I can choose to add DL_ADF if its theoretically motivated. I choose to include ADF for now and//see if the variable is sensitive to outliers. If so, i can choose to eliminate it in a later stage

nbreg Total_OSTRC_Alla_Segm_DL_Counts i.Age_cat AFAQ, exposure(Exposure_Days_Md) vce(cluster Subject) irr //The new full model without DL_ADF

nbreg Total_OSTRC_Alla_Segm_DL_Counts i.Age_cat DL_ADF AFAQ, exposure(Exposure_Days_Md) vce(cluster Subject) irr //The new full model with DL_ADF

*STEP 3. Interaction terms

//If the interaction term is significant and/or gives a theoretical reasonable pattern the interaction terms stay in the Model.

//AIC and BIC in the model with interaction terms is compared to the model without interactions, if AIC decreases a lot and the interaction term is theoretical

//reasonable, the terms stay in the Model.

//The new full model is frugal and does not need to be tested for interaction. On the other hand, for TLI we saved BMI as an a priori variable but not for OSTRC.

//BMI and div had an interaction for TLI. This has also been tested for OSTRC as seen below:

nbreg Total_OSTRC_Alla_Segm_DL_Counts i.Age_cat i.div##c.BMI i.div##c.BMI2 AFAQ ,exposure(Exposure_Days_Md) vce(cluster Subject) irr //both BMI and BMI2 are significant

testparm i.div#c.BMI i.div#c.BMI2 //n.s p=0.06

margins div, at(BMI=(15(1)35))

marginsplot

//also checking the interaction between Age and ADF

nbreg Total_OSTRC_Alla_Segm_DL_Counts i.Age_cat i.Age_cat##c.DL_ADF AFAQ, exposure(Exposure_Days_Md) vce(cluster Subject) irr

testparm i.Age_cat##c.DL_ADF //p=0.29

//CONCLUSION: No need to test interactions for this Model and the test for interaction

//between age and adf was non-significant.

nbreg Total_OSTRC_Alla_Segm_NDL_Counts i.Age_cat AFAQ, exposure(Exposure_Days_Md) vce(cluster Subject) irr //The new full model

nbreg Total_OSTRC_Alla_Segm_NDL_Counts i.Age_cat DL_ADF AFAQ, exposure(Exposure_Days_Md) vce(cluster Subject) irr //The new full model with ADF

*STEP 4. Model fit and diagnostic

//a. Analysis of residuals

//Pearson residuals >2 could be deviant and residuals >3 should definitely be scrutinized

//First a visual inspection should be done from scatters, secondly the outliers should be listed and scrutinized regarding their data (data input), thirdly a sensitivity

//analysis should be done to see if the conclusions hold up (model robustness) or not (discussion that the Model is affected by outliers)

//Alt 2: A more complete diagnostic where glm with the nbinom-family is used to receive Pearson residuals/deviance and influential statistics

//OBS! this is just for diagnostics and should not replace nbreg Modelling

glm Total_OSTRC_Alla_Segm_DL_Counts i.Age_cat DL_ADF AFAQ, family(nbinomial `a') link(log) exposure(Exposure_Days_Md) nolog

predict muhat_glm_OSTRC_DL, mu

predict pearson_glm_OSTRC_DL, pearson

predict devres_glm_OSTRC_DL, deviance

predict hat_glm_OSTRC_DL, hat

predict cook_glm_OSTRC_DL, cooksd

scatter pearson_glm_OSTRC_DL muhat_glm_OSTRC_DL, yline(0) title("Pearson residuals (glm)") //Most of the observations are localized around -1 to 2. But there are 13 outliers >2.

list Subject muhat_glm_OSTRC_DL pearson_glm_OSTRC_DL if abs(pearson_glm) > 3 //Subject 20, 81, 165, 262, 172, 216, 225, 168, 180, 208, 55, 206 have residuals >3 and are outliers

list if Subject == 20 | Subject == 81 | Subject == 165 | Subject == 262 | Subject == 172| Subject == 216| Subject == 225| Subject == 168 | Subject == 180| Subject == 208| Subject == 206 | Subject == 55

list Subject Total_OSTRC_Alla_Segm_DL_Counts Age_cat DL_ADF AFAQ pearson_glm_OSTRC_DL if Subject == 20 | Subject == 81 | Subject == 165 | Subject == 262 | Subject == 172| Subject == 216| Subject == 225| Subject == 168 | Subject == 180| Subject == 208| Subject == 206 | Subject == 55

//Conclusion:No data has been recorded incorrectly.

nbreg Total_OSTRC_Alla_Segm_DL_Counts i.Age_cat DL_ADF AFAQ, exposure(Exposure_Days_Md) vce(cluster Subject) irr //Exclusion of outliers should be done on the nbreg model with vce

nbreg Total_OSTRC_Alla_Segm_DL_Counts i.Age_cat DL_ADF AFAQ if Subject!=20 & Subject!=81 & Subject!=165 & Subject!=262 & Subject!=172 & Subject!=216 & Subject!=225 & Subject!=168 & Subject!=180 & Subject!=208 & Subject!=55 & Subject!=206, exposure(Exposure_Days_Md) vce(cluster Subject) irr

//When excluding the outliers ADL gets significant, p=0.05

//Conclusion: When eliminating all outliers at once my general conclusion still holds,

//b. Analysis of deviance residuals

//Deviance residuals: Values greater than 2-3 could indicate deviance.

scatter devres_glm_OSTRC_DL muhat_glm_OSTRC_DL, yline(0) title("Deviance residuals (glm)") //Residuals are in reasonable limits (<3), the curves have no systematic patterns,

//no signs of gross misspecification.

//Leverage grafs (hat values): influential points

//How much an observation affects its prediction, 2p/n is a simple cut off. in my case: di (2*5)/243=.04

//That will say: hat-values greater than 0.04 could be values to check.

scatter hat_glm_OSTRC_DL muhat_glm_OSTRC_DL, title("Leverage vs. Predicted values") ytitle("Leverage (hat)") xtitle("Predicted counts (muhat)") // Nine points are >0.04

list Subject hat_glm_OSTRC_DL muhat_glm_OSTRC_DL if hat_glm_OSTRC_DL > 0.04 //Subject 162 have missing values, 113, 77,80, 109, 42, 6, 217,171,62, 161, 43, 258, 55,

nbreg Total_OSTRC_Alla_Segm_DL_Counts i.Age_cat DL_ADF AFAQ, exposure(Exposure_Days_Md) vce(cluster Subject) irr //Exclusion of influential points should

be done on the nbreg model with vce

nbreg Total_OSTRC_Alla_Segm_DL_Counts i.Age_cat DL_ADF AFAQ if Subject!=113 & Subject!=77 & Subject!=80 & Subject!=109 & Subject!=42 & Subject!=6 & Subject!=217 & Subject!=171 & Subject!=62 & Subject!=161 & Subject!=43 & Subject!=55 & Subject!=258, exposure(Exposure_Days_Md) vce(cluster Subject) irr //exclusion of outliers

//Conclusion: AFAQ is not affected by exclusion of those influential points but DL_ADF is. P value is now//significant, p=0.008 and IRR= 1.06. In other words, ADF is getting stronger

//Cook´s distance (influential points):

//High estimates of pearson/deviance residuals and high estimates of leverage will give a high cooks d.

//Cooks d tells you how much your model would be affected if one observation was taken away.

//Cook’s D has no definitive cut off. A rule of thumb is that values >1.0 in greater data sets are problematic.

//In smaller data sets as this values 0.05-0.1 could be troublesome and values >0.1 should absolutely be scrutinized.

//Another conservative rule is 4/n. In my case n ≈ 243 → 4/243 ≈ 0.017. so, for this material values from 0.02 could be of interest

scatter cook_glm_OSTRC_DL muhat_glm_OSTRC_DL , title("Cook's distance") //4 subjects over 0.05, one of those are >0.2

sort cook_glm_OSTRC_DL

list Subject Total_OSTRC_Alla_Segm_DL_Counts i.Age_cat DL_ADF AFAQ Exposure_Days_Md if _n > _N-5

// No incorrect data found. 0.2 is higher than normal. Subject 182, 197, 206, 258, has high values and 162 has missing values

nbreg Total_OSTRC_Alla_Segm_DL_Counts i.Age_cat DL_ADF AFAQ, exposure(Exposure_Days_Md) vce(cluster Subject) irr

nbreg Total_OSTRC_Alla_Segm_DL_Counts i.Age_cat DL_ADF AFAQ if Subject!=182 & Subject!=197 & Subject!=206 & Subject!=258, exposure(Exposure_Days_Md) vce(cluster Subject) irr

//Same as above, AFAQ is not affected but DL_ADF is. P= 0.003 and IRR=1.06

//Conclusion: The model’s direction is not affected by those influential points, but the variable

//ADF´s p value is sensitive and will be significant with exclusion of those observations. .

//Sort cook's distance (influential points)

sort cook_glm_OSTRC_DL

list Subject cook_glm_OSTRC_DL muhat_glm_OSTRC_DL pearson_glm_OSTRC_DL devres_glm_OSTRC_DL hat_glm_OSTRC_DL in -10/l // Subject 175, 246, 159, 70, 260, 182, 197, 206, 258, are influential points and 162 has missing values.

list Subject cook_glm_OSTRC_DL muhat_glm_OSTRC_DL pearson_glm_OSTRC_DL devres_glm_OSTRC_DL hat_glm_OSTRC_DL if cook_glm_OSTRC_DL > 0.05 //subject 197, 206, 258 has cooks' distance >0.05

//as a sensitivity test use this code as it focuses on observations with cooks D. This due to those observations could change the coefficients and p

//values and this are what I want to check with a sensitivity test

nbreg Total_OSTRC_Alla_Segm_DL_Counts i.Age_cat DL_ADF AFAQ, exposure(Exposure_Days_Md) vce(cluster Subject) irr

nbreg Total_OSTRC_Alla_Segm_DL_Counts i.Age_cat DL_ADF AFAQ if Subject!=197 & Subject!=206 & Subject!=258, exposure(Exposure_Days_Md) vce(cluster Subject) irr //As above, DL_ADF is sensitive to those influential points. P=0.002, IRR=1.06

list Subject muhat_glm_OSTRC_DL hat_glm_OSTRC_DL devres_glm_OSTRC_DL pearson_glm_OSTRC_DL cook_glm_OSTRC_DL if hat_glm_OSTRC_DL > 0.04 | abs(devres_glm_OSTRC_DL) > 3 | abs(pearson_glm_OSTRC_DL) > 3 |cook_glm_OSTRC_NDL > 0.05

//subjects in this list that are outliers and influential points are: 109, 42, 181, 171, 62, 113, 55, 161, 16, 77, 80, 43, 6, 217, 182, 197, 206, 258

//(use this code as screening/diagnostic as it shows all types of observations that could be problematic)

nbreg Total_OSTRC_Alla_Segm_DL_Counts i.Age_cat DL_ADF AFAQ, exposure(Exposure_Days_Md) vce(cluster Subject) irr

nbreg Total_OSTRC_Alla_Segm_DL_Counts i.Age_cat DL_ADF AFAQ if Subject!=109 & Subject!=42 & Subject!=181 & Subject!=171 & Subject!=62 & Subject!=113 & Subject!=55 & Subject!=161 & Subject!=77 & Subject!=16 & Subject!=80 & Subject!=43 & Subject!=6 & Subject!=217 & Subject!=182 & Subject!=197 & Subject!=206 & Subject!=258, exposure(Exposure_Days_Md) vce(cluster Subject) irr

//As above, DL_ADF is affected, p= 0.003 and IRR=1.06

nbreg Total_OSTRC_Alla_Segm_DL_Counts i.Age_cat DL_ADF AFAQ, exposure(Exposure_Days_Md) vce(cluster Subject) irr

nbreg Total_OSTRC_Alla_Segm_DL_Counts i.Age_cat DL_ADF AFAQ if Subject!=258, exposure(Exposure_Days_Md) vce(cluster Subject) irr

list Subject Total_OSTRC_Alla_Segm_DL_Counts Age_cat DL_ADF AFAQ if Subject == 258

sum DL_ADF, detail

sum Total_OSTRC_Alla_Segm_DL_Counts, detail

//it seems that one point, subject 258 drives the model most as she has one of the smallest ADF value (32) and one of the highest OSTRC value (=18)

//CONCLUSION: The model is robust regarding direction but sensitive for precision

//regarding the variable DL_ADF that will be significant when one influential point is

//excluded

//c. Specification test-Linktest

//The linktest could indicate that the model is not an appropriate representation of the relationship between the outcome and predictors (misspecification), and furthermore that

//important predictors have been omitted or are represented incorrectly in the model.

nbreg Total_OSTRC_Alla_Segm_DL_Counts i.Age_cat DL_ADF AFAQ, exposure(Exposure_Days_Md) vce(cluster Subject) irr

linktest //Conclusion:_hatsq=non-significant. This indicates that the model is correctly specified and are the most important finding from this test.

//and _hat is significant: In the literature it is mentioned that _hat should be significant since it is the predicted value for the model.

//In a negative binomial regression Model the pseudo R2 most often are very low (0.05-0.10), see STATA manual or Hilbe 2011. Hilbe discuss the general

//weak predictive value in NB-models, especially with overdispersion and many zeroes.

nbreg Total_OSTRC_Alla_Segm_DL_Counts i.Age_cat DL_ADF AFAQ, exposure(Exposure_Days_Md) vce(cluster Subject) irr //How does the model look without ADF

estat ic // AIC=1082.175, BIC=1099.64, Wald=15.0, Pseudo R2=0.015

linktest //The same results as above

//Conclusion: The model has a stronger overall explanatory effect if I include ADF. .

//AFAQ HAS A HIGH IRR, 4% INCREASE FOR EACH UNIT, IS THE RELATIONSHIP TO THE OUTCOME REALLY LINEAR?

nbreg Total_OSTRC_Alla_Segm_DL_Counts i.Age_cat DL_ADF AFAQ, exposure(Exposure_Days_Md) vce(cluster Subject) irr

//Testing the linearity of AFAQ with the outcome OSTRC

nbreg Total_OSTRC_Alla_Segm_DL_Counts c.AFAQ c.AFAQ#c.AFAQ, exposure(Exposure_Days_Md) vce(cluster Subject) irr

//the interaction term is n.s. If the interaction term is n.s the relation is probably linear

nbreg Total_OSTRC_Alla_Segm_DL_Counts AFAQ, exposure(Exposure_Days_Md) vce(cluster Subject) irr

margins, at(AFAQ=(0(5)45)) predict(ir)

marginsplot, x(AFAQ) ytitle("Predicted incidence rate (IR)") xtitle("AFAQ score") title("Predicted injury rate by AFAQ") ylabel(, angle(horizontal))

//margins and marginsplot suggest an almost linear relationship

//Testing the linearity for AFAQ in the full model

nbreg Total_OSTRC_Alla_Segm_DL_Counts i.Age_cat DL_ADF c.AFAQ c.AFAQ#c.AFAQ, exposure(Exposure_Days_Md) vce(cluster Subject) irr

//The interaction term is n,s p=0.38, suggesting linear relationship

nbreg Total_OSTRC_Alla_Segm_DL_Counts i.Age_cat DL_ADF AFAQ, exposure(Exposure_Days_Md) vce(cluster Subject) irr

margins, at(AFAQ=(0(5)45)) predict(n)

marginsplot, x(AFAQ) ytitle("Predicted number of injuries") xtitle("AFAQ score") title("Predicted number of injuries by AFAQ") ylabel(, angle(horizontal))

//margins and marginsplot an almost linear relationship

nbreg Total_OSTRC_Alla_Segm_DL_Counts i.Age_cat DL_ADF c.AFAQ c.AFAQ#c.AFAQ, exposure(Exposure_Days_Md) vce(cluster Subject) irr

margins, at(AFAQ=(0(1)45)) predict(n)

marginsplot, x(AFAQ) ytitle("Predicted number of injuries") xtitle("AFAQ score") title("Predicted number of injuries by AFAQ") ylabel(, angle(horizontal))

//margins and marginsplot an almost linear relationship

*CONCLUSION: It seems that AFAQ´s relation to the outcome is almost linear

*THIS IS THE FINAL MODEL FOR OVERUSE INJURIES FOR THE DOMINANT LEG:

nbreg Total_OSTRC_Alla_Segm_DL_Counts i.Age_cat DL_ADF AFAQ, exposure(Exposure_Days_Md) vce(cluster Subject) irr

//Notes: This model is robust regarding direction for both variables but DL_ADF is sensitive for one outlier (subject 258)who have one of the smallest ADF values

// and one of the highest OSTRC values. Exclusion of these subjects changes the precision of the variable ADF to be significant.

//For OSTRC, BMI is not included as an a priori variable as there is no theoretical evidence for this.

//Furthermore, the variable BMI was not linear with the outcome variable on the log scale meaning it had to be squared or categorized. Those maneuverers

//showed to be meaningless due to few values for some categories and to great extrapolation.

//It seems that AFAQ´s relation to the outcome is almost linear

//Compared with the simpler model, the model including DL_ADF showed stronger overall explanatory power, reflected by a higher Wald χ^2 (21 vs. 14)

//and a modest improvement in pseudo-R^2. This indicates that the full model captures more of the variation in injury counts, even though individual

//coefficients (e.g., DL_ADF) may be only borderline significant. Together with a slightly lower AIC, these results support retaining the fuller model.”

****

*Sensitivity analyses for injury problems: Exposure

****

nbreg Total_OSTRC_Alla_Segm_NDL_Counts i.Age_cat AFAQ, exposure(Exposure_Days_Md) vce(cluster Subject) irr //Full model

nbreg Total_OSTRC_Alla_Segm_NDL_Counts i.Age_cat AFAQ, exposure(exposure_0) vce(cluster Subject) irr

nbreg Total_OSTRC_Alla_Segm_NDL_Counts i.Age_cat AFAQ, exposure(exposure_15) vce(cluster Subject) irr

nbreg Total_OSTRC_Alla_Segm_NDL_Counts i.Age_cat AFAQ, exposure(exposure_cca) vce(cluster Subject) irr

nbreg Total_OSTRC_Alla_Segm_DL_Counts i.Age_cat DL_ADF AFAQ, exposure(Exposure_Days_Md) vce(cluster Subject) irr //Full model

nbreg Total_OSTRC_Alla_Segm_DL_Counts i.Age_cat DL_ADF AFAQ, exposure(exposure_0) vce(cluster Subject) irr

nbreg Total_OSTRC_Alla_Segm_DL_Counts i.Age_cat DL_ADF AFAQ, exposure(exposure_15) vce(cluster Subject) irr

nbreg Total_OSTRC_Alla_Segm_DL_Counts i.Age_cat DL_ADF AFAQ, exposure(exposure_cca) vce(cluster Subject) irr

****

***Sensitivity analyses for injury problems: Central lumbar problems**

****

gen Tot_OSTRC_DL_Counts_NoLumb = Total_OSTRC_Alla_Segm_DL_Counts - Total_OSTRC_DL_Länd_Counts

sum Total_OSTRC_Alla_Segm_DL_Counts Total_OSTRC_DL_Länd_Counts Tot_OSTRC_DL_Counts_NoLumb

di 3.016393-.7459016

assert Tot_OSTRC_DL_Counts_NoLumb >= 0

count if Total_OSTRC_DL_Länd_Counts > 0

nbreg Total_OSTRC_Alla_Segm_DL_Counts i.Age_cat DL_ADF AFAQ, exposure(Exposure_Days_Md) vce(cluster Subject) irr

nbreg Tot_OSTRC_DL_Counts_NoLumb i.Age_cat DL_ADF AFAQ, exposure(Exposure_Days_Md) vce(cluster Subject) irr

gen Tot_OSTRC_NDL_Counts_NoLumb = Total_OSTRC_Alla_Segm_NDL_Counts - Total_OSTRC_NDL_Länd_Counts

sum Total_OSTRC_Alla_Segm_NDL_Counts Total_OSTRC_NDL_Länd_Counts Tot_OSTRC_NDL_Counts_NoLumb

di 2.709016 - .7581967

assert Tot_OSTRC_NDL_Counts_NoLumb >= 0

count if Total_OSTRC_NDL_Länd_Counts > 0

nbreg Total_OSTRC_Alla_Segm_NDL_Counts i.Age_cat AFAQ, exposure(Exposure_Days_Md) vce(cluster Subject) irr

nbreg Tot_OSTRC_NDL_Counts_NoLumb i.Age_cat AFAQ, exposure(Exposure_Days_Md) vce(cluster Subject) irr

summarize Total_OSTRC_DL_Länd_Counts

display r(sum) //=182

summarize Total_OSTRC_NDL_Länd_Counts

display r(sum) //=185

****

*Sensitivity analyses time-loss injuries: Exposure

****

nbreg TLI_Alla_Segment_NDL i.Age_cat i.div NDL_ADF NDL_Nm3BWm_100,exposure(Exposure_Days_Md) vce(cluster Subject) irr //full model

nbreg TLI_Alla_Segment_NDL i.Age_cat i.div NDL_ADF NDL_Nm3BWm_100,exposure(exposure_0) vce(cluster Subject) irr

nbreg TLI_Alla_Segment_NDL i.Age_cat i.div NDL_ADF NDL_Nm3BWm_100,exposure(exposure_15) vce(cluster Subject) irr

nbreg TLI_Alla_Segment_NDL i.Age_cat i.div NDL_ADF NDL_Nm3BWm_100,exposure(exposure_cca) vce(cluster Subject) irr

nbreg TLI_Alla_Segment_DL i.Age_cat##c.DL_Nm3BWm_100 AFAQ, exposure(Exposure_Days_Md) vce(cluster Subject) irr //Full modell

nbreg TLI_Alla_Segment_DL i.Age_cat##c.DL_Nm3BWm_100 AFAQ, exposure(exposure_0) vce(cluster Subject) irr

nbreg TLI_Alla_Segment_DL i.Age_cat##c.DL_Nm3BWm_100 AFAQ, exposure(exposure_15) vce(cluster Subject) irr

nbreg TLI_Alla_Segment_DL i.Age_cat##c.DL_Nm3BWm_100 AFAQ, exposure(exposure_cca) vce(cluster Subject) irr
