# Supplementary material for: How are biomechanical and psychosocial factors associated with injuries across one season in Swedish elite and sub-elite female soccer players?
Source: BMC Sports Sci Med Rehabil. 2026 Sep 17;18:435. doi: 10.1186/s13102-026-02084-y (PMC13599122; doi:10.1186/s13102-026-02084-y)
Supplement: Supplementary file 3 [file 13102_2026_2084_MOESM3_ESM.pdf]

Additional file 3

Additional file 3. Univariate analyses of an injury problem in the dominant and non-dominant leg

	Injury problems for the lower back and lower extremities: non-dominant leg				Injury problems for the lower back and lower extremities: dominant leg			
**Variable**	[a]n	[b]IRR	[c]95% CI	[d]p-value	[a]n	[b]IRR	[c]95% CI	[d]p-value
**Division**	244	0.97	0.81-1.1.15	0.70	244	0.89	0.76-1.04	0.14
Div. 1	89	1			89	1		
Div. 2	49	1.06	0.70-1.63	0.78	49	1.06	0..72-1.56	0.78
Div. 3	106	0.94	0.66-1.33	0.71	106	0.79	0.58-1.09	0.15
**Age category**	244	1.19	0.96-1.50	0.12	244	1.16	0.95-1.41	0.14
16-19 years	83	1			83	1		
20-24 years	94	1.19	0.81-1.74	0.38	94	1.18	0.83-1.68	0.35
25-39 years	67	1.41	0.92-2.15	0.12	67	1.34	0.91-1.99	0.14
**Length (m)**	244	0.99	0.96-1.02	0.43	244	0.99	0.96-1.01	0.29
**BMI category[e]**	241	0.72	0.41-1.28	0.27	241	0.93	0.62-1.40	0.74
≥18.5 & ≤24.9	223	7.81	1.91-31.90	**0.00***	223	4.13	1.02-16.83	**0.05***
≥25.0 & ≤29.9	15	4.40	0.89-21.63	0.07	15	3.25	0.73-14.40	0.12
≥30.0	1	5.89	1.46-23.81	**0.01***	1	4.41	1.09-17.81	**0.04***
**The Single Leg Squat**	244				244			
Pass	133	1			75	1		
Fail	111	0.78	0.56-1.06	0.11	169	0.69	0.33-1.48	0.34
**Ankle dorsiflexion[f]**	244	1.00	0.96-1.05	0.92	244	1.03	0.98-1.07	0.24
**Hip strength[g]**	243	1.00	0.99-1.01	0.70	242	1.00	0.99-1.01	0.79
**AFAQ[h]**	243	1.04	1.02-1.06	**0.00***	243	1.04	1.02-1.07	**0.00***
**PSS-14[i]**	243	1.01	0.97-1.06	0.57	243	1.02	0.98-1.06	0.35
**PSQI[j]**	243				243			
Good sleepers, ≤5 points	152	1			152	1		
Poor sleepers, ≥6 points	91	0.97	0.70-1.34	0.85	91	0.90	0.66-1.21	0.47
**GAD-7[k]**	243	1.21	0.97-1.52	0.10	243	1.15	0.92-1.44	0.22
No anxiety	104	1			104	1		
Mild anxiety ≥5 points	96	1.22	0.86-1.72	0.26	96	1.34	0.99-1.83	0.06
Moderate/severe anxiety ≥10 points	43	1.46	0.92-2.33	0.11	43	1.21	0.75-1.97	0.44

***Denotes a statistically significant differences at p≤0.05;**
[a]**n**: total sample; [b]**IRR:** Injury Rate Ratio; [c]**95 % CI:** 95 % confidence interval; [d]**p-value:** probability value; [e]**BMI:** Body Mass Index; [f]**Ankle dorsiflexion**: measured with the Weight Bearing Dorsiflexion Lunge Test (WBLT) and calculated with a trigonometric dorsiflexion angle (TA); [g]**Hip strength:** Side-Lying Clamshell (CLAM), body-sized independent measurement; Newton (N)*length of femur (m)/ (Body weight (kg)*height (m)) *100; [h]**AFAQ**: Athletic Fear Avoidance Questionnaire; [i]**PSS-14**: Perceived Stress Scale 14-item instrument; [j]**PSQI**: Pittsburgh Sleep Quality Index; [k]**GAD-7:** Generalized Anxiety Disorder 7-item scale.
