# Supplementary material for: How are biomechanical and psychosocial factors associated with injuries across one season in Swedish elite and sub-elite female soccer players?
Source: BMC Sports Sci Med Rehabil. 2026 Sep 17;18:435. doi: 10.1186/s13102-026-02084-y (PMC13599122; doi:10.1186/s13102-026-02084-y)
Supplement: Supplementary file 4 [file 13102_2026_2084_MOESM4_ESM.pdf]

Additional file 4

Additional file 4. Univariate analyses of a time-loss injury in the dominant and non-dominant leg

	Time-loss injury for the lower back and lower extremities: non-dominant leg				Time-loss injury for the lower back and lower extremities: dominant leg			
Variable	[a]n	[b]IRR	[c]95% CI	[d]p-value	[a]n	[b]IRR	[c]95% CI	[d]p-value
**Division**	244	1.03	0.73-1.47	0.86	244	0.85	0.56-1.29	0.44
Div. 1	89	1			89	1		
Div. 2	49	2.64	1.11-6.27	**0.03***	49	0.75	0.27-2.08	0.58
Div. 3	106	1.09	0.49-2.40	0.84	106	0.72	0.32-1.65	0.44
**Age category**	244	0.97	0.63-1.05	0.89	244	1.10	0.69-1.76	0.69
16-19 years	83	1			83	1		
20-24 years	94	1.12	0.49-2.57	0.80	94	1.05	0.43-2.55	0.92
25-39 years	67	0.93	0.38-2.30	0.88	67	1.21	0.48-3.07	0.68
**Length (m)**	244	1.02	0.96-1.09	0.45	244	0.97	0.90-1.04	0.36
**BMI[e]**	243	1.08	0.91-1.29	0.39	243	1.06	0.87-1.29	0.56
**The Single Leg Squat**	244				244			
Pass	133	1			75	1		
Fail	111	1.10	0.54-2.24	0.80	169	0.69	0.33-1.48	0.34
**Ankle dorsiflexion[f]**	244	0.91	0.83-0.99	**0.04***	244	0.99	0.91-1.08	0.84
**Hip strength[g]**	243	0.98	0.97-0.99	**0.02***	242	0.97	0.95-0.99	**0.00***
**AFAQ[h]**	243	1.03	0.99-1.08	0.20	243	1.09	1.03-1.15	**0.00***
**PSS-14[i]**	243	1.0	0.90-1.10	0.92	243	1.05	0.92-1.20	0.44
**PSQI[j]**	243				243			
Good sleepers, ≤5 points	152	1			152	1		
Poor sleepers, ≥6 points	91	0.98	0.47-2.05	0.95	91	1.32	0.63-2.80	0.46
**GAD-7[k]**	243	1.35	0.85-2.14	0.21	243	1.69	1.06-2.69	**0.03***
No anxiety	104	1			104	1		
Mild anxiety ≥5 points	96	1.41	0.66-3.00	0.38	96	2.55	1.04-6.30	**0.04***
Moderate/severe anxiety ≥10 points	43	1.79	0.68-4.74	0.24	43	2.63	0.93-7.6	0.07

***Denotes a statistically significant p value at p≤0.05**

[a]**n**: total sample; [b]**IRR:** Injury Rate Ratio; [c]**95 % CI:** 95 % confidence interval; [d]**p-value:** probability value; [e]**BMI:** Body Mass Index; [f]**Ankle dorsiflexion**: measured with the Weight Bearing Dorsiflexion Lunge Test (WBLT) and calculated with a trigonometric dorsiflexion angle (TA); [g]**Hip strength:** Side-Lying Clamshell (CLAM), body-sized independent measurement; Newton (N)*length of femur (m)/(Body weight (kg)*height (m)) *100; [h]**AFAQ**: Athletic Fear Avoidance Questionnaire; [i]**PSS-14**: Perceived Stress Scale 14-item instrument; [j]**PSQI**: Pittsburgh Sleep Quality Index; [k]**GAD-7:** Generalized Anxiety Disorder 7-item scale.
