# Supplementary material for: How are biomechanical and psychosocial factors associated with injuries across one season in Swedish elite and sub-elite female soccer players?
Source: BMC Sports Sci Med Rehabil. 2026 Sep 17;18:435. doi: 10.1186/s13102-026-02084-y (PMC13599122; doi:10.1186/s13102-026-02084-y)

Additional file 5

Linear combination of coefficients-Lincom

1. Multivariate model for time-loss injury dominant leg

```
Negative binomial regression                    Number of obs     =        241
                                                Wald chi2(6)      =      29.10
Dispersion           = mean                     Prob > chi2       =     0.0001
Log pseudolikelihood = -108.92685               Pseudo R2         =     0.0874

                                   (Std. Err. adjusted for 241 clusters in Subject)
-----------------------------------------------------------------------------------------
                        |               Robust
    TLI_Alla_Segment_DL |        IRR   Std. Err.      z    P>|z|     [95% Conf. Interval]
------------------------+----------------------------------------------------------------
                Age_cat |
                 20-24  |   .0142898   .0254985    -2.38   0.017     .0004327    .4719495
                   >24  |   .4447219   .8177352    -0.44   0.659     .0121042    16.33957
                        |
          DL_Nm3BWm_100 |   .9502777   .0159705    -3.03   0.002      .919486    .9821006
                        |
Age_cat#c.DL_Nm3BWm_100 |
                 20-24  |   1.047451   .0217321     2.23   0.025     1.005712    1.090923
                   >24  |   1.011272   .0228846     0.50   0.620     .9673997    1.057135
                        |
                   AFAQ |   1.082591   .0259935     3.31   0.001     1.032824    1.134755
                  _cons |   .0087134    .012506    -3.30   0.001      .000523    .1451694
   ln(Exposure_Days_Md) |          1  (exposure)
------------------------+----------------------------------------------------------------
               /lnalpha |  -.2972089   1.153127                     -2.557297    1.962879
------------------------+----------------------------------------------------------------
                  alpha |   .7428888   .8566452                       .077514    7.119793
-----------------------------------------------------------------------------------------
Note: Estimates are transformed only in the first equation.
Note: _cons estimates baseline incidence rate.
```

2. Post-estimation commands used for interpretation of the interaction model

a. The protective effect of hip strength in the age category 16-19 years

. lincom DL_Nm3BWm_100, eform //IRR=0.95, p=0.95

```
 ( 1)  [TLI_Alla_Segment_DL]DL_Nm3BWm_100 = 0

------------------------------------------------------------------------------
TLI_Alla~_DL |     exp(b)   Std. Err.      z    P>|z|     [95% Conf. Interval]
-------------+----------------------------------------------------------------
         (1) |   .9502777   .0159705    -3.03   0.002      .919486    .9821006
------------------------------------------------------------------------------
```

b. The protective effect of hip strength in the age category 20-24 years

. lincom DL_Nm3BWm_100 + 2.Age_cat#c.DL_Nm3BWm_100, eform //IRR=0.995, P=0.71

```
 ( 1)  [TLI_Alla_Segment_DL]DL_Nm3BWm_100 + [TLI_Alla_Segment_DL]2.Age_cat#c.DL_Nm3BWm_100 = 0

------------------------------------------------------------------------------
TLI_Alla~_DL |     exp(b)   Std. Err.      z    P>|z|     [95% Conf. Interval]
-------------+----------------------------------------------------------------
         (1) |   .9953695   .0122995    -0.38   0.707     .9715526     1.01977
------------------------------------------------------------------------------
```

c. The protective effect of hip strength in the age category >24 years

. lincom DL_Nm3BWm_100 + 3.Age_cat#c.DL_Nm3BWm_100, eform //IRR=0.96, P=0.008

```
 ( 1)  [TLI_Alla_Segment_DL]DL_Nm3BWm_100 + [TLI_Alla_Segment_DL]3.Age_cat#c.DL_Nm3BWm_100 = 0

------------------------------------------------------------------------------
TLI_Alla~_DL |     exp(b)   Std. Err.      z    P>|z|     [95% Conf. Interval]
-------------+----------------------------------------------------------------
         (1) |   .9609897   .0144722    -2.64   0.008     .9330392    .9897774
------------------------------------------------------------------------
```

d. Predicted injury counts across hip strength values within each age category

```
. margins Age_cat, at(DL_Nm3BWm_100=(70(5)110))

Predictive margins                              Number of obs     =        241
Model VCE    : Robust

Expression   : Predicted number of events, predict()

1._at        : DL_Nm3BW~100    =          70

2._at        : DL_Nm3BW~100    =          75

3._at        : DL_Nm3BW~100    =          80

4._at        : DL_Nm3BW~100    =          85

5._at        : DL_Nm3BW~100    =          90

6._at        : DL_Nm3BW~100    =          95

7._at        : DL_Nm3BW~100    =         100

8._at        : DL_Nm3BW~100    =         105

9._at        : DL_Nm3BW~100    =         110

------------------------------------------------------------------------------
             |            Delta-method
             |     Margin   Std. Err.      z    P>|z|     [95% Conf. Interval]
-------------+----------------------------------------------------------------
 _at#Age_cat |
      1#<20  |   .4072959   .1210963     3.36   0.001     .1699515    .6446404
    1#20-24  |   .1493834   .0713674     2.09   0.036     .0095058     .289261
      1#>24  |    .396982    .150851     2.63   0.008     .1013196    .6926445
      2#<20  |   .3156187    .092647     3.41   0.001     .1340339    .4972036
    2#20-24  |   .1459567   .0640442     2.28   0.023     .0204323    .2714811
      2#>24  |   .3253602   .1096247     2.97   0.003     .1104997    .5402206
      3#<20  |   .2445769    .076587     3.19   0.001     .0944692    .3946847
    3#20-24  |   .1426086   .0578338     2.47   0.014     .0292564    .2559608
```

3#>24	.2666601	.081753	3.26	0.001	.1064272	.4268929
4#<20	.1895257	.0667578	2.84	0.005	.0586828	.3203686
4#20-24	.1393373	.0528798	2.63	0.008	.0356948	.2429798
4#>24	.2185504	.0640168	3.41	0.001	.0930797	.3440211
5#<20	.1468659	.0595134	2.47	0.014	.0302217	.2635101
5#20-24	.1361411	.049317	2.76	0.006	.0394815	.2328006
5#>24	.1791204	.0534208	3.35	0.001	.0744176	.2838233
6#<20	.1138082	.0531941	2.14	0.032	.0095498	.2180667
6#20-24	.1330181	.0472273	2.82	0.005	.0404544	.2255819
6#>24	.1468043	.0472139	3.11	0.002	.0542667	.2393418
7#<20	.0881914	.0472334	1.87	0.062	-.0043843	.1807671
7#20-24	.1299668	.0465931	2.79	0.005	.038646	.2212876
7#>24	.1203185	.0432579	2.78	0.005	.0355346	.2051023
8#<20	.0683406	.0415332	1.65	0.100	-.013063	.1497443
8#20-24	.1269855	.0472793	2.69	0.007	.0343198	.2196512
8#>24	.0986111	.0402288	2.45	0.014	.0197641	.1774581
9#<20	.052958	.0361502	1.46	0.143	-.0178952	.1238112
9#20-24	.1240726	.0490608	2.53	0.011	.0279152	.22023
9#>24	.0808201	.0374689	2.16	0.031	.0073825	.1542577

e. A plot of the linear relation of age and hip strength for different age categories

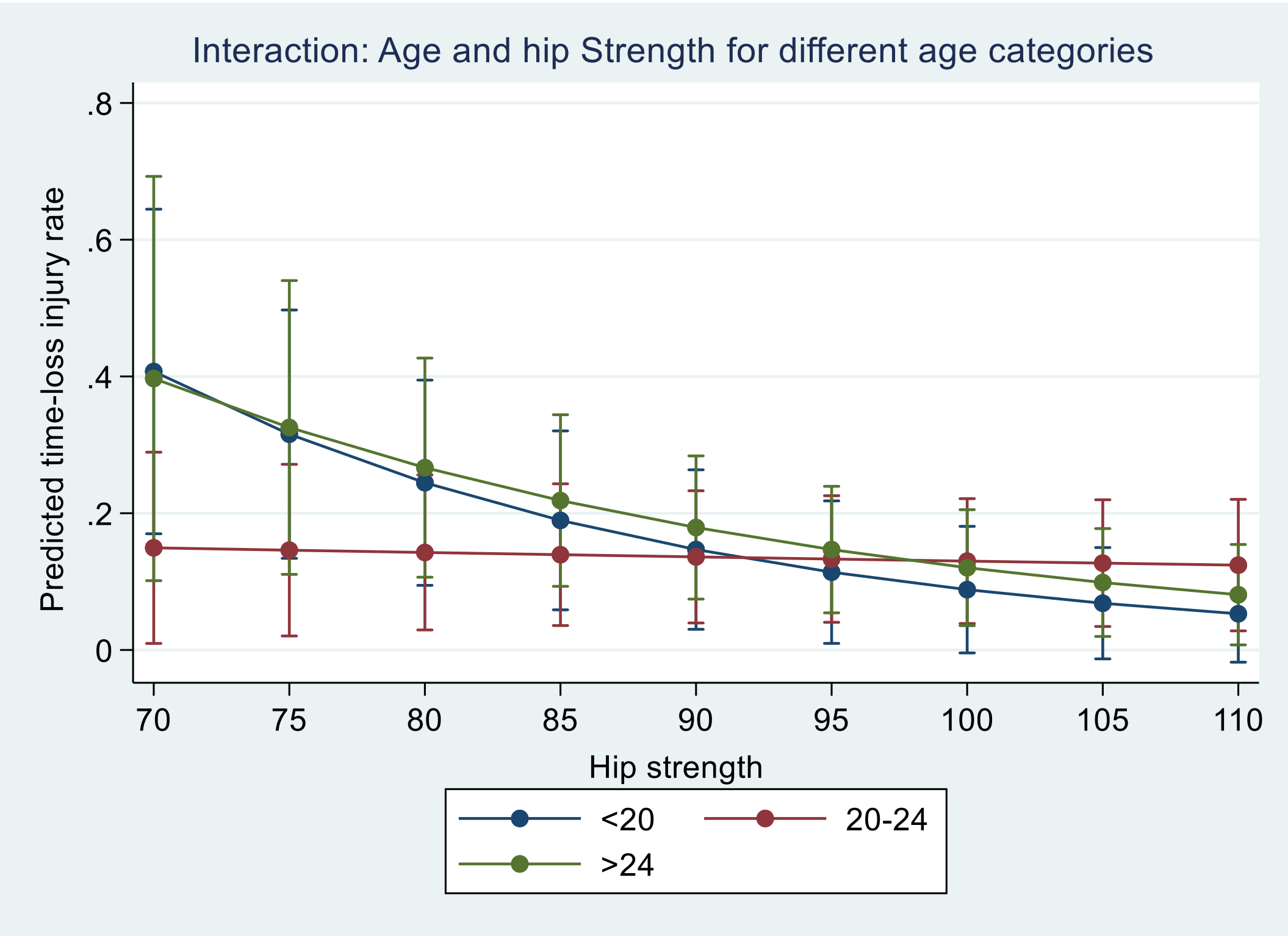

Supplement: Supplementary file 5 [file 13102_2026_2084_MOESM5_ESM.pdf]
