# Supplementary material for: How are biomechanical and psychosocial factors associated with injuries across one season in Swedish elite and sub-elite female soccer players?
Source: BMC Sports Sci Med Rehabil. 2026 Sep 17;18:435. doi: 10.1186/s13102-026-02084-y (PMC13599122; doi:10.1186/s13102-026-02084-y)
Supplement: Supplementary file 6 [file 13102_2026_2084_MOESM6_ESM.pdf]

Sensitivity analyses regarding exposure

IRR-30: Incidence rate ratio with original exposure used in the study. In case of a non-response regarding the OSTRC questionnaire for one month, 30 days of absence were registered and used to adjust the exposure.

IRR-15: Incidence rate ratio with the assignment of 15 days of absence in case of a non-response regarding the OSTRC questionnaire.

IRR-0: Incidence rate ratio with the assignment of zero days of absence in case of a non-response regarding the OSTRC questionnaire.

IRR-CCA: Complete case analysis, all participants that once during the 10 months of follow up had a non-response are excluded from the study. In total 91 person (33%) had at least one non-response during the study.

Table 1. Time-loss injuries, non-dominant leg

	**Division 2 vs 1**	**Ankle dorsiflexion**	**Hip strength**
**IRR-30 (p-value, 95% CI)**	2.75 (**0.01*,** 1.23-6.16)	0.92 (**0.02***,0.84-0.99)	0.98 (**0.00*,**0.96-0.99)
**IRR-15 (p-value, 95% CI)**	2.44 (**0.02*,** 1.19-5.00)	0.93 (**0.05***, 0.87-0.99)	0.98 (**0.00***, 0.97-0.99)
**IRR-0 (p-value, 95% CI)**	2.25 (**0.02***, 1.11-4.56)	0.93 (**0.05***, 0.87-0.99)	0.98 (**0.00***, 0.97-0.99)
**IRR-CCA (p-value, 95% CI)**	1.62 (0.34, 0.60-4.37)	0.96 (0.34, 0.88-1.05)	0.98 (**0.00***, 0.97-0.99)
***Denotes a statistically significant p values at p≤0.05**<br>**IRR:** Incidence rate ratio**; 95% CI:** 95% Confidence interval**; p-value:** Probability value.			

Table 2. Time-loss injuries, dominant leg

	**Hip strength**	**Interaction effect 20-24 years**	**AFAQ**
**IRR-30 (p-value, 95% CI)**	0.95 (**0.00***, 0.92-0.98)	1.05 (**0.03***, 1.01-1.09)	1.08 (**0.00***, 1.03-1.14)
**IRR-15 (p-value, 95% CI)**	0.96 (**0.02***, 0.93-0.99)	1.03 (0.11, 0.99-1.08)	1.07 (**0.00***, 1.03-1.11)
**IRR-0 (p-value, 95% CI)**	0.96 (**0.01***, 0.93-0.99)	1.04 (0.10, 0.99-1.08)	1.07 (**0.00***, 1.03-1.11)
**IRR-CCA (p-value, 95% CI)**	0.97 (0.13, 0.93-1.01)	1.02 (0.49, 0.97-1.07)	1.08 (**0.00***, 1.03-1.14)
***Denotes a statistically significant p values at p≤0.05**<br>**IRR:** Incidence rate ratio**; 95% CI:** 95% Confidence interval**; p-value:** Probability value.			

Table 3. Injury problems, non-dominant leg

	**AFAQ**
**IRR-30 (p-value, 95% CI)**	1.04 (**0.00***, 1.02-1.06)
**IRR-15 (p-value, 95% CI)**	1.03 (**0.01***, 1.01-1.06)
**IRR-0 (p-value, 95% CI)**	1.03 (**0.00***, 1.01-1.06)
**IRR-CCA (p-value, 95% CI)**	1.02 (0.09, 0.99-1.05)
***Denotes a statistically significant p values at p≤0.05**<br>**IRR:** Incidence rate ratio**; 95% CI:** 95% Confidence interval**;**<br>**p-value:** Probability value.	

Table 4. Injury problems, dominant leg

	**Ankle dorsiflexion**	**AFAQ**
**IRR-30 (p-value, 95% CI)**	1.04 (0.07, 0.99-1.08)	1.04 (**0.00***, 1.02-1.07)
**IRR-15 (p-value, 95% CI)**	1.04 (0.12, 0.99-1.08)	1.03 (**0.00***, 1.02-1.06)
**IRR-0 (p-value, 95% CI)**	1.04 (0.10, 0.99-1.08)	1.04 (**0.00***, 1.01-1.06)
**IRR-CCA (p-value, 95% CI)**	1.06 (**0.01***, 1.02-1.11)	1.03 (**0.04***, 1.00-1.06)

***Denotes a statistically significant p values at p≤0.05**
IRR: Incidence rate ratio**; 95% CI:** 95% Confidence interval**; p-value:** Probability value.
