# Supplementary material for: How are biomechanical and psychosocial factors associated with injuries across one season in Swedish elite and sub-elite female soccer players?
Source: BMC Sports Sci Med Rehabil. 2026 Sep 17;18:435. doi: 10.1186/s13102-026-02084-y (PMC13599122; doi:10.1186/s13102-026-02084-y)
Supplement: Supplementary file 7 [file 13102_2026_2084_MOESM7_ESM.pdf]

Additional file 7

Guidelines for research documentation and data management at Karolinska Institutet

Document number 1-20/2021

Valid from 2021-01-26

NOTE: This is a translation of the Swedish version (Riktlinjer för forskningsdokumentation och datahantering på KI). In the event of any discrepancy between the versions, the Swedish version constitutes the official decision and the Swedish wording will prevail.

Karolinska Institutet

Content

1 Introduction 3
2 Purpose 3
3 Responsibility 3
4 Permits and agreements 4
4.1 Personal information 4
5 Planing and documentation 4
5.1 What should be documented? 5
6 Data management 5
6.1 Active data 6
6.2 Storage, sharing and archiving of data 6
6.3 Making research data available 7
7 Research support 7
8 Links 7

**Document number:** 1-20/2021	**Document number for previous version: -**	**Decided:** 2021-01-26	**Valid:** Until further notice
**Decided by:** Committee for research (Kommittén för forskning)		**Dokument type:** Guidelines	
**Administrative division / unit:** Research Support Office/ Research Data Office		**Prepared in consultation with:** Working group appointed by the Committee for research, the Research Data Office, and the Legal Unit	
**Revision with respect of:** New document			

1 Introduction

Good research practice presupposes that the entire research process, from thought and idea, through collection and analysism to results and dissemination of research results, is documented and archived in a correct manner. It is also important that all research data collected or produced within a research project is handled correctly throughout the process.

Research is surrounded by regulations (laws, regulations, various codes and recommendations) that are there to increase and ensure good scientific quality and to protect researchers, experimental animals, employees and not least the integrity and objectivity of research. These regulations also regulate how research is to be documented.

These guidelines complement the governing documents "Guidelines for research at KI" and "Coherent quality system at KI", that more comprehensive regulate research at Karolinska Institutet (KI).

2 Purpose

Guidelines for research documentation and data management at KI summarize how research should be documented and how research data should be handled throughout the life cycle for data and are aimed for everyone who performs research at KI.

3 Responsibility

KI is responsible for the processing of the research data that researchers at KI collect, the data that is generated and all associated documentation within a research project, even if the project funding is external. This means that KI must protect data from, among other things, unauthorized access and that KI is responsible for confidentiality assessment and possible disclosure of data in accordance with applicable regulations.

For research projects conducted in collaboration with other higher education institutions or actors, it is important that agreements that regulate collaboration and responsibility regarding research data and documentation are drawn up. At each department, the head of department has the ultimate responsibility for ensuring that the research, which includes documentation, data management, data storage, publication and archiving, takes place in accordance with current regulations.

The individual researcher must follow good research practice and the rules and guidelines that govern research. If a researcher leaves KI and wants continued access to data, the researcher, together with, for example, the new university, can request that data be disclosed. Data disclosure in some cases can be regulated by agreement.

4 Permits and agreements

A number of different permits and agreements are usually needed before a project can start and some of these agreements also regulate data management within the project.

Requirements for how data is to be handled may occur in ethics permits, biobank agreements, collaboration and assignment agreements, consortium agreements (usually in international collaborations), personal data processing agreements or in financiers' requirements for making documentation and data available in projects they finance.

As a researcher, it is important to know which agreements are needed and what these regulate. Contract review is usually done by KI's lawyers.

Permits, funders' terms and agreements must be recorded and archived in accordance with KI's document management plan and should easily be traced to the research that is conducted.

4.1 Personal information

Personal information is information that can be directly or indirectly traced to a living person. Sensitive personal data refers to information about race or ethnic origin, political opinions, religious or philosophical beliefs, trade union membership, health, a person's sexual life or sexual orientation, genetic data and biometric data that uniquely identify an individual.

Research projects that collect and process personal data must comply with the requirements of the General Data Protection Regulation (GDPR) and be notified to KI's data protection officer via a web form (registeranmalan.ki.se).

All processing of personal data in research must take place in accordance with current regulations for data protection and KI's guidelines for information security. When requesting the disclosure of data containing personal data, a confidentiality assessment must be made.

5 Planing and documentation

All research conducted at KI must be documented, either in Swedish or English. Employees and others must be able to follow and review the research. It is important that the entire research process, from idea and planning to results and conclusions, is described clearly and in detail.

Keeping research organized through documentation is central for being able to account for the research process in accordance with good research practice. Research documentation is also needed to ensure traceability, reproducibility and reusability, protect copyright and patents and facilitate collaborations.

Research documentation at KI must be done electronically in approved systems, which is why KI provides a central ELN system (electronic notebook).

5.1 What should be documented?

Research documentation shall include the intellectual and practical aspects of the research, and, when necessary, refer to the administrative documents, such as funding, permits and agreements, relating to the research.

Much of the comprehensive information can be given in a project description with an associated data management plan which is then supplemented continuously with active documentation of the research process where the methods and data management are described and any changes are documented.

The documentation should take place on an ongoing basis and in connection with the research continuation with, for example, analyzes and other processing of data and / or materials being documented on the same day as they are performed.

Additional information and documentation that could be available is, for example, correspondence and notes of importance to the research, as well as reports and publications.

For ongoing research, the following must be documented:

- Background and purpose
- Materials and methods (includes even processing and analysis of data)
- Results (raw and processed data)
- Conclusions

In order to be able to reuse research data, this should be described with metadata. Metadata can, for example, include information about the method used to collect data, definitions of variables, units of measurement, any assumptions, formats and file types for data and software used to collect and / or process data.

Research documentation must be kept for as long as the corresponding data is saved, at least ten years after publication or completion of the project, but in several cases longer than that (if data is saved).

It is important that the formats used for documentation can be read during all the time they are saved. If necessary, these may need to be transferred to archive-proof formats for long-term storage.

6 Data management

Data management is a collective term for the researcher's work with his data. It is important that research data is clearly described, is traceable, handled securely and that it is easy to connect, for example, information in publications to the underlying data. All data that is collected, measured or created within a research project is counted as research data. Data can be numeric, text, images, video or audio recordings. If data is not initially collected in a standard format, then data may need to be migrated to a persistent format prior to long-term storage and archiving.

It is important that the FAIR (Findable, Accessible, Interoperable and Reusable) principles are taken into account when planning data management.

KI recommends that all research projects use data management plans to facilitate good data management. It is the responsible researcher who must develop and continuously update the data management plan if necessary. KI offers a web-based system for electronic data management plans.

A data management plan usually contains a description of data, responsibilities and resources and information on how the research is documented and how the researcher ensures data quality, storage and backup and takes into consideration legal and ethical aspects. The data management plan also describes strategies / plans for making data available and long-term data preservation.

6.1 Active data

At an early stage in the project, the research group should agree on how files should be named and how these should be stored. If this happens in different folders, the naming of the folders and their hierarchy should also be uniform and consistent in order to facilitate both traceability and daily work.
It is important that it is clear which version of a file is the latest and that it is be possible to download previous versions if necessary. Changes can be indicated in the file name and for important documents a version table can be included with the date and note of changes for each version number.
It is important, if possible, to choose the file format that is best suited for long-term durability and availability. Ideally, the file formats should be common and vendor-independent. If necessary, the original file formats may need to be transferred to archive-proof formats prior to long-term storage and archiving.

A file name can consist of the following parts:

• A prefix that shows the type of document a file is
• A title: this should be as informative as possible so that the document content can be understood from its title
• The version number of the document
• Date displayed when created: use YYMMDD format
• Document status: draft or final
• Author's initials: who last changed it

6.2 Storage, sharing and archiving of data

Data must be available to qualified researchers and protected against access by unauthorized persons.

It is important to use approved systems to store data. A current list of approved systems can be found on the employee portal. USB sticks, private computers and external hard drives that are not regularly backed up may not be used within the framework of KI's research projects.

When sharing data files, secure systems must be used. If data to be shared with partners outside KI contains personal data, agreements governing access to the data must be drawn up.

Original data from the implementation of the research project must be saved for at least ten years or longer after publication or completion of the project. Samples should be saved for the same period of time if possible.

Guidance on which research data is to be preserved and for how long, as well as what can be discarded is described in the document management plan for KI.

6.3 Making research data available

Research data must be as open and accessible as possible. This also applies to other data that is part of the research, such as program code and scripts.

If the data contains personal data that can be traced directly or indirectly to a living person, these must not be made directly openly available. In order to also be able to provide access to this data, metadata is published openly and then regulated access to data containing personal data is required.

There may also be other reasons for confidentiality than personal data, for example linked to agreements and / or patents, which prevent data from being made publicly available.

7 Research support

Support via Research Data Office (RDO, rdo@ki.se) focuses specifically on research documentation and data management, such as storage, archiving and making data available.

8 Links

More detailed information, links and help can be found on RDOs homepage.
